# Integrated systems analysis reveals a molecular network underlying autism spectrum disorders

**DOI:** 10.15252/msb.20145487

**Published:** 2014-12-30

**Authors:** Jingjing Li, Minyi Shi, Zhihai Ma, Shuchun Zhao, Ghia Euskirchen, Jennifer Ziskin, Alexander Urban, Joachim Hallmayer, Michael Snyder

**Affiliations:** 1 Department of Genetics, Stanford Center for Genomics and Personalized Medicine, Stanford University School of MedicineStanford, CA, USA; 2 Department of Pathology, Stanford University School of MedicineStanford, CA, USA; 3 Department of Psychiatry & Behavioral Sciences, Stanford University School of MedicineStanford, CA, USA

**Keywords:** autism spectrum disorders, corpus callosum, functional modules, oligodendrocytes, protein interaction network

## Abstract

Autism is a complex disease whose etiology remains elusive. We integrated previously and newly generated data and developed a systems framework involving the interactome, gene expression and genome sequencing to identify a protein interaction module with members strongly enriched for autism candidate genes. Sequencing of 25 patients confirmed the involvement of this module in autism, which was subsequently validated using an independent cohort of over 500 patients. Expression of this module was dichotomized with a ubiquitously expressed subcomponent and another subcomponent preferentially expressed in the corpus callosum, which was significantly affected by our identified mutations in the network center. RNA-sequencing of the corpus callosum from patients with autism exhibited extensive gene mis-expression in this module, and our immunochemical analysis showed that the human corpus callosum is predominantly populated by oligodendrocyte cells. Analysis of functional genomic data further revealed a significant involvement of this module in the development of oligodendrocyte cells in mouse brain. Our analysis delineates a natural network involved in autism, helps uncover novel candidate genes for this disease and improves our understanding of its molecular pathology.

See also: **C Auffray** (December 2014)

## Introduction

Genetic studies of autism spectrum disorders (ASDs) in the past decade have implicated a large number of clinical mutations in more than 300 different human genes (Basu *et al*, 2009). These mutations account for very few autism cases, suggesting that the genetic architecture of autism is comprised of extreme locus heterogeneity (Abrahams & Geschwind, 2008). Key issues in understanding the underlying pathophysiology of ASDs are identifying and characterizing the shared molecular pathways perturbed by the diverse set of ASD mutations (Bill & Geschwind, 2009; Berg & Geschwind, 2012).

The common approach to uncover pathways underlying ASD is based on enrichment tests against a set of annotated pathways for mutations derived from a genome-wide comparison between cases and controls. For example, a β-catenin/chromatin remodeling protein network showed enrichment for the *de novo* mutations identified from sequencing exomes of sporadic cases with autism (O'Roak *et al*, 2012). Common variants from genome-wide association studies (GWAS) were also tested against KEGG pathways, suggesting a possible association with a pathway for ketone body metabolism (Yaspan *et al*, 2011). However, in spite of extensive efforts by many research groups worldwide, including recent large-scale genotyping and sequencing studies (Anney *et al*, 2012; Liu *et al*, 2013), we still lack a complete understanding of the genetic underpinnings of this disease. Therefore, instead of searching genome-wide, we decided that a focused study either by injecting our prior knowledge or by utilizing information from molecular studies of natural pathways might help discover pathways relevant to ASD etiology. Gilmen *et al* constructed a network by connecting every pair of genes with any functional association, such as shared annotation terms, pathway memberships, interacting partners or co-evolutionary patterns. This association network was then seeded with the genes previously found in ASD-associated *de novo* copy number variants (CNVs) followed by a search of their neighborhoods for sub-networks most enriched for these affected genes. This “seeding-and-expansion” strategy identified functionally associated genes in synapse development, axon targeting and neuron motility (Gilman *et al*, 2011). Related studies were focused on a set of proteins potentially implicated in ASD and characterized their interacting partners to identify molecular pathways underlying ASD (Sakai *et al*, 2011; Corominas *et al*, 2014; Cristino *et al*, 2014). These approaches all started with a set of previously curated ASD-associated genes, which served to define an ASD-related framework. However, given our incomplete understanding of ASD, identifying ASD-associated pathways purely based on these known genes might not be able to reveal the “natural” organization of genes implicated in this disease and may miss many components involved in ASD.

A complementary approach was also developed recently, in which human genes were first grouped based on their expression profiles across brain developmental stages or anatomical brain sections. Significant mutation or aberrant expression within a few co-expressed gene groups should then reveal a more complete functional organization underlying ASD (Voineagu *et al*, 2011; Ben-David & Shifman, 2012; Parikshak *et al*, 2013; Willsey *et al*, 2013). However, co-expression analysis often identifies a large number of genes co-expressed for many reasons, including gene sub-cellular co-localization, co-evolution or just coincidental expression, and thus, it is not possible to infer the exact *physical* organization of genes in ASD from such a heterogeneous co-expression network. For example, when we considered a threshold of Pearson's correlation of 0.7 for genes expressed across brain anatomical sections (Hawrylycz *et al*, 2012), more than 2.8 million gene pairs displayed significant co-expression, whereas the complete physical interactome in human is estimated to consist of 150k to 370k protein–protein interactions (Hart *et al*, 2006), accounting for only ∼5–10% of the co-expressed genes. Therefore, co-expression analyses reveal functional association between genes, but not “physical” organization; however, the latter is crucial for delineating the mechanistic basis of the disease.

Herein, we describe a systems biology approach (Supplementary Fig S1) to unravel natural organization of physically interacting proteins implicated in ASD. We analyzed the human protein interactome to detect a protein module strongly enriched for biological processes relevant to ASD etiology. The module is frequently mutated in patients with autism, which was further validated in a large patient cohort and by our own independent sequencing studies. Network and transcriptome analyses of this ASD module collectively revealed that the corpus callosum is likely a potential tissue of origin underlying ASD, in line with its morphological alterations that have been described in patients with ASD (Boger-Megiddo *et al*, 2006; Frazier *et al*, 2012).

## Results

### Modularization of the human protein interactome

**Figure 1:**
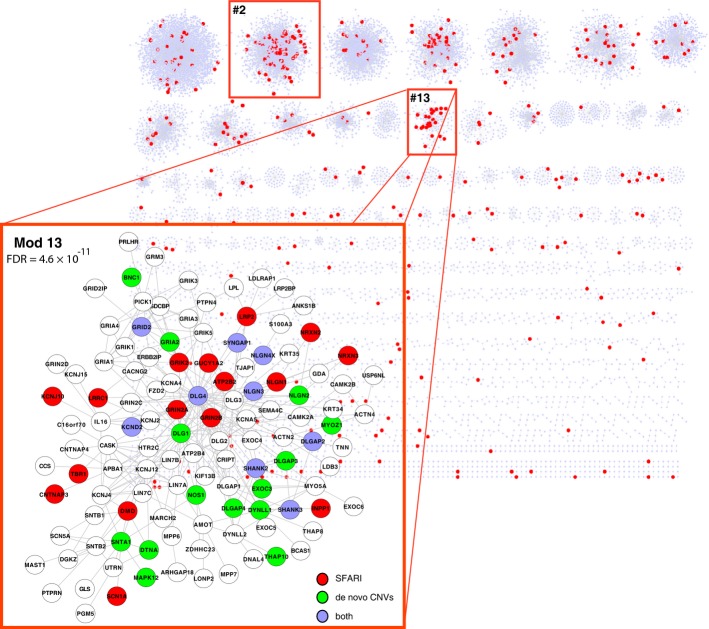
A modular protein interaction network with modules containing enrichment of autism-associated genes Two topological modules (#2 and #13) on human protein interaction network showed significant enrichment for autism genes (in red). The topological modules are physical clusters on the network where their member genes intensively interact with each other but sparsely interact with non-member genes on the network. A zoom-in view of module #13 is also shown, where known autism genes and genes affected by ASD-associated de novo CNVs were colorized in red and green, respectively. Genes annotated by both were in blue. The false discovery rate indicates its significant enrichment for the known autism genes.

We first generated a new topological protein interaction network using the most comprehensive human protein interactome from BioGrid (Stark *et al*, 2011) comprising 13,039 proteins and 69,113 curated interactions (see Materials and Methods, and Supplementary Dataset S1). Since interacting proteins are presumably co-expressed, the quality of these protein interactions was often analyzed by co-expression analysis (Yu *et al*, 2008). We found significantly increased gene co-expression from this dataset relative to a set of previously benchmarked interacting proteins (Das & Yu, 2012) and also to randomly paired proteins (Supplementary Fig S2, and also see Materials and Methods, *P* < 1e-10, Wilcoxon rank-sum test), demonstrating high quality of this human protein interactome dataset. We then topologically clustered the proteins that constituted the network into highly interacting modules using a parameter-free algorithm (Materials and Methods) that was specifically designed for detecting community structures in a large-scale network (Blondel *et al*, 2008). By maximizing the score for network modularity, the human interactome was decomposed into 817 topological modules (Fig1, Supplementary Dataset S1) of non-uniform sizes (Supplementary Fig S3A). Within each module, the proteins tightly interacted with each other, but sparsely with proteins in other modules. This observed modularity of the human interactome was then tested against a set of shuffled networks of the same size by randomly rewiring existing interactions while maintaining the same number of interacting partners. None of the randomized networks achieved the same modularity observed from the network in this study (Supplementary Fig S3B), confirming the significance of these topological clusters (*P* < 0.01, estimated from the 100 random shufflings).

Gene Ontology (GO) enrichment analysis for the 192 topological modules containing more than five genes (Supplementary Fig S4) revealed 85 modules that showed significant enrichment for at least one GO term (FDR < 0.05, hypergeometric test, Supplementary Dataset S2). The enrichment was highly significant for most of the modules (FDR ≤ 5e-3, Supplementary Fig S4), including module #22 for histone acetylation (FDR = 5.3e-3), module #4 for kinase cascades (FDR = 9.41231e-18), module #2 for DNA-dependent regulation (FDR = 2.43e-237) and module #13 for synaptic transmission (FDR = 2.77e-28). Overall, these observations revealed the modular architecture of the human protein interactome, with different modules organized for specific functions (Supplementary Fig S5).

### A protein interaction module is associated with autism

To determine whether any of the modules are related to autism, we first examined the 383 genes involved in ASD susceptibility from the SFARI Gene list (https://gene.sfari.org/autdb/) that were present in the network. Enrichment tests for each module in the network revealed that module #2 (1,430 member genes, FDR = 2.3e-3, hypergeometric test) and #13 (119 member genes, FDR = 4.6e-11, hypergeometric test) showed significant enrichment. Module #2 was enriched for transcriptional regulation, including ASD-associated transcription factors and chromatin remodelers (FOXP2, MECP2, and CHD8, *etc*.), and module #13 encompassed many genes for synaptic transmission (SHANK2, SHANK3, NLGN1, NLGN3, etc., see GO enrichment test above and also in Supplementary Fig S6). Given the substantially stronger enrichment for SFARI ASD genes in module #13 relative to module #2, in the remaining part of the study, we focused on module #13 for its ASD implication and molecular function.

To determine that the observed enrichment for SFARI genes was not biased by unequal CDS (coding DNA sequences) length and GC content in the above comparison, we further performed 10,000 sets of permutation tests. In each permutation, we randomly sampled genes with indistinguishable CDS length and GC content from the SFARI genes (see Materials and Methods), and we validated the enrichment for SFARI genes in module #13 (*P* < 1e-5). The SFARI reference ASD gene list, although comprehensive, is likely to have potential curation bias. We therefore tested this module's enrichment for ASD candidate genes using a variety of validation tests. We first tested whether the observed enrichment for ASD genes in module #13 was simply accounted for by its overall enrichment for synaptic genes. Of the total 1,886 known synaptic genes from SynaptomeDB (Pirooznia *et al*, 2012), 1,745 were present on the network. After removal of the synaptic genes from module #13, ASD non-synaptic genes were highly enriched in the module relative to those in the entire network or across the genome (14.8% versus 2.6% and 2.9%, respectively; *P* ≤ 1.64e-4, hypergeometric test). Furthermore, 5.44% (95/1745) of the ASD SFARI genes were in the synaptic set for the entire network, but 21% (25/119) were in module #13, a highly significant enrichment (*P* = 3.28e-8, Fisher's exact test, for the ratio difference from the synaptic gene set). These comparisons collectively demonstrate that the ASD enrichment in module #13 cannot be attributed to only the synaptic genes in this module, but instead is due to a clustering of ASD genes in the module. Furthermore, the enrichment was also observed when testing ASD genes from different releases of the SFARI curated database (*P* ≤ 1e-10, Supplementary Fig S7).

We next analyzed the association of module #13 with ASD using data from several unbiased genomic studies (Supplementary Dataset S2). To account for any potential bias in CDS length or GC content, all comparisons were based on a set of 9,782 genes with comparable CDS length and GC content with genes in module #13 (*P* = 0.25 and 0.14, respectively, Wilcoxon rank-sum test, see Materials and Methods). We performed five independent tests using (i) all the genes whose exons were affected by *de novo* CNV events from three independent studies (Levy *et al*, 2011; Sanders *et al*, 2011; Pinto *et al*, 2014); (ii) a list of 203 high-confidence genes affected by ASD-associated *de novo* CNVs detected in 181 individuals with autism (Noh *et al*, 2013); (iii) 407 genes affected by rare CNV events associated with ASD (Pinto *et al*, 2010); (iv) 67 genes affected by *de novo* loss-of-function mutations in ASD probands; (v) 366 genes affected by *de novo* missense mutations in ASD probands. As control gene sets for these analyses we also included the following: (vi) 557 genes whose exons were affected by *de novo* CNVs identified from non-ASD individuals (Kirov *et al*, 2012) or unaffected siblings (Levy *et al*, 2011; Sanders *et al*, 2011); (vii) 109 genes with *de novo* missense mutations identified in unaffected siblings; and (viii) 148 and 52 genes with *de novo* silent mutations in ASD probands and unaffected siblings, respectively. All of the above *de novo* point mutations were from recent large-scale exome-sequencing studies (Neale *et al*, 2012; O'Roak *et al*, 2012; Sanders *et al*, 2012). The exact comparisons are shown in Supplementary Table S1A and B.

We observed that genes affected in ASD patients by the *de novo* CNVs (19.33% in the module versus 11.27% in the matched control gene set, *P* = 0.01, Fisher's exact test), the rare CNVs (5.04% in the module versus 2.17% in the matched control gene set, *P* = 0.048, Fisher's exact test) and the disruptive mutations (2.52% in the module versus 0.54% in the in the matched control gene set, *P* = 0.03, Fisher's exact test) each displayed a significant enrichment for this module, whereas the enrichment signal was absent from all types of mutations identified from non-ASD individuals and unaffected siblings, nor the silent mutations from ASD probands (*P* > 0.1, Fisher's exact test, See Supplementary Table S1A and B for the exact comparisons). Notably, although all ASD cohorts were enriched, the strongest enrichment signal was from the high-confidence CNV genes in ASD patients (Noh *et al*, 2013), where 14.29% of these genes were implicated in this module compared with 1.1% in the matched background (*P* = 3.1e-13, Fisher's exact test). Lastly, the similar enrichment was also observed from a set of ASD-associated genes with syndromic mutations, or highly replicable genes in different GWAS patient cohorts (*P* = 3.85e-6, Fisher's exact test, scored by SFARI Gene Module, category “S”). Overall, both curated data and data from genome-wide screening consistently support a significant association of module #13 with ASD. Our own sequencing as described in the section below provides further evidence for this module's involvement in ASD.

Module #13 was also more enriched for ASD genes (21% in the module) than genes involved in schizophrenia (Jia *et al*, 2010) (10% in the module) and intellectual disability (Parikshak *et al*, 2013) (9.2% in the module), whereas no enrichment was observed for Alzheimer's disease (Bertram *et al*, 2007) (*P* = 0.28, Fisher's exact test, see Materials and Methods). The increased overlap with schizophrenia and intellectual disability relative to Alzheimer's disease was expected given the shared molecular etiology among the psychiatric disorders (Lee *et al*, 2013). Overall, this comparison suggests that the module is likely more specific toward ASD-related genes.

### DNA sequencing of ASD patients reveals an enrichment of rare non-synonymous mutations in this module

We sequenced postmortem brain DNAs collected from 25 ASD patients (all Europeans, Supplementary Table S2); in 19 subjects, we sequenced the whole exomes (WES, >97× coverage) and in six the whole genomes (WGS, ∼35–40× coverage). In addition, we sequenced four genomes and one exome from non-autistic European individuals to control for the overall sequencing quality (see Supplementary Tables S2, S3 and S4). We first analyzed variants identified from the WES platform (19 exomes) and identified 153 non-synonymous variants that were mapped onto the module, among which 19.6% (30/153) were extremely rare and were not previously observed in the 1,000 Genome dataset. Randomly sampling the same number of genes 10,000 times, with indistinguishable CDS length and GC content from those in this module, demonstrated a significant enrichment for the rare non-synonymous variants in this module (*P* = 1.2e-3, with the expected fraction 12%). The same enrichment signal was also observed from the variants identified by WGS (*P* = 2.5e-3, permutation test).

**Figure 2:**
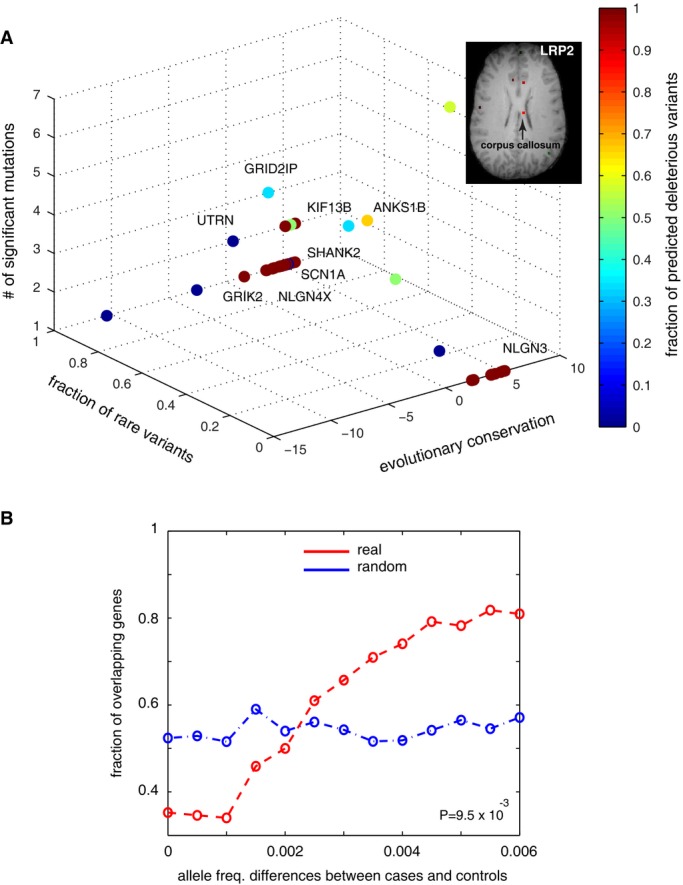
Candidate genes from sequencing screens An overview of the identified loci from whole-genome and exome sequencing. Evolutionary conservation is quantified by GERP++ score, where the higher scores indicate greater selective pressure on the genomic loci. For genes with multiple significant loci, the most conserved residue is considered. Variants absent in the 1,000 Genome dataset are considered rare variants. The genes were colorized based on the fraction of deleterious mutations predicted by MutationTaster among all the identified mutations in the gene (MRI image of the corpus callosum: Allen Institute of Brain Science).
Validation using another larger patient cohort. In this dataset, variants with allele frequencies with increased absolute differences between cases and controls are more likely to affect genes that were also detected in our study (red line). The allele frequency difference is the absolute difference between cases and controls. This trend cannot be observed by 10,000 simulations (blue line for one randomized dataset).

Excluding the variants also identified in the control subjects that were sequenced on the same platform, we considered 113 non-synonymous sites in this module collectively identified from WGS or WES. We compared their allele frequencies to those in the 1,000 Genomes dataset, both the entire global populations and the European populations, and from the 25 patients, we identified a total of 38 genes affected by significant non-synonymous variants in this module with an expected false-positive rate at 0.1 (determined by Fisher's exact test followed by Benjamini–Hochberg correction). The high gene overlap between WGS and WES was not expected by chance (*P* = 0.03 by random permutation test). Furthermore, the identification of genes in our module was not affected by the CDS length of the identified genes relative to the average CDS length in the module (*P* = 0.16, Wilcoxon rank-sum test). The identified genes and a summary of the variant information are shown in Fig2A. For example, LRP2 harbored seven distinct non-synonymous mutations (z-axis, Fig2A), four of which were predicted to be deleterious by MutationTaster (Schwarz *et al*, 2010). LRP2 has recently been identified as an ASD candidate gene (Ionita-Laza *et al*, 2012), whose clinical mutations cause the Donnai–Barrow syndrome (Kantarci *et al*, 2007) with the underdeveloped or absent corpus callosum. This syndrome exhibits many autistic-like symptoms. Figure2A further underlines its tissue specificity in the corpus callosum using Brain Explorer (http://www.brain-map.org). Other well-characterized ASD-associated genes included SHANK2, SCN1A, NLGN4X and NLGN3 as well as several LRP2 interacting proteins (LRP2BP, ANKS1B). Overall, the affected loci in these genes were more likely to be both rare in the population (*y*-axis) and evolutionarily conserved (*x*-axis), suggesting their functional importance (Fig2A). We also noted that 28 genes of the 38 ASD candidates have not been described previously (see Supplementary Dataset S3). To better support their association with this disease, we further examined their mouse mutant phenotypes in Mouse Genome Informatics (http://www.informatics.jax.org) and observed that 10 of the 28 new candidate genes displayed abnormal behavioral traits or a defective nervous system in their respective mouse mutants (see Supplementary Dataset S3). For example, mouse mutants of (i) ANKS1B and KCNJ12 exhibited hyperactivity, (ii) ERBB2IP hyporesponsive behavior to stimuli, (iii) GRID2IP abnormal reflex and (iv) SCN5A seizure.

### Validation using an independent patient cohort

We next sought to further validate our observations in a larger patient cohort. An exome-sequencing dataset of 505 ASD cases and 491 controls, each of European ancestry and unrelated within the cohort, was analyzed (Liu *et al*, 2013). These samples had been sequenced using a separate sequencing platform (SOLiD), and the patients did not overlap with our sequenced cohort (See Materials and Methods). A previous study examined this dataset but did not find any genes (or variants) significantly associated with ASD (Liu *et al*, 2013). We compared the allele frequencies for each of non-synonymous variant detected in this study and found ∼95% of these variants had case–control frequency differences below 0.8%. We observed that genes with non-synonymous variants with the highest allele frequency differences between cases and controls were more likely to be in the 38 module-specific candidate genes that we identified in our sequencing cohort (Fig2B), and this trend was not observed when we randomly sampled the same number of genes from the module for 10,000 times (*P* = 9.5e-3, Fig2B). Furthermore, regression analysis on this dataset identified 16 genes in this module with the extreme imbalanced allele frequencies among the patient population (*P* < 0.05, see Materials and Methods); 14 were in the 38 candidate genes we identified (*P* = 1.2e-6, hypergeometric test, Supplementary Dataset S3). Thus, this large-scale exome-sequencing data validated and extended our results.

### Expression specificity of the module in the corpus callosum

We next examined expression of the genes in module #13 using the Allen Human Brain Atlas (Hawrylycz *et al*, 2012), which describes the spatial gene expression across hundreds of neuroanatomically precise subdivisions as measured by microarray analyses of two individuals. Since the individuals exhibited high concordance in expression profiles across brain sections (Hawrylycz *et al*, 2012), we averaged the gene expression data for each of the 295 anatomical brain sections.

**Figure 3:**
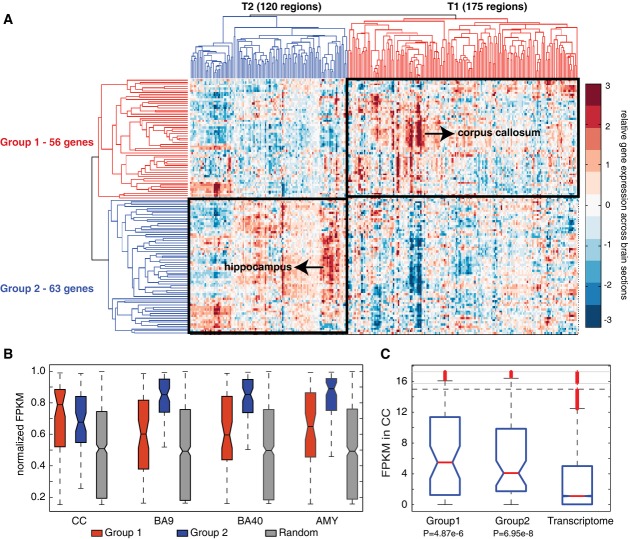
Expression analysis of the synaptic module Dichotomized expression of the genes in module #13 across 295 brain sections. Relative abundance of each gene across the 295 brain sections was hierarchically clustered to reveal gene groups exhibiting similar expression patterns across tissues. Group 1 genes showed elevated expression in 175 regions (T1, e.g., corpus callosum) relative to other brain sections, and Group 2 genes showed high expression in 120 regions (T2, e.g., hippocampal regions) relative to other brain sections.

Most genes in module #13 were expressed across all brain sections (Supplementary Fig S8). However, hierarchical clustering of the normalized gene expression across brain sections revealed two distinct spatial patterns with some heterogeneity apparent in each (Fig3A, complete list in Supplementary Dataset S4). Group 1 had 56 of 119 total genes preferentially expressed in 175 regions (T1 regions in FigA), whereas the 63 genes of Group 2 had elevated expression in the other 120 brain regions (T2 regions in Fig3A). Group 1 genes were strongly expressed in sections associated with the corpus callosum (Fig3A, including LRP2 shown in Fig2A), which transfers motor, sensory and cognitive signals between the brain hemispheres. Group 2 genes (e.g., SHANK2 and SHANK3) were up-regulated in T2 regions, which encompassed neuron-rich regions, exemplified by the hippocampal formation, including CA 1/2/3/4 fields, subiculum and dentate gyrus. Tissue enrichment was derived from *relative* expression of individual genes across brain sections; closer examination of their *absolute* expression in each brain section relative to the transcriptome background revealed that Group 1 expression levels were at background levels across most tissue types, but peaked in the corpus callosum (Supplementary Fig S8). Group 2 genes were highly expressed across all tissues, albeit their expression levels were slightly depressed in the corpus callosum (Supplementary Fig S8). Thus, Group 2 genes were more ubiquitously expressed, and Group 1 genes were tissue specific in the corpus callosum, and the trend was evidenced by its increased tissue specificity index (*P* = 1.5e-4, Wilcoxon rank-sum test) and decreased expression breadth (*P* < 0.01, Wilcoxon rank-sum test, Supplementary Fig S9).

RNA-sequencing of four different brain regions from a healthy subject. The brain regions include the Brodmann areas 9 (BA9), 40 (BA40), the amygdala (AMY) and the corpus callosum (CC), which revealed the same observation as from the microarray analyses. Group 1 (red) and 2 (blue) genes were compared with 1,000 randomly sampled genes (gray) from the transcriptome in each brain region. The raw FPKM values were normalized into the cumulative density functions based on kernel density estimation. The elevation of Group 2 genes across all brain regions and the greatest increase of Group 1 genes in the corpus callosum were all statistically significant (*P* < 1e-5, Wilcoxon rank-sum test).

RNA-sequencing of the corpus callosum transcriptomes from six non-autistic individuals. FPKM quantifies the absolute expression of genes in each group. The two groups have similar expression in the corpus callosum (*P* > 0.5, Wilcoxon rank-sum test), which are all above the transcriptome background (*P* < 4.87e-6, Wilcoxon rank-sum test), suggesting that both sub-components are active in this tissue.

We further tested the tissue specificity of expression patterns by RNA-sequencing (RNA-Seq) of postmortem human brain samples in two sets of experiments. First, we examined expression levels in four brain regions of one individual with no known disease (see Materials and Methods). These regions were the dorsolateral prefrontal cortex (Brodmann Area 9, BA9), the parietal lobe (Brodmann Area 40, BA40), the amygdala (AMY) and the corpus callosum (CC). BA9, BA40 and AMY are neuron-rich regions, while the corpus callosum is glial rich. Consistent with the microarray results, Group 2 genes were highly expressed in all tissues (*P* < 8e-7, Wilcoxon rank-sum test, FigB) confirming their ubiquitous expression, and Group 1 genes showed the greatest up-regulation over the average transcriptome background in the corpus callosum (*P* < 1.6e-6, Wilcoxon rank-sum test, Fig3B) confirming their increased tissue specificity. These RNA-Seq experiments also confirmed the tissue specificity of LRP2 in the corpus callosum (Supplementary Fig S10), as expected from Fig2A. Secondly, to rule out individual variability, we also examined gene expression by RNA-Seq of the corpus callosum from six normal individuals (all young Caucasian males; the control subjects in our later RNA-Seq experiments, Materials and Methods). We found that both Group 1 and 2 genes were highly expressed in the corpus callosum relative to the transcriptome background (*P* < 4.87e-6, Wilcoxon rank-sum test, Fig3C). These results confirmed that module #13 as a whole is highly expressed in the corpus callosum, the largest white matter structure in human brain.

**Figure 4:**
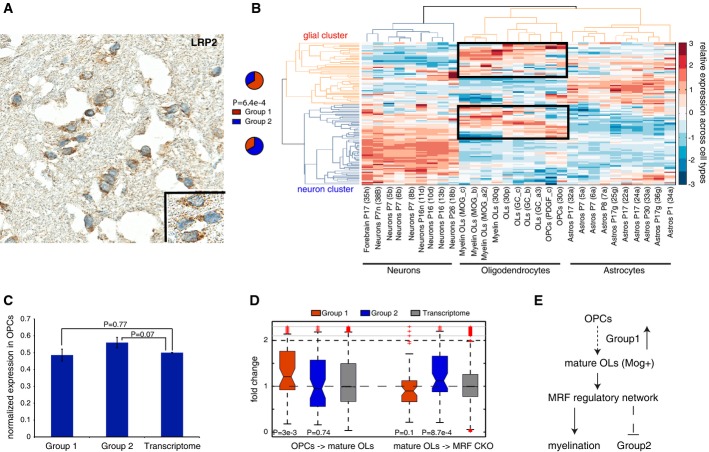
Cell-type expression of module #13 in oligodendrocytes Immunohistochemistry analysis in the corpus callosum. Staining of LRP2 in the human corpus callosum reveals that the major cell population in the corpus callosum is the oligodendrocytes (the blue round nuclei), which express LRP2 stained in brown. A zoom-in view is shown in the inset.

To further validate our results, we performed immunohistochemical analyses for a Group 1 corpus callosum-specific gene (Supplementary Fig S10), LRP2, that also showed excessive mutation in our sequencing analyses (FigA). The experiment was performed in the frozen postmortem corpus callosum tissue from one autism patient (Fig4A) and one control subject (Supplementary Fig S11). LRP2 protein was significantly expressed in the corpus callosum in both individuals, with no obvious difference between the normal and ASD subjects. As shown in Fig4A, the staining results further revealed that the human corpus callosum was predominantly populated by oligodendrocyte cells.

Neural cell-type expression of the orthologous module #13 in mouse brain. Gene expression in different neural cell types was hierarchically clustered into the three major cell types in brain (neurons, oligodendrocytes and astrocytes). The clustering grouped genes in module #13 into a neuron cluster and a glial cluster, enriched for Group 1 and 2 genes, respectively. The fraction of Group 1 (red) and 2 (blue) genes in the glial and neuronal clusters were represented by the pie charts, with statistical significance determined by a chi-square test.

Overall expression of module #13 in cultured oligodendrocyte precursor cells (OPCs). Group 1 and 2 were expressed at a similar level as the transcriptome background in OPCs. The statistical significance was determined by Wilcoxon rank-sum test, and the error bars represent one standard error.

The role of the module in oligodendrocyte (OL) development. Differentiation of OPCs into mature myelinating OLs (MOG^+^) led to a significant up-regulation of Group 1 genes (left, OPCs → mature OLs). On the other hand, conditional knockout (CKO) of the master myelination factor MRF from mature OLs led to a significant up-regulation of Group 2 genes (right, mature OLs → MRF CKO). The statistical significance was determined by Wilcoxon rank-sum test.

A proposed model. Up-regulation is associated with, or likely to contribute to, the differentiation of OPCs into mature myelinating OLs. The mature OLs acquire their myelination capacity by activating the MRF-mediated regulatory network, which also serves to repress expression of Group 2 genes.

Given this fact, we next explored the function of this module in the oligodendrocytes by comparing gene expression of module #13 with other major cell types (neurons and astrocytes) in brain. Due to a lack of the cell-type expression data in human brain, we mapped module #13 onto their unambiguous mouse orthologs (the one-to-one orthology) and analyzed their cell-type expression (Cahoy *et al*, 2008). Hierarchical clustering revealed that the mouse orthologs in our module formed two major clusters with expression enrichments in either neurons or glial cells (i.e., oligodendrocytes and astrocytes, Fig4B). The expression profiles of glial cells were significantly enriched for Group 1 genes, and of neuronal cells for Group 2 genes (*P* = 6.4e-4, chi-square test, Fig4B), suggesting that expression propensities of Group 1 and 2 in sections T1 and T2 (Fig3A), respectively, were largely due to their different compositions of glial cells and neurons. However, a portion of the genes in both the neuron and glial clusters showed common enrichment in the oligodendrocytes, separating the cluster of the myelinating oligodendrocytes (myelin OLs, the sub-cluster on the *x*-axis, Fig4B) from the non-myelinating oligodendrocytes (the newly differentiated oligodendrocytes, OLs and the oligodendrocyte precursor cells, OPCs, the sub-cluster on the *x*-axis, Fig4B). We thus hypothesized that the two sub-components (Group 1 and 2) in the module are likely to be involved in the development of oligodendrocyte cells.

Using the data generated by Emery *et al* (2009), we next compared gene expression of the mouse orthologs of Group 1 and 2 genes in differentiating mouse culture systems. In cultured oligodendrocyte precursor cells (OPCs), the two gene groups did not show substantial expression changes relative to the transcriptome average (Fig4C). However, in the matured myelinating oligodendrocytes (MOG^+^), Group 1 genes exhibited marked up-regulation (*P* = 3.0e-3, Wilcoxon rank-sum test, Fig4D), whereas the Group 2 genes showed slight down-regulation with no statistical significance (*P* = 0.74, Wilcoxon rank-sum test). This indicates that up-regulation of Group 1 genes is associated with oligodendrocyte maturation.

In the same mature oligodendrocytes, we tested the expression of module #13 components using mouse knockouts (Emery *et al*, 2009). The transcription factor, myelin gene regulatory factor (MRF), plays a central role in developing myelination capacity for oligodendrocyte cells, and mice lacking MRF in the oligodendrocyte lineage show defects of myelination, accompanied by severe neurological abnormalities and postnatal lethality due to seizures (Emery *et al*, 2009). In mouse oligodendrocytes with a conditional knockout of MRF (MRF^fl/fl^; Olig2^wt/cre^), Group 2 genes exhibited a significant up-regulation relative to the transcriptome background (*P* = 8.7e-4, Wilcoxon rank-sum test, Fig4D), whereas Group 1 genes underwent down-regulation with marginal statistical significance (*P* = 0.1, Wilcoxon rank-sum test, Fig4D). This suggests that Group 2 genes are directly or indirectly suppressed by the master myelination factor MRF in the myelinating oligodendrocytes. Overall, given these observations, we propose that up-regulation of the Group 1 genes in this module is associated with, or likely contributes to, oligodendrocyte maturation from their precursor cells (OPSc). However, in the mature oligodendrocytes, myelination capacity is acquired by the MRF-mediated regulatory network, which also serves to suppress expression of the Group 2 genes (Fig4E).

### Altered gene expression in the corpus callosum of ASD patients revealed by RNA-sequencing

**Figure 5:**
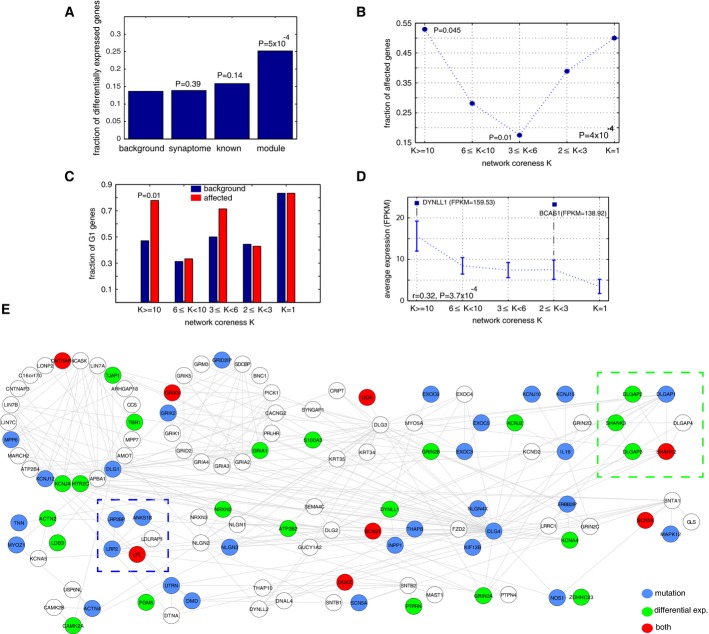
Integrative analysis of the genetic alteration in this study Enrichment of the differentially expressed genes in module #13. RNA-sequencing of the corpus callosum of autism patients and their matched controls. Enrichment was not observed for the genes in the human synaptome or the collection of known autism genes (excluding genes in this module). Statistical significance was determined by hypergeometric test.

Given the apparent importance of oligodendrocytes in the corpus callosum, we further hypothesized that gene expression in this module is likely to be perturbed in the corpus callosum of ASD patients. We obtained frozen postmortem samples from six young Caucasian males with a diagnosis of autism together with their respective matched controls from the NICHD Brain and Tissue Bank (Materials and Methods and Supplementary Table S5). Total RNAs were prepared and subjected to high-coverage (180M reads/sample) deep RNA-sequencing. Biological replicates (with the same sequencing depth) were performed on half of the samples, using different sections of the same tissue block. The biological replicates produced highly reproducible results with a median Pearson's coefficient equal to 0.95 (range 0.9–0.96; Supplementary Fig S12), whereas the correlations among samples from different individuals were substantially lower (median correlation coefficient 0.89, *P* = 4.4e-3, Wilcoxon rank-sum test), demonstrating the high intra-individual reproducibility of our platform. Because gene expression in the brain is age dependent in patients with autism (Chow *et al*, 2012), we compared gene expression in each case–control pair with identical age, ethnicity, sex and comparable postmortem intervals (PMIs). We then identified genes showing the most extreme expression changes in at least one case–control pair (fold change > 2, above the 97.5% upper bound for up-regulation and below 2.5% for down-regulation across the entire transcriptomes, Supplementary Table S6). Genes encoding components of the module #13 showed significant enrichment for the differentially expressed genes relative to the genes encoding the entire protein interaction network (*P* = 5e-4, hypergeometric test, Fig5A). We conducted comparisons against two control gene sets: a complete list of 1,886 known synapse-related genes (the synaptome in Fig5A) from SynaptomeDB (Pirooznia *et al*, 2012) and the other control included a list of known 383 autism candidate genes represented on the network. In each case, the gene set contained a similar fraction of differentially expressed genes as the entire transcriptome background (*P* = 0.39 and 0.14, hypergeometric tests, respectively). Thus, expression of module #13, but not synaptic genes in general or known ASD candidate genes, was significantly altered in the corpus callosum of the ASD patients relative to the matched controls.

The mutation pattern of the genes from the innermost layers of the interaction network (*K *≥ 10) to the periphery layer (*K* = 1). Genes in the central and periphery layers in this module are more likely to be affected, while the trend cannot be observed in 10,000 random simulations. For individual bins, significant enrichment and depletion were observed in the central layers (*K* ≥ 10) and the intermediate layers (3 ≤ *K* < 6), respectively. Statistical significance of the enrichment was determined by hypergeometric test. 10,000 random permutations were performed to determine the statistical significance of the curve.

Compositional bias of the mutated genes in central layers. The mutated genes in central layers are more biased toward the corpus callosum-specific subcomponent; this trend is not observed in background or other mutated genes with varying degree of *K*. Statistical significance of the enrichment was determined by hypergeometric test.

Positive correlation between network coreness and gene expression in the corpus callosum. RNA-sequencing of the corpus callosum of six non-autistic individuals revealed a positive correlation, suggesting the central layers may play critical roles in the corpus callosum. Two outlier genes, DYNLL1 and BCAS1, are separately labeled due to their extreme expression in this tissue. The correlation coefficient r and its statistical significance were computed using Spearman's correlation.

Predicted sub-complexes within this module. Genes in this module are topologically clustered to form sub-complexes, with the significantly mutated genes labeled in blue, mis-expressed genes in the corpus callosum labeled in green, and both in red. Two clusters, #6 for SHANK-DLGAP complexes and #6 for LRP2, and its binding partners, are enriched for the mis-expressed or mutated genes, respectively. Statistical significance of the enrichment was determined by hypergeometric test.

### A network view of the candidate loci in this ASD module

We postulated that genes associated with ASD might show common patterns in their topological positions on the molecular network, and thus, we used the protein interaction network to integrate our findings from the genome sequencing and expression analyses for the module. The global interactome can be viewed as a layered structure with proteins distributed from central cores to peripheral layers. This can be revealed by the *k*-core decomposition algorithm (Materials and Methods, also see the layered structure in Supplementary Fig S13), where the coreness *K* of a protein describes its closeness toward the network center. Proteins with *K* = 1 are peripheral nodes that are individually connected, and proteins with *K *≥ 10 lie in the center of the network (the entire *K* distribution is shown in Supplementary Fig S14). A previous study has shown that the proportion of essential and conserved proteins increased successively toward the network's innermost cores (Wuchty & Almaas, 2005).

By combining the 38 genes with at least one significant non-synonymous variant detected from our whole-genome and exome sequencing (Fig2A), we examined the fraction of genes with the significant variants as a function of their coreness *K* in the module. As shown in Fig5B, within this module, a significantly high proportion of central proteins were affected by mutations in individuals with ASD (*P* = 4.5e-2, hypergeometric test), whereas a significant depletion was manifested in the intermediate layer (3 ≤ *K *< 6) (*P* = 0.01, hypergeometric test). The peripheral nodes were also enriched for mutations in the module, but these were not statistically significant. By randomly sampling the same number of genes from the module 10,000 times, we found that the particular U-shape distribution was not expected by chance (*P* = 4.0e-4), suggesting that network topology is indeed correlated with gene mutation frequency in ASD patients.

We also examined brain tissue gene expression as a function of network coreness *K*. Analysis of the different layers of the network revealed that protein products of the genes centered in the network (*K* ≥ 10, Fig5B) were significantly biased toward the corpus callosum-specific sub-component (Group 1; Fig5C, *P* = 0.01, hypergeometric test). These observations were also observed using the independent 500-patient cohort (*P* ≤ 0.05, hypergeometric test). Further analysis of the corpus callosum RNA-sequencing data from the six non-autistic subjects (Supplementary Table S5) revealed a positive correlation between the network coreness and their expression levels for individual genes in module #13 (*r* = 0.32, *P* = 3.7e-4, Spearman's correlation, Fig5D). These observations collectively indicate that the central genes may play fundamentally important roles in the corpus callosum as they are preferentially expressed in this tissue and pathogenic mutations of ASD patients more likely lie in these genes. We note that two genes, DYNLL1 and BCAS1, displayed extreme expression in the corpus callosum (Fig5D) with FPKMs > 130. Examination of their expression in the three neuronal regions (BA9, BA40 and AMY, Fig3B) revealed that DYNLL1 is a ubiquitously expressed gene with high expression across all the brain sections, whereas the extreme expression of BACS1 was unique only in the corpus callosum (FPKM < 20 in other neuronal regions). Its specific expression in the corpus callosum was further confirmed on the microarray data from Allen Brain Atlas, suggesting a novel function of this gene in the corpus callosum.

### Affected sub-complexes in this ASD module

To characterize the module at higher resolution, we decomposed it into 21 sub-clusters (Fig5E) using the algorithm in Fig1. Functional coherence among genes within the same sub-complexes was observed; for example, EXOC3–6 were clustered in the fourth sub-complex, consistent with their co-complex membership by recent mass spectrometry profiling (Havugimana *et al*, 2012). The second sub-complex contained glutamate receptors, encompassing AMPA, kainate and NMDA families, delineating the collaborative nature of these receptor proteins. Most interestingly, many known genes implicated in ASD were also co-clustered, such as the co-clustering of NLGN1-3 with NRXN2-3, suggesting mutations on these genes are likely to perturb a common protein complex. In general, except for one sub-complex (THAP10-DYNLL2-DNAL4), all others have been affected by either mutations or mis-expression of at least one member protein, suggesting a pervasive role of this module underlying ASD etiology. Notably, the sixth and eighth sub-clusters showed significant enrichment for both the differentially expressed genes (*P* = 0.035, hypergeometric test) and the mutated genes (*P* = 0.036, hypergeometric test), respectively (Fig5E). The sixth sub-cluster revealed interaction between the DLGAP (DLGAP1-4) and SHANK proteins, all of which are part of the postsynaptic scaffold. In addition, genes in the eighth sub-complex were preferentially mutated in our screen, which characterized another pathway involving the corpus callosum-specific protein LRP2. Overall, these results further delineate the substructure of the components and complexes that comprise the ASD-associated module.

## Discussion

Most of our knowledge today about ASD genetics has been gained from genetic association or exome-sequencing analyses of large ASD patient cohorts, which allows us to begin to observe the molecular underpinnings of this disease. However, a complete picture for this disease may require an integration of ASD genetic data from different dimensions. For example, a number of studies have analyzed genes that displayed differential expression in ASD brains (Voineagu *et al*, 2011; Chow *et al*, 2012), but aberrant mutations have not yet been identified for many of these genes. Since the retention of genetic mutations within a population is strongly driven by natural selection and population demographics (Hartl & Clark, 2007), mutations in genes critical for ASD are likely to be depleted by purifying selection or simply by population bottleneck, preventing the identification of ASD candidate genes only from mutational analyses. In addition, another example of a gene that would be missed by differential expression studies is LRP2, whose implication in ASD was found by this study and also an earlier investigation (Ionita-Laza *et al*, 2012), but it did not exhibit altered expression in ASD patients. These observations strongly suggest that genetic alterations leading to ASD might occur at different levels, perturbing gene regulation or affecting gene function, and highlight the importance of building an integrative model to study ASD, where genomic data from multiple independent dimensions are incorporated to reveal the hidden architecture of this disease.

The integrative framework presented in this study is such an example to unravel the natural and physical organization of components implicated in ASD. We leveraged abundant genomic data including the human protein interactome, the transcriptome data in human and mouse brain, the MRF knockout data in mouse oligodendrocytes and also the mutation data from previous ASD sequencing projects. In addition, we also independently sequenced the genomes, exomes and transcriptomes in patients’ brains to validate our observations from those publically available data or to gain new insights into this disease. Our integrative approach incorporated these genomic data of diverse dimensions, suggesting several key findings relevant to autism. First, we observed the modular structure of the human protein interactome, where genes forming a natural topological cluster tend to have shared functions. In particular, module #2 (with GO enrichment for gene regulation) and #13 (with GO enrichment for synaptic transmission) showed statistically significant enrichment for ASD genes. Their enriched functional categories are consistent with earlier studies for *de novo* mutations associated with ASD (O'Roak *et al*, 2012; Ben-David & Shifman, 2013). These observations suggest convergent functional modules underlying the seemingly heterogeneous mutations associated with ASD.

Because of its high enrichment, we specifically studied module #13, and a second key finding is that this module had dichotomized spatial expression pattern across human brain: one sub-component (Group 2 genes) ubiquitously expressed and one with enhanced molecular expression in the corpus callosum (Group 1 genes). Both interact extensively with each other. We confirmed using RNA-Seq, microarrays and immunohistochemical staining that the module as a whole was expressed in the corpus callosum, a brain structure predominantly constituted by axons and oligodendrocyte cells. Up-regulation of Group 1 genes was associated with oligodendrocyte maturation from OPC cells (Fig4D). Considering that the expression of Group 1 genes is highly enriched in the corpus callosum, we speculate that this sub-component is likely involved in differentiating OPCs in the corpus callosum. Genes in this group include KCNJ10 (potassium inwardly rectifying channel, subfamily J, member 10), which exhibited tenfold up-regulation from OPCs to the matured myelinating oligodendrocytes, suggesting a strong role of this gene in oligodendrocyte development. Importantly, mutations in this gene were identified among ASD patients from our exome/genome sequencing and also in an earlier study from a different patient cohort (Sicca *et al*, 2011). Meanwhile, aberrant mutations in this gene were also found to be associated with seizure susceptibility (Buono *et al*, 2004), a condition commonly comorbid with ASD. These observations support the potential role of oligodendrocytes in the development of autism. Group 2 genes, in addition to their relatively high expression in the corpus callosum (Fig3C), showed the strongest expression in neuronal regions in brain (Figs3B and 4B), explaining the high enrichment signal of synaptic genes in module #13 in our initial GO enrichment analysis. This observation supports the synaptic theory of this disease.

The corpus callosum plays a central role in mediating signal communication between the brain hemispheres through the axons extending from different cortical layers; thus, appropriate myelination by the oligodendrocytes for the axons is key for the process. We further observed that conditional knockout of the myelination regulatory factor (MRF) in the matured oligodendrocyte cells significantly up-regulated Group 2 genes, which were otherwise highly expressed in neuron-rich regions. Collectively given the functions of module #13 involved in the development of oligodendrocytes, the major cell type in the corpus callosum, our study likely provides a molecular clue to the reduced size of the corpus callosum that has been observed among individuals with ASD (Egaas *et al*, 1995).

Two recent studies (Parikshak *et al*, 2013; Willsey *et al*, 2013) have implicated the superficial cortical layer (II/III) or the deep cortical regions (layer V/VI) in ASD. Callosal projection neurons are primarily localized in the superficial layers II/III (∼80%) or deep layers V/VI (∼20%); thus, our study now connected the two studies suggesting a critical role of the interhemispheric connectivity circuitry, whereby disrupting its sub-components to affect the interhemispheric signal transduction through the corpus callosum will likely to give rise to ASD phenotypes. Therefore, the disease etiology should be understood at the level of the complete interhemispheric connectivity circuitry, not simply by a particular brain region or cell type. This could not only explain the enrichment in ASD-associated mutations in genes highly expressed in the constitutive parts of the circuitry (superficial or deep cortical layers in the earlier studies, or in the corpus callosum in this study), but also might provide a molecular basis for the observation from the imaging studies of the under-development of the corpus callosum among ASD patients. Importantly, different from previous research, our study illustrates the role of the oligodendrocyte cells in ASD, which myelinate and support the axons in the corpus callosum for interhemispheric signal transduction. Since current ASD research has been primarily focused on neuronal regions, future study is warranted to examine the implications of other cell types in this disease.

Two groups of genes were identified previously which displayed elevated expression in the corpus callosum, but were not significantly associated with ASD (Ben-David & Shifman, 2012). The overlap between our module and these genes was restricted to two genes. Meanwhile, only four of our genes overlapped with those from NETBAG (Gilman *et al*, 2011), which identified the functionally associated genes affected by rare *de novo* CNVs in autism. Notably, a more recent paper considered a sub-network implicated in ASD constituted by known ASD candidate genes and their first-degree interacting neighbors (An *et al*, 2014; Cristino *et al*, 2014). This empirical network was large and encompassed more than 2,000 genes for ASD, but ∼30% of genes in our module were not captured by their empirical network. Worthy of note, based on independent yeast-two-hybrid screens, recent studies have attempted to generate the complete interactomes for individual proteins implicated in ASD (Sakai *et al*, 2011; Corominas *et al*, 2014), and thus, we envision a significant expansion of our current observation when the human protein interactome is more complete.

In conclusion, by using an integrative framework, we were able to examine the convergence of clinical mutations onto specific disease-related pathways. The framework provided in this work might be used to uncover functional modules for other diseases, improving their risk assessment.

## Materials and Methods

### Network compilation and operations

The human protein interaction network used in this study was downloaded from BioGrid database (rel.3.1.92) (Stark *et al*, 2011), where high-quality protein interactions were collected by the curation team. We removed the isolated nodes, self-interacting edges and interactions between human and non-human proteins from the network. We analyzed a total of 13,039 proteins and 69,113 interactions. To first assess the quality of this network, we examined gene co-expression between the reported interacting proteins, which has been used previously to examine the quality of protein interactions (Yu *et al*, 2008). We compared gene co-expression between the BioGrid interactome with a set of benchmarked high-confidence human interacting proteins (HINT) (Das & Yu, 2012; Wang *et al*, 2012) and also with a set of randomly paired proteins. The expression dataset encompassing 79 human tissues and cell types (Su *et al*, 2002) was used for the co-expression analysis, where probe identifies from the microarray platform were mapped onto their Entrez identifiers, and signals of multiple probes corresponding to a single Entrez gene were averaged. Pearson's pairwise correlation was then computed for protein pairs in each dataset.

Having assessed the overall quality of the network, we next topologically decomposed the global protein interaction network into a set of network modules with dense interactions within a module and sparse interactions between modules. The network decomposition algorithm was first described in a previous publication, which showed significant improvement compared with other methods (Blondel *et al*, 2008). The modules in this study were from the first-pass partitioning of the network without further grouping small modules into larger ones. This practice gave more specific insights into module functions. The power-law distribution of the module sizes (Supplementary Fig S3A) was based on a statistic test for empirical data (Clauset *et al*, 2009). To test whether the modularity of the network can be observed by chance, we generated 100 randomized networks by shuffling edges of each node but maintained its degree (degree-preserving shuffling (Milo *et al*, 2002)) (Supplementary Fig S3B). We also performed Markov clustering algorithm (MCL) and affinity propagation (Vlasblom & Wodak, 2009) to divide the network, but their performance was not satisfactory, where the resulting network modularity scores *Q* were significantly lower than that of the algorithm used in this study. These network operations were based on FUGA (Drozdov *et al*, 2011). Network visualization was implemented by CytoScape v2.8.3 (http://www.cytoscape.org). The layered structure of the protein interaction network was decomposed with the *k*-core algorithm implemented by MatlabBGL (http://dgleich.github.io/matlab-bgl/). Visualization of the layered structure by *k*-core decomposition was implemented by LaNet-vi (http://lanet-vi.soic.indiana.edu).

We examined GO enrichment for each of the decomposed network module to infer their biological relevance. GO annotations (excluding IEA terms) were downloaded from http://www.geneontology.org (as of Sep. 2012). The hypergeometric test was performed to determine GO enrichment, followed by FDR correction (false discovery rate). In each of the tests, we only considered modules with more than five genes. To justify this size threshold selection, we varied the threshold from 1 to 20 genes and identified *n* = 5 was the optimal threshold, which has balanced sensitivity and specificity (Supplementary Fig S4B). Specifically, in Supplementary Fig S4B, the blue curve (with red circles) showed the number of clusters with GO enrichment above a given size threshold, and the black curve (with green squares) was the gradients of the blue curve at each threshold, which detected the pattern changes on the blue curve. It is clear that the number of GO-enriched clusters decreased rapidly with the increase of the threshold when the threshold was < 5 (from ∼200 clusters at threshold *n* = 1 down to 85 at the threshold *n* = 5, the blue curve). This threshold-sensitive pattern was recapitulated by the rapid increase in the gradients at each threshold points, especially by the two consecutive rises in the gradients from threshold *n* = 3 to *n* = 4 and from *n* = 4 to *n* = 5 (black curve), transitioning from a threshold-sensitive regime into a threshold-insensitive regime. After the threshold *n* = 5, the blue curve gradually decreased and reached convergence after *n* = 8, accompanied with the almost flat gradient curve (the black curve), which, however, suggests the threshold *n *≥ 8 would be too conservative. Thus, in this study, we used the turning point *n* = 5 as our threshold to trade-off specificity and sensitivity. Furthermore, for module #13, we also considered the sources of the curated interactions. Module #13 consists of 119 proteins mediating 275 interactions and was derived from 109 different publications (with different PubMed IDs, on average ∼2.5 interactions per publication), compared with a total of 16,140 PubMed IDs for 69,113 interactions in the whole network (on average ∼4.28 interactions per publication). The elevated diversity of experimental sources for this module suggests that its network modularity was less likely to be biased toward a particular experimental platform.

### The enrichment of module #13 for ASD gene candidates curated from SFARI

To determine the associations of the network modules with ASD, we first considered the curated genes implicated in ASD and then generalized our comparisons to genes from unbiased genome-wide screens. We first retrieved known autism-associated genes from SFARI Gene (https://gene.sfari.org/autdb/). Among a total of 484 genes in the database (as of February, 2013), 383 were on the protein interaction network. Different versions of these annotated genes were also considered. In addition to using the hypergeometric test to assess the enrichment of the SFARI genes in module #13, we perform a set of permutation tests to ensure that the comparison was not biased by unequal CDS length or GC content. Briefly, we compiled a list of 10,390 genes whose CDS length (the longest RefSeq transcript, Ensembl 72) was similar with the SFARI genes (*P* = 0.24, Wilcoxon rank-sum test). Furthermore, we also compiled a list of 14,041 genes, whose GC content in CDS was similar with the SFARI genes (*P* = 0.58, Wilcoxon rank-sum test). We then considered the intersection between the two gene sets, totaling 7,743 genes (excluding the SFARI genes). Among this gene set with indistinguishable CDS length and GC content, we randomly sampled 383 genes, the same number with the SFARI genes, for 10,000 times (the pseudo-ASD risk genes), and we found that none of the 10,000 random simulations overlapped with module #13 more than the real SFARI gene list, giving an empirical *P* < 1e-5. We also used genes annotated by SynaptomeDB (Pirooznia *et al*, 2012) to control for potential bias from known synaptic genes in this comparison.

### The enrichment of module #13 for ASD gene candidates from genome-wide screens

To determine the enrichment in module #13 for genes implicated in ASD from genome-wide screens, we compared genes in module #13 with 9,782 background genes with indistinguishable CDS length and GC content (*P* > 0.05, Wilcoxon rank-sum test, as described above), and this set of control genes was not overlapping with module #13. For each set of ASD candidate genes (identified by CNV, exome-sequencing studies, etc., Supplementary Table S1), we asked whether or not the module was more enriched for these ASD candidate genes than the matched control gene sets. The exact comparisons can be found in Supplementary Table S1B, where we considered ASD candidate genes affected by *de novo* CNVs, rare CNVs, *de novo* disruptive, missense and silent mutations from large collection of ASD probands. The same categories of mutations identified from non-ASD individuals or the matched unaffected siblings were also analyzed in Supplementary Table S1B. The references for the data sources can be found in Supplementary Table S1A and B, and the complete gene list can be found in Supplementary Dataset S2. Particularly for the *de novo* CNV datasets, we first considered *de novo* CNVs (annotated as “de novo” in their final category) identified from ASD probands from a recent publication (Pinto *et al*, 2014). In addition, *de novo* CNVs from two early studies were also considered (Levy *et al*, 2011; Sanders *et al*, 2011). The union and the intersection of the *de novo* CNV datasets from Pinto *et al* and those from Sanders *et al* or from Levy *et al* were separately tested. Genes with at least one exon affected by these *de novo* CNVs were considered for both ASD and non-ASD subjects. The *de novo* CNVs for non-ASD subjects were collected from a recent publication (Kirov *et al*, 2012). This control CNV dataset was combined with those identified from the unaffected siblings in Sanders *et al* and Levy *et al*. Since these *de novo* CNVs affected thousands of genes in the genome, we also considered a small set of strong candidate genes affected by the ASD-associated high-confidence *de novo* CNVs in this comparison, and these genes were identified from a previous study (Noh *et al*, 2013).

### Collection of genes involved in other psychiatric diseases

We additionally tested enrichment signals in module #13 for genes implicated in schizophrenia, intellectual disability and Alzheimer's diseases. Genes in schizophrenia were obtained from SZGR (http://bioinfo.mc.vanderbilt.edu/SZGR/index.jsp), where 38 core genes and 278 protein-coding genes representing confident loci from previous genome-wide association studies were considered. 613 genes implicated Alzheimer's disease were obtained from AlzGene (http://www.alzgene.org). Genes implicated in intellectual disability were collected in a recent publication (Parikshak *et al*, 2013).

### Whole-genome and exome-sequencing protocols

#### Sample information

Samples were requested from two sources, Autism Speak's Autism Tissue Program (ATP) and NICHD Brain and Tissue Bank (NICHD). Sample information can be found in Supplementary Table S2. Autism diagnosis was confirmed by the clinical practitioners in the brain banks with ADI-R (Autism Diagnosis Interview–Revised). The ATP samples covered the most case DNAs in the ATP's repository (excluding 15q duplication, epilepsy, Angelman syndrome samples or samples from patients’ siblings or samples with no sufficient DNA amount).

#### Sequencing protocol

The genomic DNAs from ATP were extracted from the occipital lobe, Broadmann Area (BA19). We received frozen tissue blocks (postmortem corpus callosum) of six patients from NICHD and extracted genomic DNAs with the use of QIAGEN's DNeasy Blood & Tissue Kit. We used 5 μg DNAs for genome sequencing and 3 μg DNAs for exome sequencing. DNA quality was examined on agarose gel electrophoresis prior to library preparation. Sequencing was on Illumina's HiSeq 2000 platform with 101 × 2 pair-end adaptors. WGS samples were subject to standard Illumina's procedures with variants called by the company's software CASAVA. The called variants were further validated with the Illumina Omni genotyping SNP array with overall concordance rates of ∼99.28%.

The variants were further filtered by removing variants falling in the segmental duplication, simple repeat regions, etc. For exome sequencing, GATK (ver. 2.3.9) was used to call variants by aggregating samples over the targeted intervals designed for exome capture, reaching the average ratio of Ti/Tv 3.18. Agilent SureSelectXT kit (Human All Exon V5+UTRs) was used for exome pull-down in this study. Coverage and Ti/Tv values (transition to transversion rates) for individual samples in WGS and exome sequencing can be found in Supplementary Tables S3 and S4. Variants were annotated using ANNOVAR (Wang *et al*, 2010) based on human genome build hg19.

#### Analysis

Fisher's exact test was used to identify alleles overrepresented in the patient cohort. 1,000 Genome variants’ allele frequencies in all samples or only in Europeans were referenced in the analysis. The *P*-values for variants in this module were further corrected with the Benjamini–Hochberg procedure. The functional consequences of the identified variants were tested by MutationTaster (Schwarz *et al*, 2010), where the automatic annotations based on the 1,000 Genome frequencies were overridden by the prediction from the original Bayesian classifier. Phenotypic analysis of the identified genes was based on the component of Human-Mouse: Disease Connection in Mouse Genome Informatics (http://www.informatics.jax.org/humanDisease.shtml).

#### Validation using dbGAP data

We were approved to use one exome-sequencing dataset in dbGAP, which sequenced a larger patient population in previous study (Liu *et al*, 2013). Half of the samples were sequenced in Broad Institute (by the Illumina platform) and the other half in Baylor College Medicine (BCM, by the SOLiD platform). Due to incomplete data deposited in dbGAP for those sequenced on the Illumina platform, we were only able to study the subjects sequenced by BCM, including 505 unrelated patients and 491 controls, all with European ethnicity. Variants showing the most significant deviation in their allele frequencies from the control subjects were identified with a regression analysis. We regressed case/control frequencies reciprocally, followed by a residue analysis that identified outliers exceeding the upper 5% bound of the residue distribution modeled by a *t*-distribution.

### Expression analyses of the module across brain sections

Expression data were from Allen Brain Atlas (Hawrylycz *et al*, 2012), where gene expression was measured with microarrays across hundreds of anatomical sections in two representative individuals (9,861 and 10,021). The microarray data had been normalized and postprocessed by Allele Brain Atlas, and we considered 295 brain sections that were measured in both individuals (by matching the brain section identifiers). Expression of a given gene in a given tissue was then averaged over the two individuals to reduce the potential individual-specific fluctuations. In addition, signals of multiple probes mapped onto the same transcripts were also averaged in this analysis. The expression profiles were then normalized across sections followed by a hierarchical clustering, which allowed identifying gene groups sharing similar spatial expression patterns. In each brain section, the absolute expression of genes in Group 1 and 2 was also compared against the transcriptomic background in the corresponding section. Tissue specificity index was computed for individual genes across the 295 brain sections using the following formula defined in a previous study (Yanai *et al*, 2005), 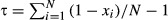, where τ is the tissue specificity index of a given gene, *N* is the total number of different brain sections, and *x*_*i*_ is the gene's expression in a section, *i*. Expression breadth of a given gene was determined by the number of brain sections where the gene is active, and we varied the threshold to define gene activity based on the distribution of the absolute gene expression across the transcriptomes in the 295 brain sections (Supplementary Fig S9). The thresholds chosen in our comparison were 15, 25 and 50% of the data points across all genes, and expression values below these cutoffs were deemed to be inactive.

Genes in this module were further mapped onto the mouse genome by identifying their one-to-one mouse orthologs based on Ensembl Gene (as of August, 2013). Mouse expression data for neurons, oligodendrocytes and astrocytes were retrieved from a previous study (Cahoy *et al*, 2008). Chi-square test was used to determine the imbalanced distribution of genes in Group 1 and 2 in the neuron and glial cluster, respectively (Fig4B). Mouse expression data in the oligodendrocyte precursor cells (OPCs), the mature oligodendrocytes (OLs) and the MRF conditional knockouts were retrieved from a previous study (Emery *et al*, 2009). We mapped the probes onto mouse gene symbols and averaged signals from multiple probes mapped onto the same genes. Expression across multiple biological replicates under the same condition was averaged.

### Immunohistochemistry analysis of the postmortem corpus callosum

Immunohistochemistry analysis was performed on the corpus callosum from a patient (#5308) and a control subject (#4727). Anti-LRP2 antibody was purchased from Abcam (cat#: ab76969, Abcam, Cambridge, MA). Immunohistochemistry labeling for LRP2 was carried out using the DAKO EnVision system (cat#: K4065, DAKO, Carpinteria, CA) at 1:100; slides were developed using the Dako Envision method as the manual suggested. Heat-induced antigen retrieval was performed with Decloaking Chamber (Biocare Medical, Concord, CA) in citrate buffer (pH 6.0). Human kidney carcinoma tissue and normal human ovary were used as positive and negative controls given the presence and absence of LRP2 (from literature) in these two tissues, respectively. In addition, IgG was also used as a control for the specificity of anti-LRP2. Cell types in the corpus callosum were independently identified and verified by a neuro-pathologist at Stanford.

### RNA-sequencing protocols

#### Sample information

Postmortem tissues of corpus callosum from 12 individuals were subject to RNA-sequencing in this study. Frozen tissue blocks were all provided by NICHD Brain and Tissue Bank. The samples were all European males, and case–control pairs were matched in terms of their age, sex and PMI (depends on tissue availability). All the control subjects have been optimized for comparisons and were selected by the brain bank to match the cases. The case–control pairs are listed in Supplementary Table S5. We also biologically replicated our experiments on 6 out of 12 individuals by sectioning different areas of the tissue blocks. In addition to the corpus callosum, we also sequenced three brain sections (NICHD) for a control subject #5407 (Supplementary Table S5), including Brodmann areas 9, 40, and also the amygdala.

#### Sequencing protocols

Total RNA was extracted from flash-frozen tissue samples using Trizol reagent. Then, the total RNA was treated with RNase-Free DNase (Qiagen) followed by purification with RNeasy MinElute Cleanup Kit (Qiagen) following the manufacturer's instructions. 2 μg of total RNA each sample was subject to RNA-Seq library preparation with ScriptSeq™ Complete Gold Kit from Epicentre (Cat. #SCL24EP, Madison, WI) following the manufacturer's instructions. In brief, ribosomal RNA was depleted from total RNA using Ribo-Zero magnetic beads, and then, the ribosomal RNA-depleted RNA was purified and fragmented. Random primer tailed with Illumina adaptor was used to perform reverse transcription to get cDNA library. Adaptor sequence was added to the other end of cDNA library with a Terminal-Tagging step. cDNA library was amplified with Illumina primers provided with this kit. The product was size selected (350–500 bp) from 2% agarose E-gels (Invitrogen) and sequenced in 1 lane per sample on Illumina's HiSeq 2000 platform.

#### Analysis

The sequenced 101 × 2 pair-end fragments were mapped against the human RefSeq transcriptome using TopHat v2.0.8 (http://tophat.cbcb.umd.edu). Quantitation of expression levels was computed with CuffLinks v2.0.2 (http://cufflinks.cbcb.umd.edu). We excluded genes with low expression in both cases and controls (FPKM < 1) to avoid numerical fluctuations by small numbers and retained ∼12,000 highly expressed genes in this study (with “OK” status from Cufflinks calculation), which were likely more relevant to the physiology of this particular tissue type. We also retrieved the medical and neuropathology records of these patients and found that three patients had no documented medication history related to ASD. The other three patients took medications to correct their ASD-related behaviors; however, the potential drug targets (determined by microarray study upon drug exposure or literature curation, data not shown) were not present in our module. Therefore, medication cannot fully explain the dys-regulated genes in our module.

### Human subjects

This study was exempt from Stanford IRB review since only postmortem brain tissues from de-identified and deceased individuals were examined in this study. Brain tissues/DNA extracts were obtained from ATP and NICHD, where informed consent was obtained from all subjects. The experiments conformed to the principles set out in the WMA Declaration of Helsinki and the Department of Health and Human Services Belmont Report.

### Data availability

RNA-sequencing data are deposited in GEO with the accession identifiers: GSE62098 and GSE63513. DNA-sequencing data are deposited in SRA with the accession identifiers SRP050187.

## Supporting Information

Supplementary Dataset S4

Review Process File
