# Supplementary figures and images for: Integrated systems analysis reveals a molecular network underlying autism spectrum disorders
Source: Mol Syst Biol. 2014 Dec 30;10(12):774. doi: 10.15252/msb.20145487 (PMC4300495; doi:10.15252/msb.20145487)

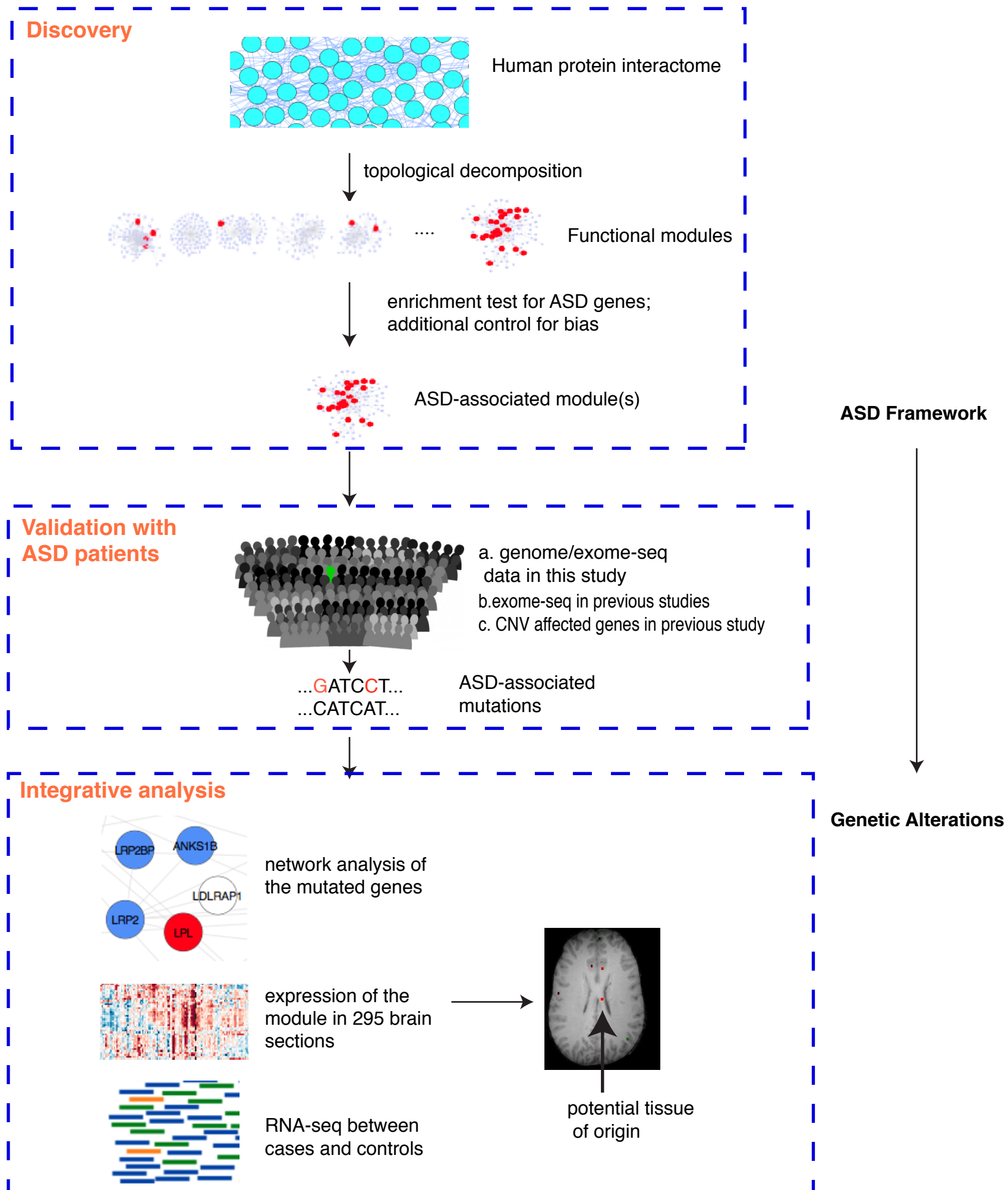

**Fig. S1**

Supplement: Supplementary file 1 [file msb0010-0774-sd1.pdf]

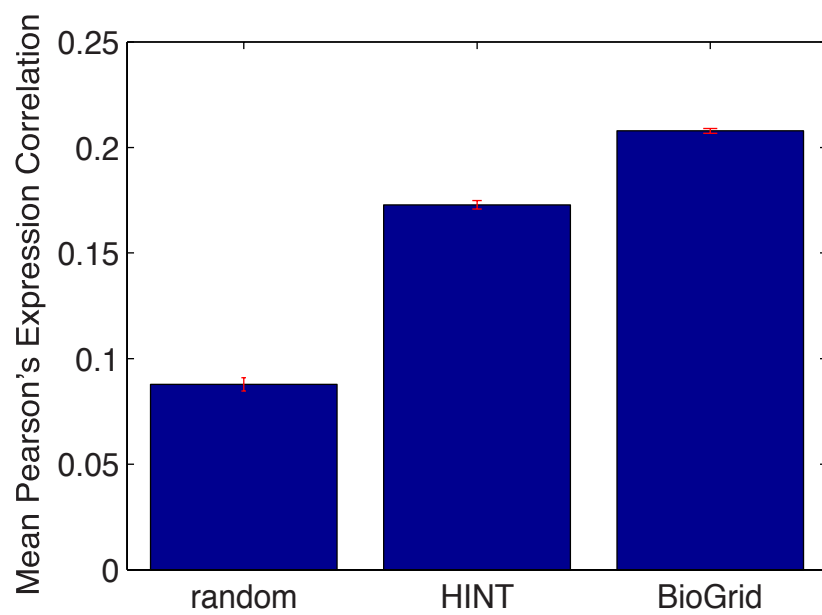

Supplement: Supplementary file 2 [file msb0010-0774-sd2.pdf]

**A**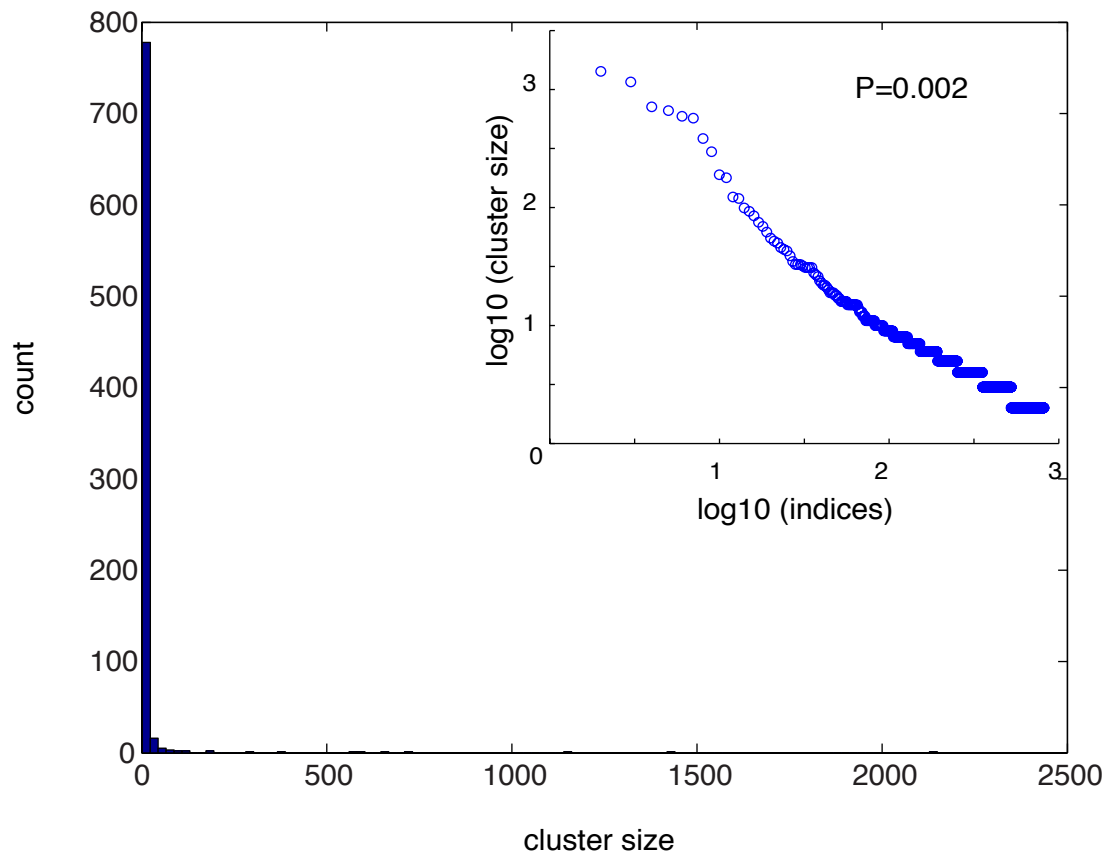**B**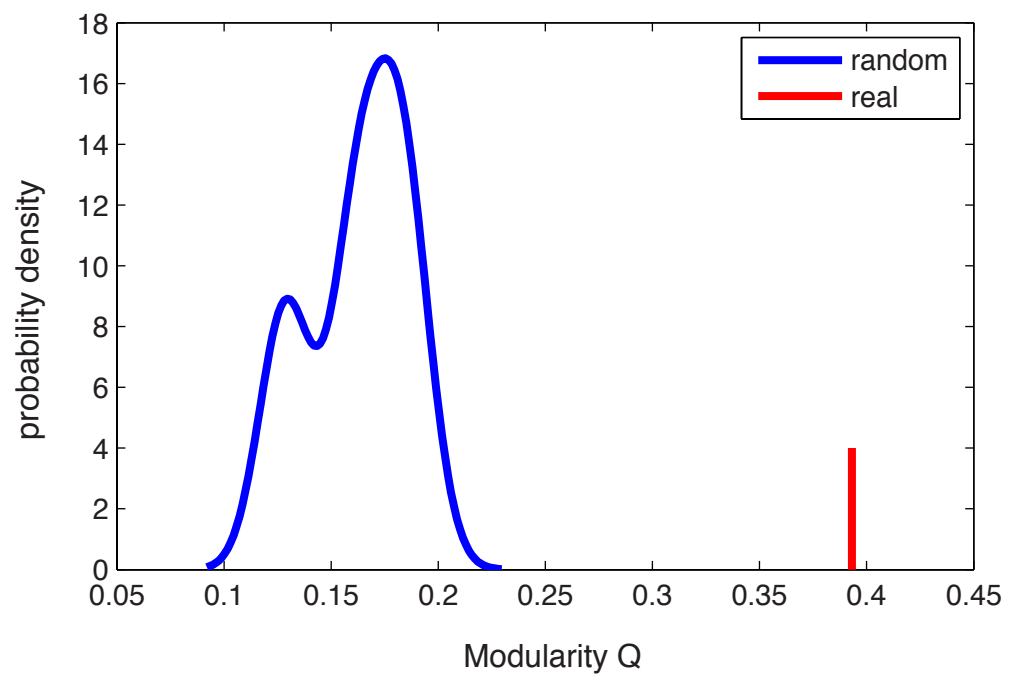

Supplement: Supplementary file 3 [file msb0010-0774-sd3.pdf]

**A**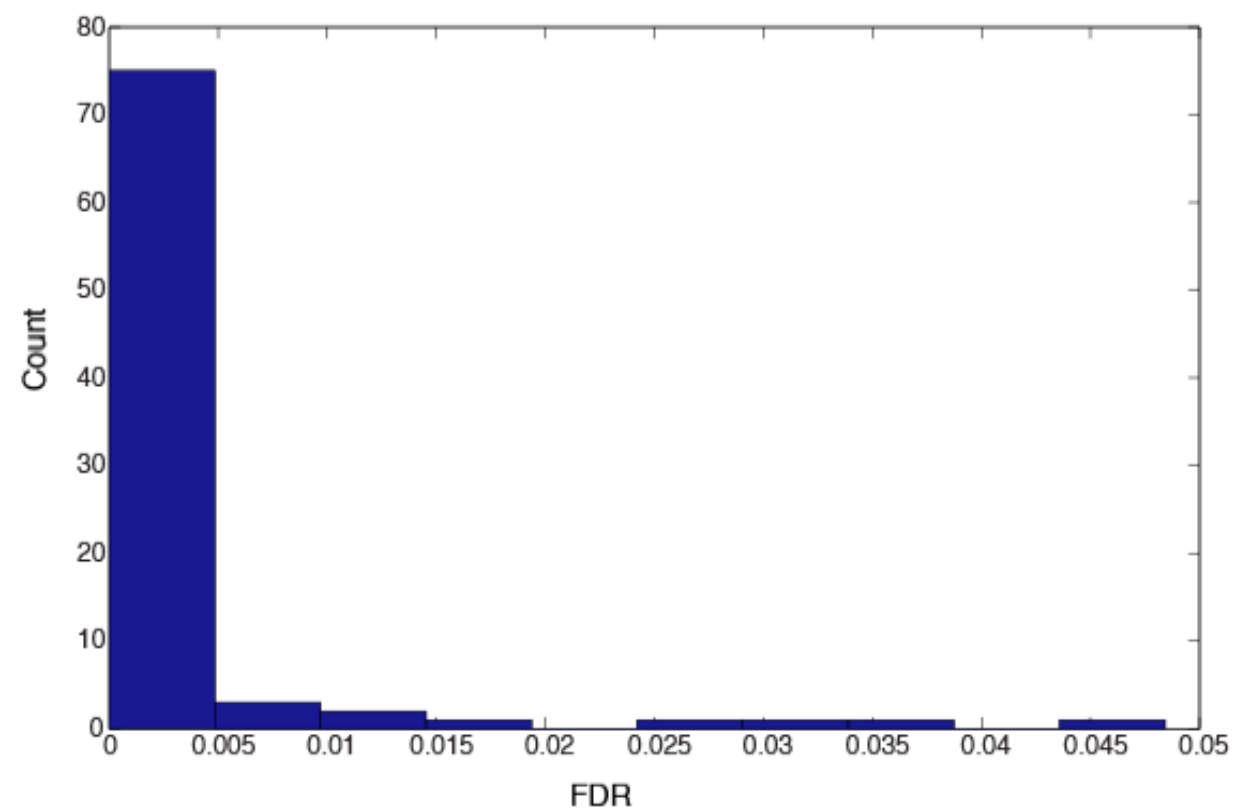**B**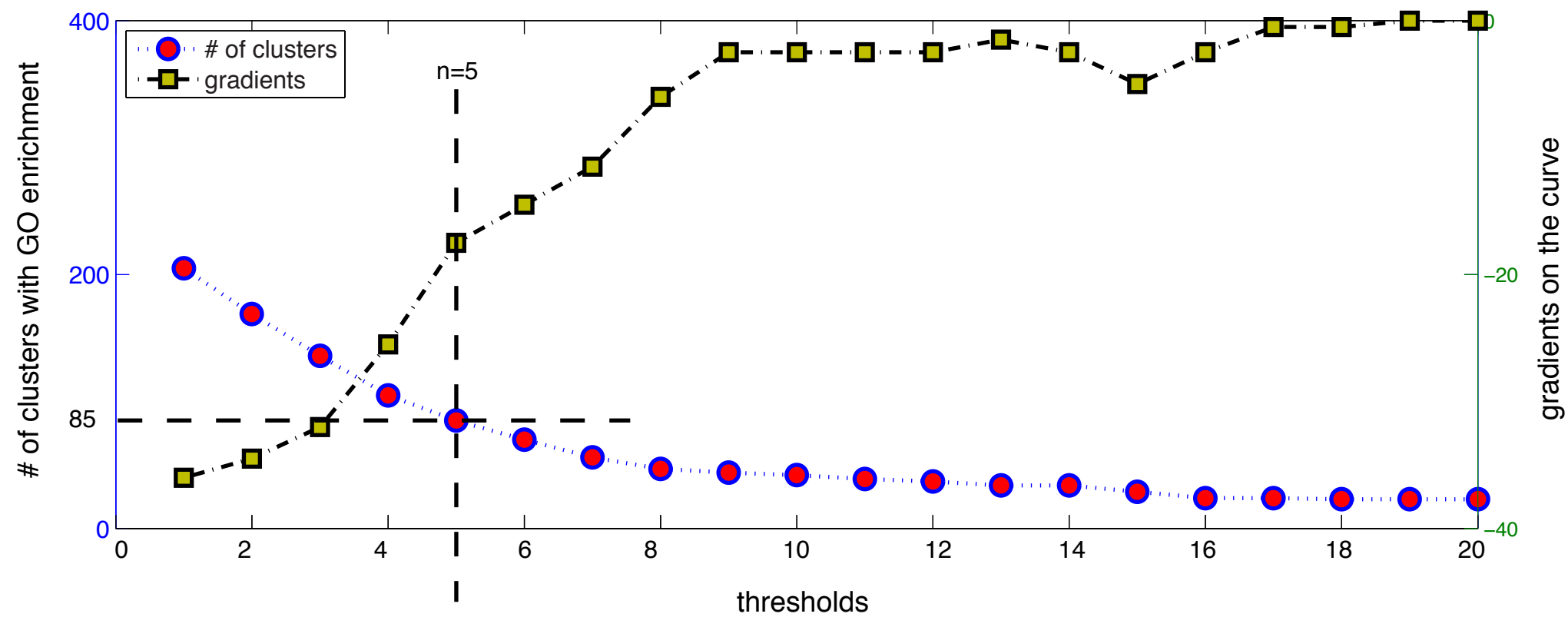

Supplement: Supplementary file 4 [file msb0010-0774-sd4.pdf]

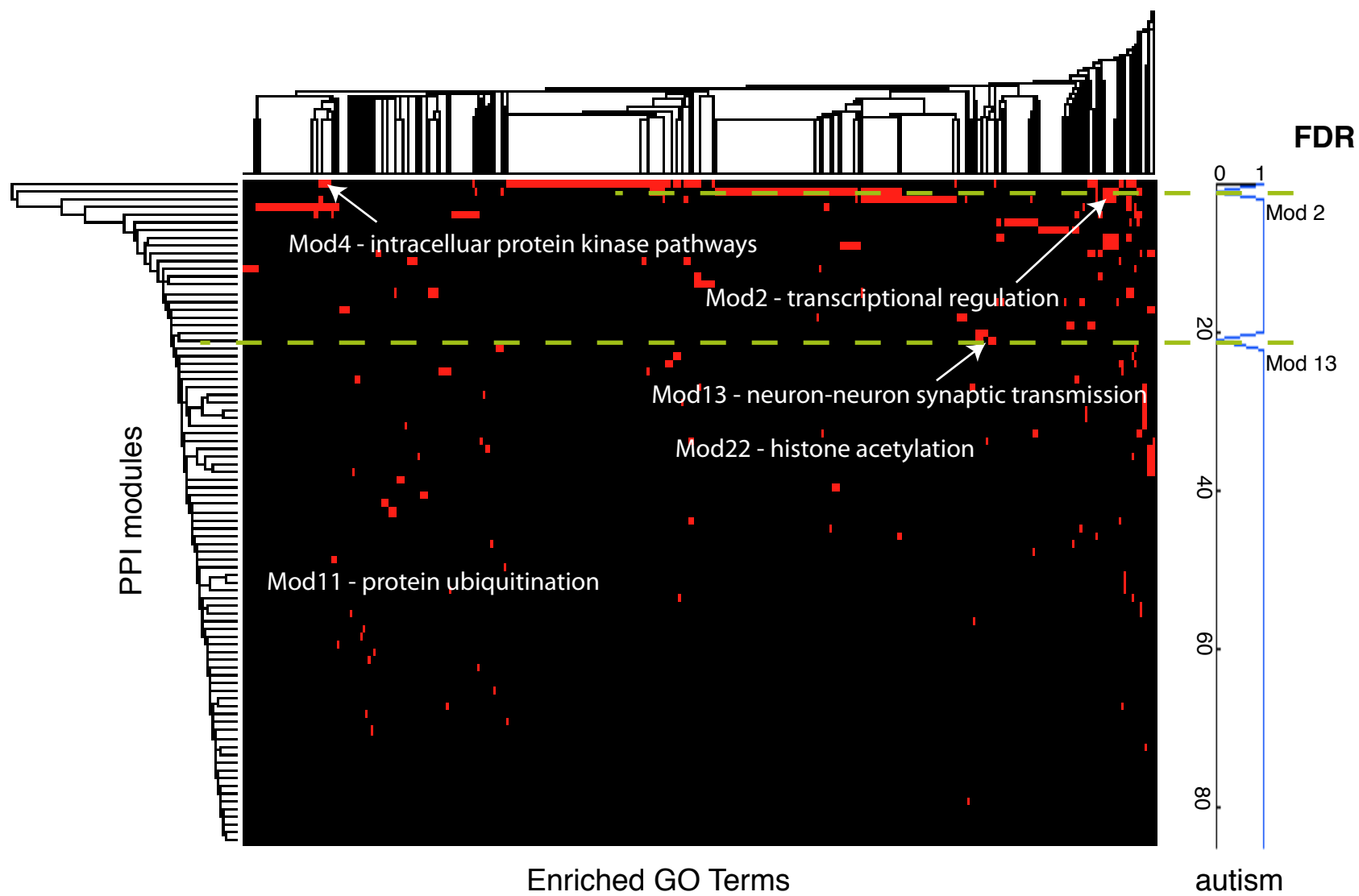

**Supplementary Fig. S5**

Supplement: Supplementary file 5 [file msb0010-0774-sd5.pdf]

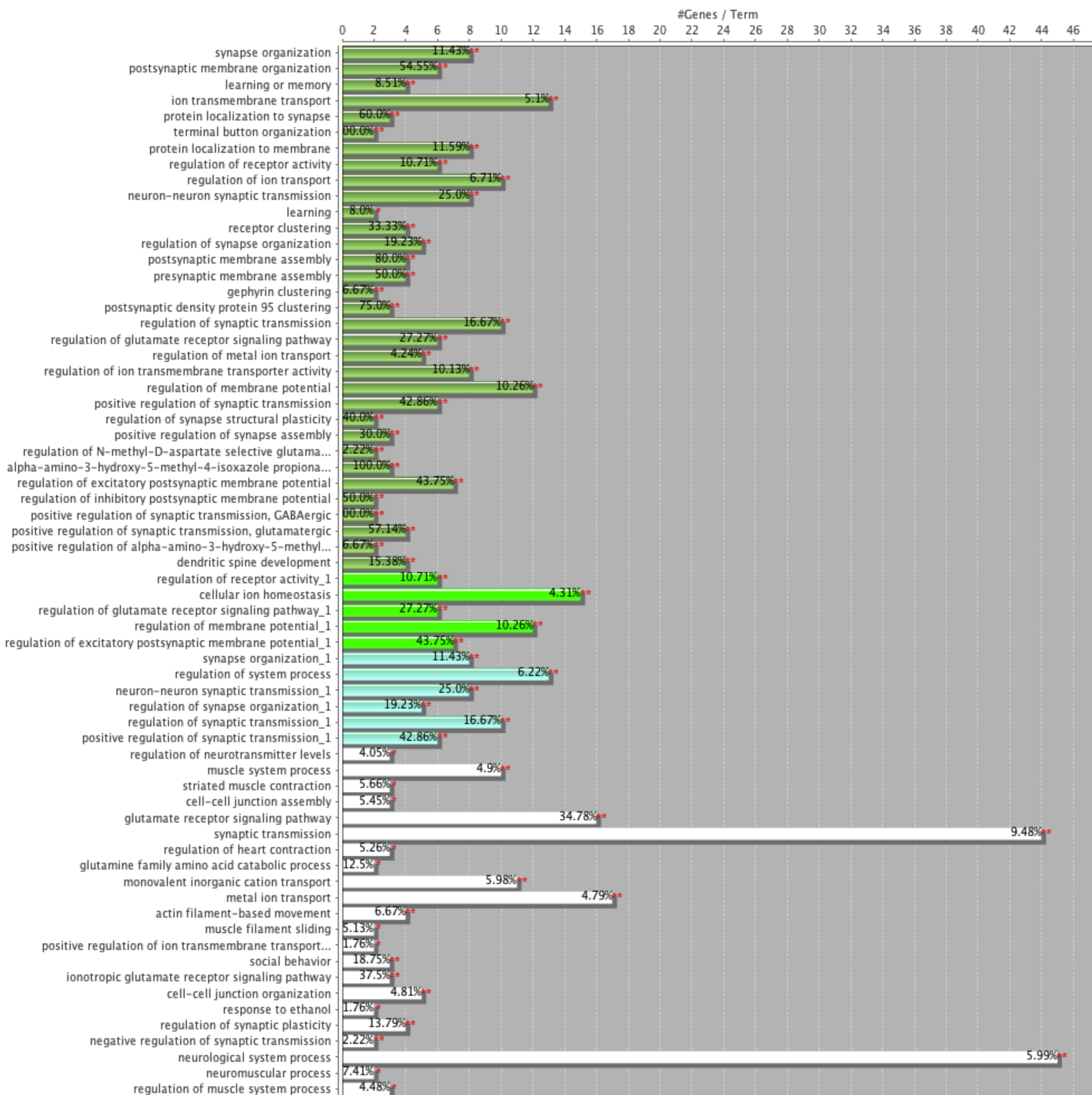

Supplementary Fig. S6

Supplement: Supplementary file 6 [file msb0010-0774-sd6.pdf]

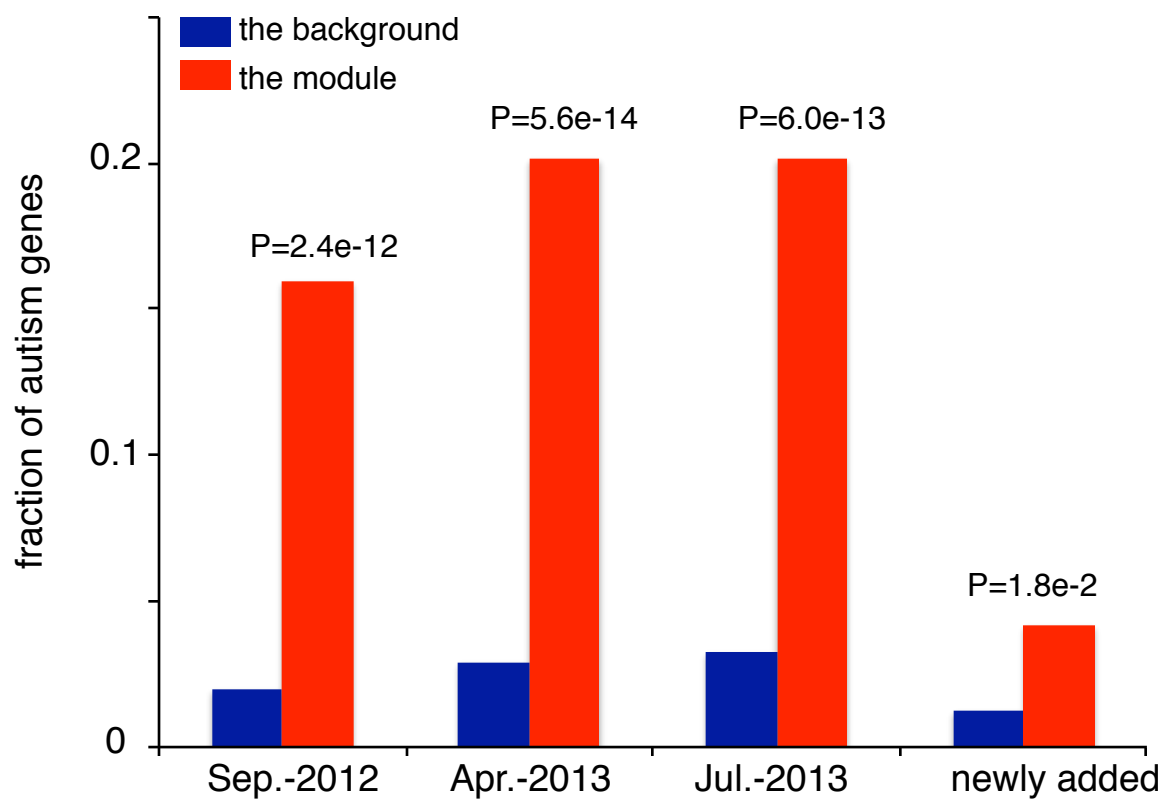

Supplement: Supplementary file 7 [file msb0010-0774-sd7.pdf]

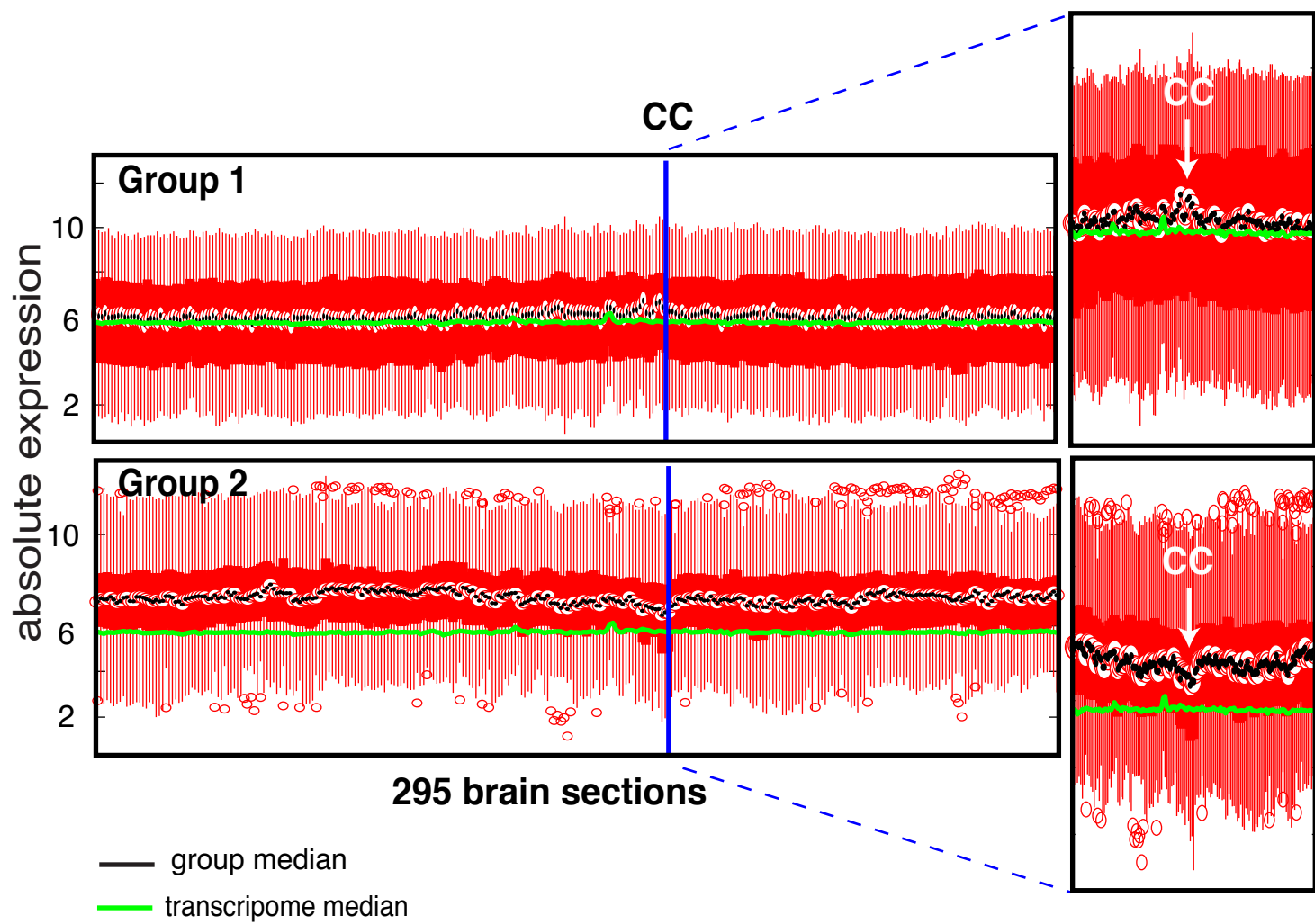

Supplement: Supplementary file 8 [file msb0010-0774-sd8.pdf]

**A**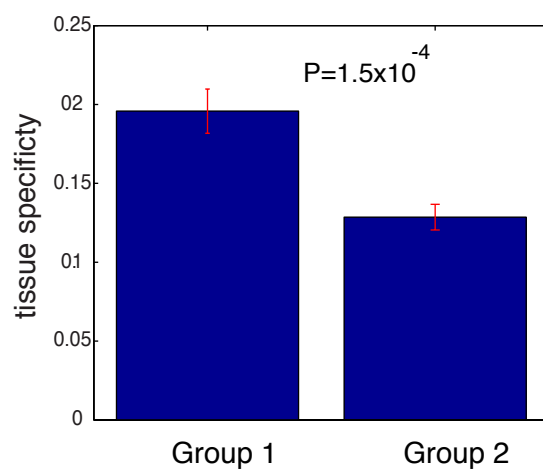**B**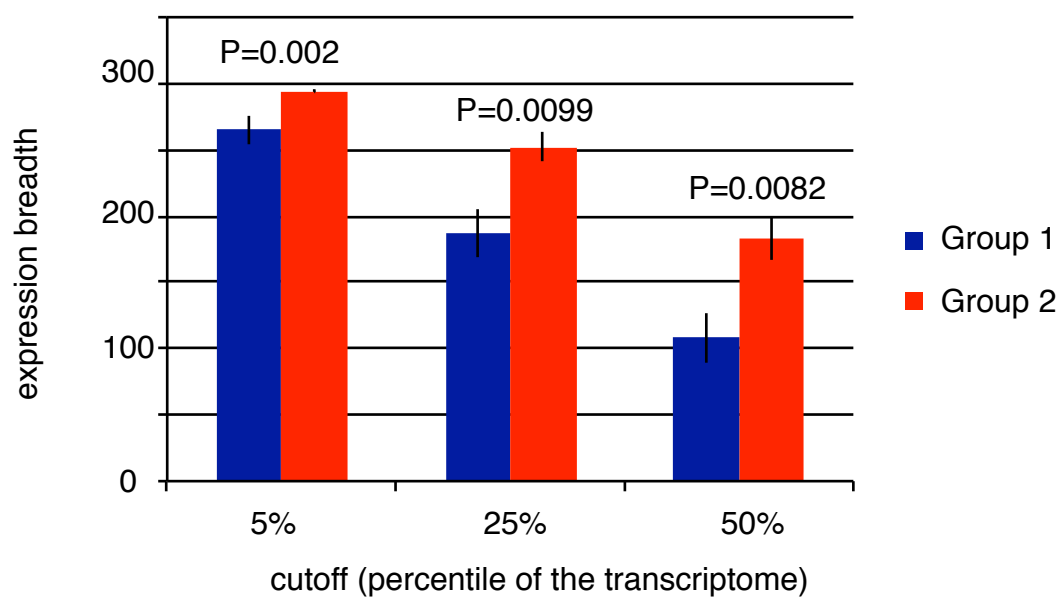

Supplement: Supplementary file 9 [file msb0010-0774-sd9.pdf]

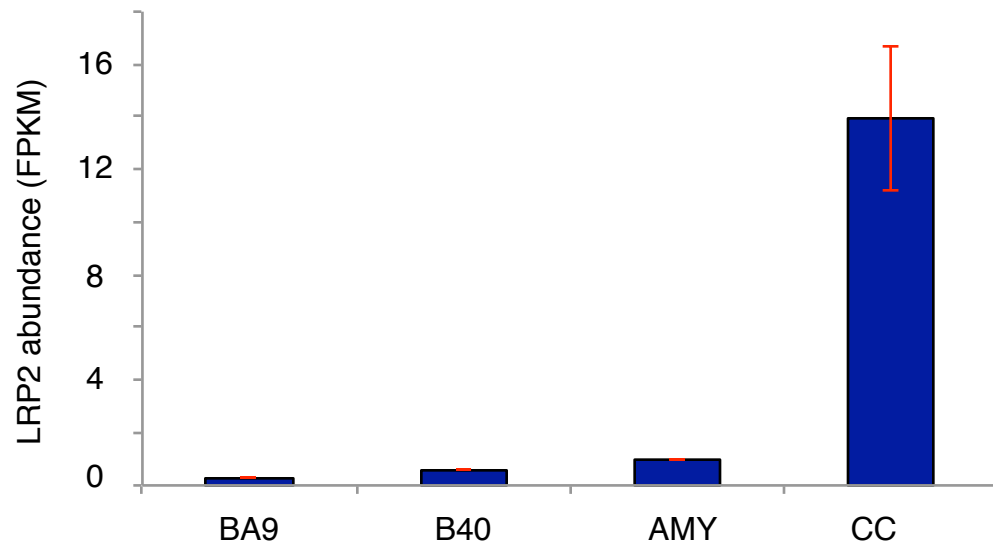

Supplement: Supplementary file 10 [file msb0010-0774-sd10.pdf]

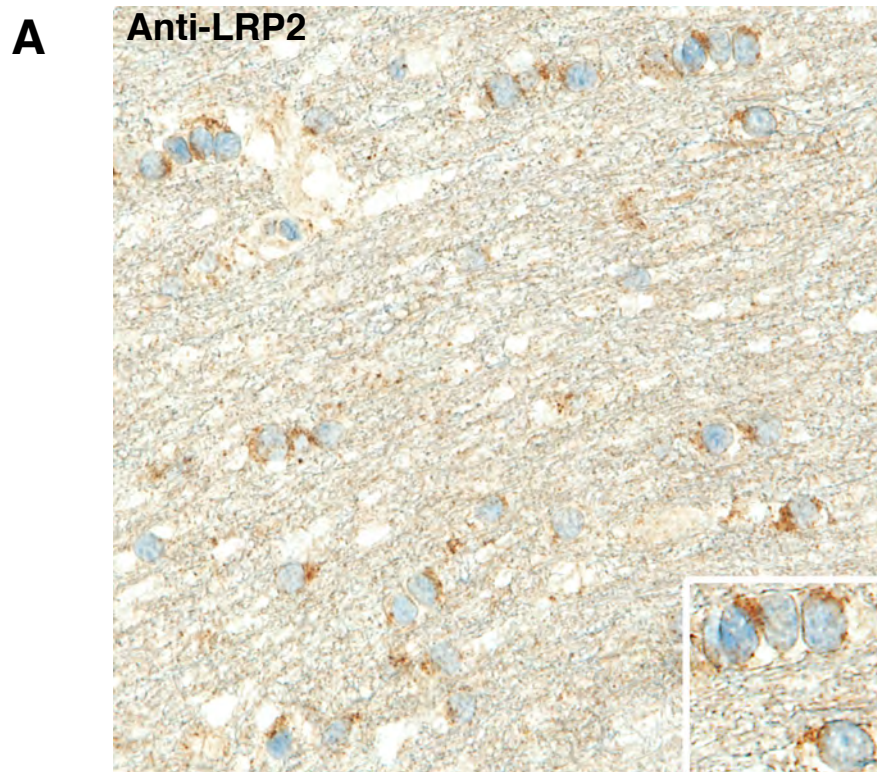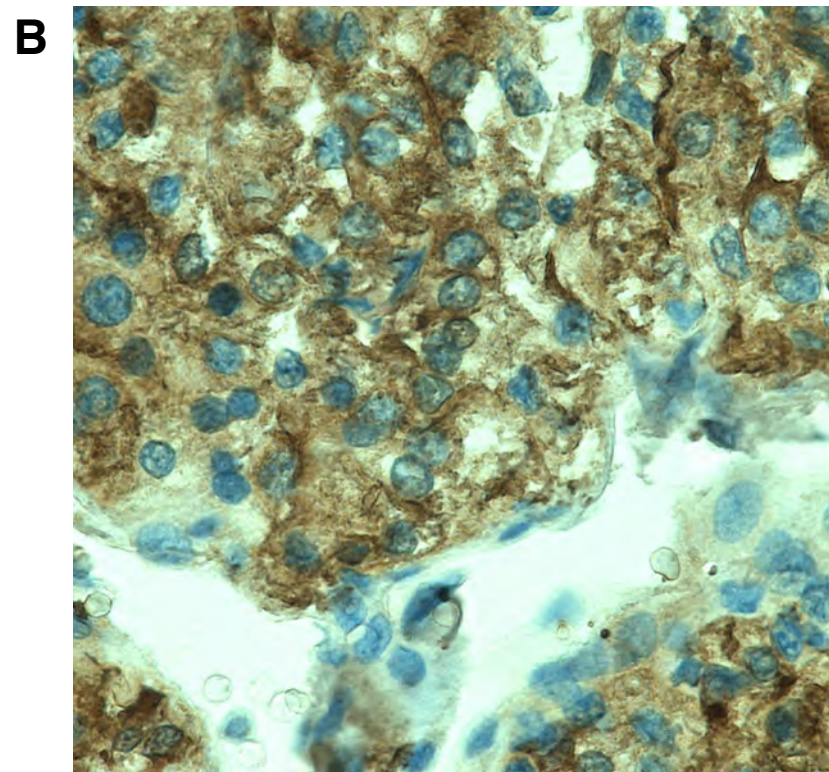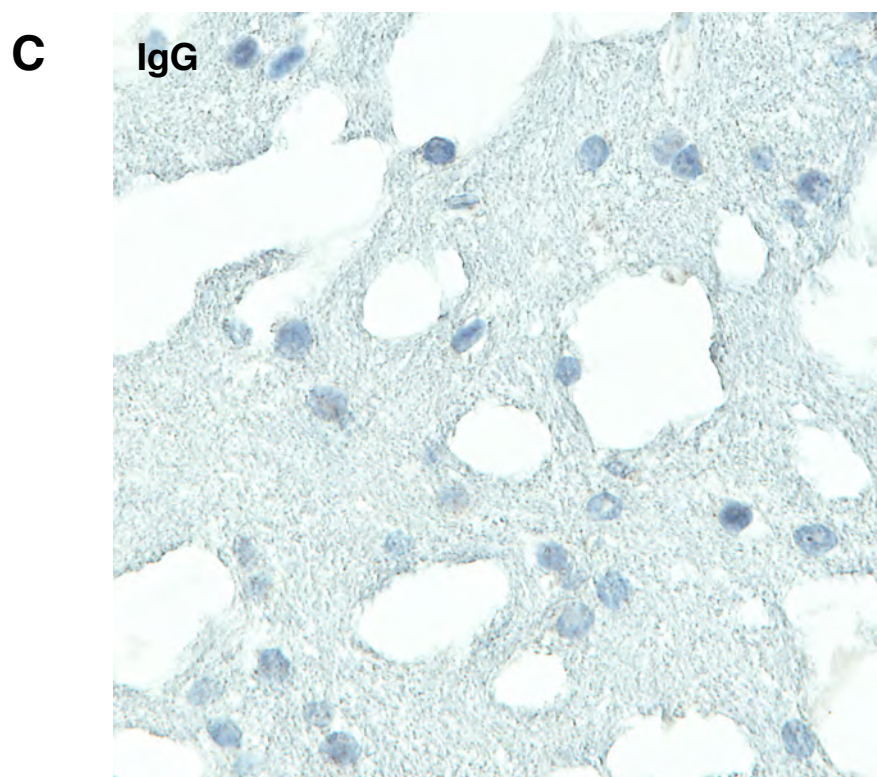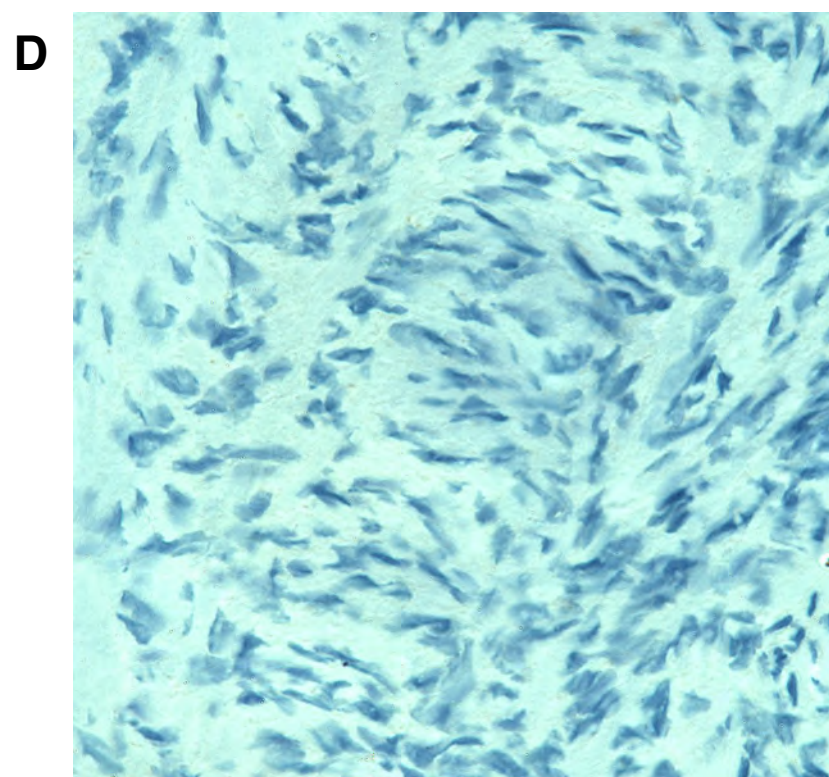

**Supplementary Fig. S11**

Supplement: Supplementary file 11 [file msb0010-0774-sd11.pdf]

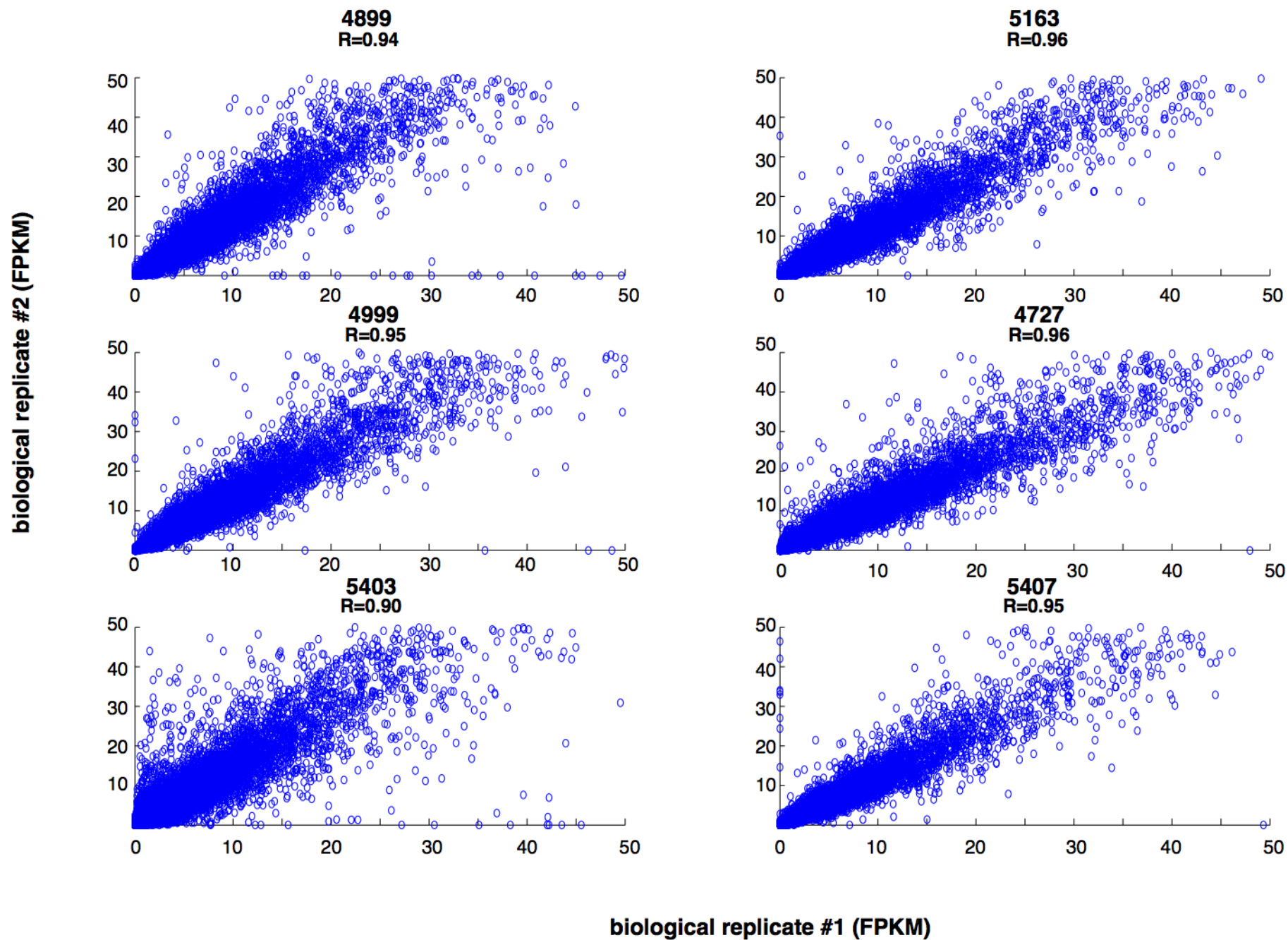

Supplementary Fig. S12

Supplement: Supplementary file 12 [file msb0010-0774-sd12.pdf]

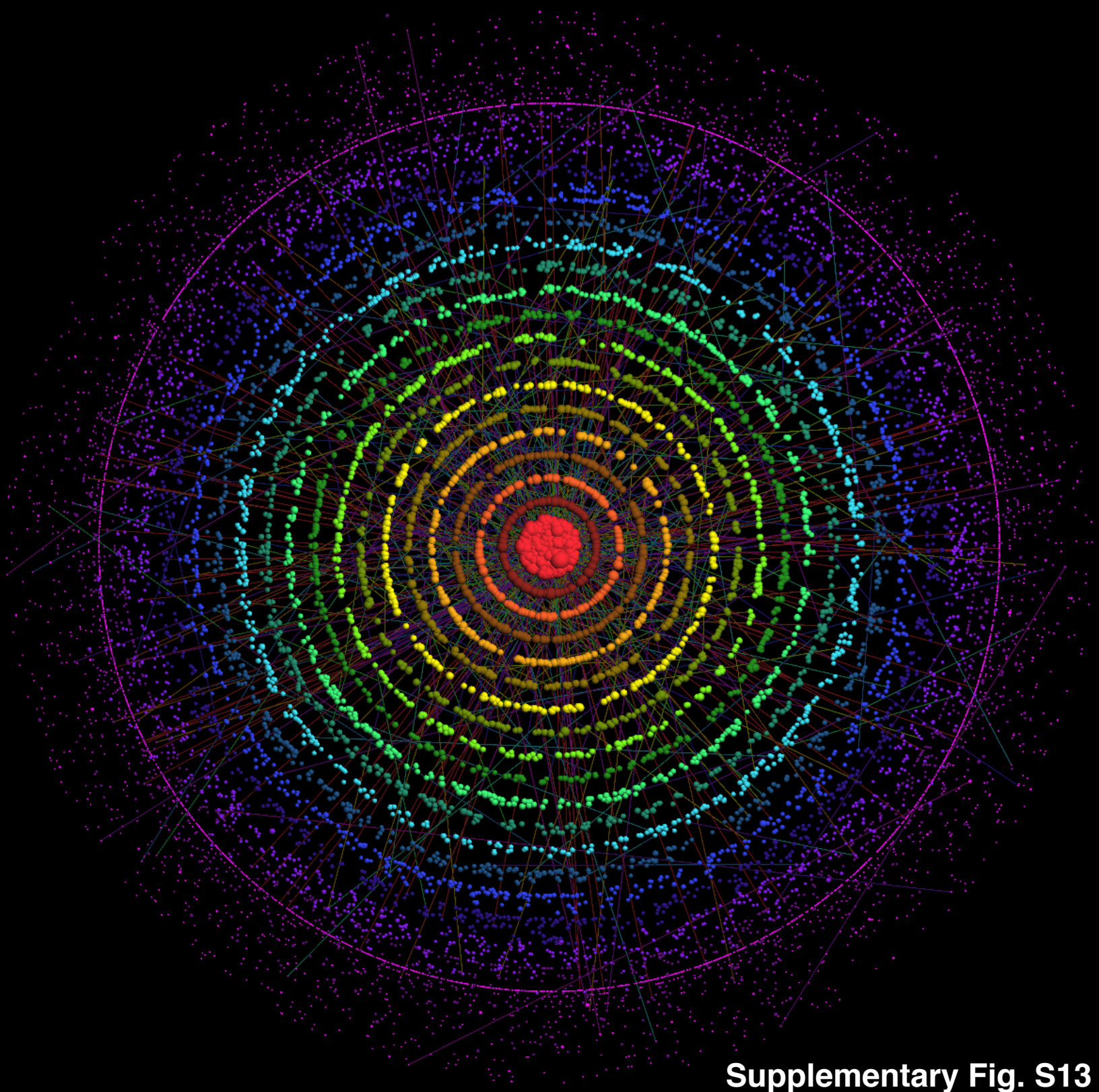

**Supplementary Fig. S13**

Supplement: Supplementary file 13 [file msb0010-0774-sd13.pdf]

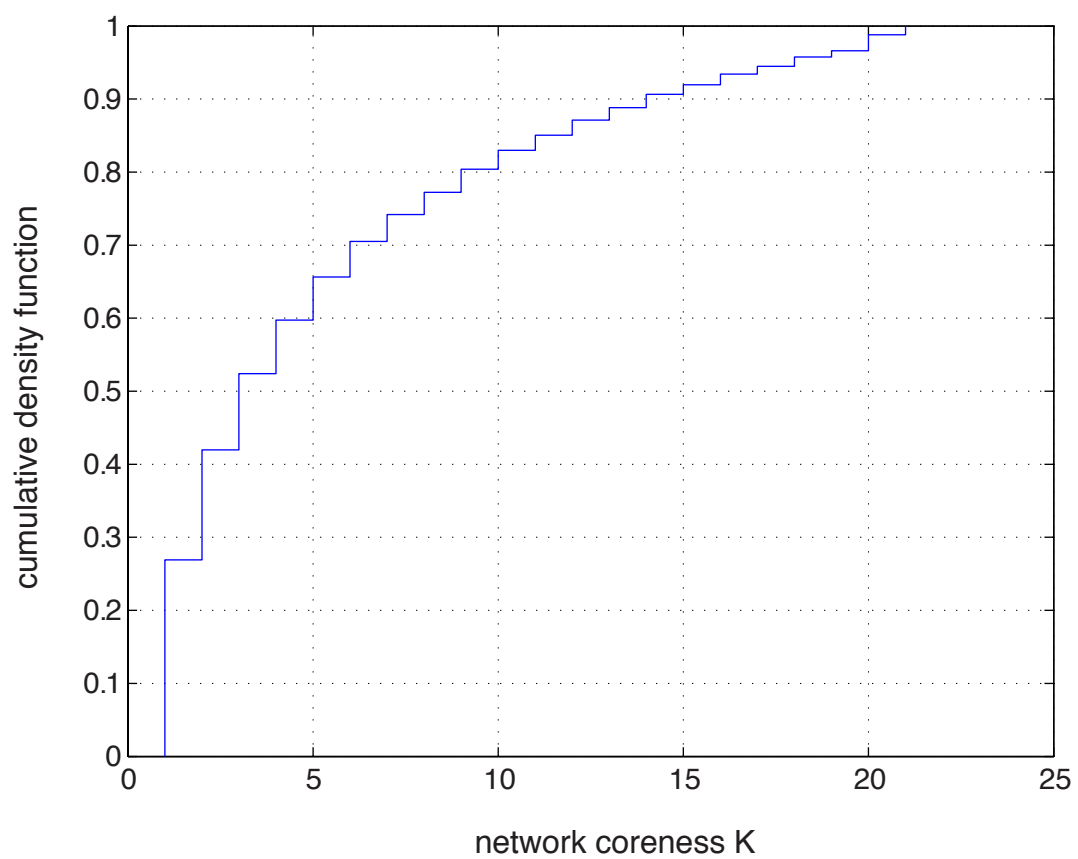

Supplement: Supplementary file 14 [file msb0010-0774-sd14.pdf]
