# Supplementary material for: Integrated systems analysis reveals a molecular network underlying autism spectrum disorders
Source: Mol Syst Biol. 2014 Dec 30;10(12):774. doi: 10.15252/msb.20145487 (PMC4300495; doi:10.15252/msb.20145487)
Supplement: Supplementary file 15 [file msb0010-0774-sd15.pdf]

Table S1. Validation for the enrichment of ASD genes in this module

<table>
<tr><th></th><th>Validation method</th><th>Dataset</th><th>Conclusion</th></tr>
<tr><td rowspan="3">SFARI-based validation</td><td>Comparing with the human synaptome background</td><td>synaptomeDB
SFARI</td><td>Our module is more enriched for ASD genes than that of genes in the synaptome background (P=3.28e-8, Fisher’s exact test)</td></tr>
<tr><td>Enrichment of the non-synaptic genes in module</td><td>synaptomeDB
SFARI</td><td>Genes in our module not annotated as synaptic genes also showed enrichment for ASD genes (P=1.64e-4, hypergeometric test)</td></tr>
<tr><td>High-confidence loci (with syndromic mutations) and different SFARI versions</td><td>SFARI (gene-scoring module, category S)</td><td>Significant enrichment (P≤3.85e-06, Fisher’s exact)</td></tr>
<tr><td rowspan="8">Independent validation</td><td>Enrichment of genes affected by de novo ASD disruptive mutations</td><td>Sanders, et al; O'Roak, et al.; Neale, et al., 2012, Nature</td><td>Significant enrichment (P=0.029, Fisher’s exact)</td></tr>
<tr><td>Enrichment of genes affected by de novo CNVs in ASD probands</td><td>Pinto, et al. 2014, AJHG
Sanders et al. and Levy et al. 2011, Neuron</td><td>Significant enrichment (P=0.01, Fisher’s exact)</td></tr>
<tr><td>Enrichment of the high-confidence ASD candidate genes with recurrent de novo disruptive mutations</td><td>Willsey, A.J. et al. Cell, 2014</td><td>Significant enrichment (P=3.8e-3, Fisher’s exact)</td></tr>
<tr><td>Enrichment of genes affected by de novo CNVs associated with ASD patients</td><td>Noh, H.J. et al. PLoS Genet., 2013</td><td>Significant enrichment (P=3.1105e-13, Fisher’s exact)</td></tr>
<tr><td>Enrichment of genes affected by rare ASD CNVs</td><td>Pinto, D. et al., Nature 2010</td><td>Significant enrichment (P=0.0475, Fisher’s exact)</td></tr>
<tr><td>Enrichment of rare nonsynonymous mutations</td><td>genome/exome-sequencing in this study</td><td>Significant enrichment (P=1.2e-3, hypergeometric test)</td></tr>
<tr><td>Replication on exome-seq data for >500 patients</td><td>Liu, L. et al. PLoS Genet.</td><td>Significant overlap with the candidate loci identified in our sequencing study</td></tr>
<tr><td colspan="3">1. SynaptomeDB (http://psychiatry.igm.jhmi.edu/SynaptomeDB/)
2. For the comparisons with previously published sequencing dataset, the control gene set includes a set of 9782 genes with indistinguishable CDS length and GC content from the genes in the module.
3. For comparisons involving ASD probands, the same comparisons on unaffected siblisng were also performed.</td></tr>
</table>

Table S1B. Enrichment test for genes with different types of mutations in ASD probands and unaffected siblings					
Test for ASD candidate genes					
**CNV test**	**module**	**matched control**	**Fold-change**	**P (Fisher's exact)**	**References (PMID)**
de novo CNV (ASD-union, 2753 genes)	19.33%	11.27%	1.7152	0.0124	24768552, 21658581, 21658582
de novo CNV (ASD-intersection, 545 genes)	5.04%	2.07%	2.435	0.0393	24768552, 21658581, 21658582
HC CNV in ASD (203 genes)	14.29%	1.20%	11.91	3.11E-13	23754953
de novo CNV (nonASD, 557 genes)	1.68%	2.65%	0.634	0.7725	21658581, 21658582, 22083728
rare ASD CNV in ASD (407 genes)	5.04%	2.17%	2.3226	0.0475	20531469
**de novo SNVs**					22495309, 22495306, 22495311
de novo disruptive in proband (67 genes)	2.52%	0.54%	4.67	0.03	
de novo disruptive in siblings (8 genes)	0.00%	0.06%	0	1	
de novo missense in proband (366 genes)	5.04%	2.81%	1.79	0.1543	
de novo missense in siblings (109 genes)	0.84%	0.73%	1.15	0.5826	
de novo silent in proband (148 genes)	0.84%	1%	0.84	1	
de novo silent in siblings (52 genes)	0	0.40%	0	1	
**SFARI genes (484 genes)**	21%	3.40%	6.18	5.84E-13	https://gene.sfari.org
HC SFARI genes (category S)	5.04%	0.31%	16.26	3.85E-06	

control gene set is matched with CDS length and GC content
