# Supplementary material for: Integrated systems analysis reveals a molecular network underlying autism spectrum disorders
Source: Mol Syst Biol. 2014 Dec 30;10(12):774. doi: 10.15252/msb.20145487 (PMC4300495; doi:10.15252/msb.20145487)
Supplement: Supplementary file 16 [file msb0010-0774-sd16.pdf]

Table S2. Sample Information for DNA-sequencing

ID	AN#	AGE	SEX	PMI	ETHNICITY	DIAGNOSIS	SEQ TYPE	SOURCE
133332	AN03217	19	M	18.58	European	NA	WGS	ATP
133350	AN06420	39	M	13.95	European	ADI-R	WGS	ATP
133334	AN10833	22	M	21.47	European	NA	WGS	ATP
111305	AN11989	30	M	16.06	European	ADI-R	WGS	ATP
133337	AN17450	0	M	5	European	NA	WGS	ATP
111291	AN00764	20	M	23.7	European	Autism - confirmed by ADI-R	WGS	ATP
111297	AN19511	8	M	22.2	European	Autism - confirmed by ADI-R	WGS	ATP
111302	AN03345	2	M	4	European	Autism - confirmed by ADI-R	WGS	ATP
133331	AN07444	17	M	30.75	European	NA	WGS	ATP
111301	AN09730	22	M	25	European	Autism - confirmed by ADI-R	WGS	ATP
111289	AN16641	9	M	27	European	ADI-R	EXOME	ATP
111290	AN00493	27	M	8.3	European	ADI-R	EXOME	ATP
111292	AN08792	30	M	20.3	European	ADI-R	EXOME	ATP
111296	AN08873	5	M	25.5	European	ADI-R	EXOME	ATP
111299	AN01570	18	F	6.75	European	ADI-R	EXOME	ATP
111304	AN12457	29	F	17.83	European	ADI-R	EXOME	ATP
111310	AN08166	28	M	43.25	European	ADI-R	EXOME	ATP
111313	AN17678	11	M	-	European	ADI-R	EXOME	ATP
111316	AN09714	60	M	26.5	European	Autism - confirmed by ADI-R	EXOME	ATP
111317	AN17254	51	M	22.16	European	ADI-R	EXOME	ATP
133328	HSB-4640	8	M	13.8	European	Autism-supported by records	EXOME	ATP
133341	AN16115	11	F	12.88	European	ADI-R	EXOME	ATP
133344	AN08043	52	F	39.15	European	ADI-R	EXOME	ATP
133346	AN02456	4	F	17.02	European	NA	EXOME	ATP
5403	#	16	M	35	European	ADI-R	EXOME	NICHD
5144	#	7	M	3	European	ADI-R	EXOME	NICHD
5308	#	4	M	21	European	ADI-R	EXOME	NICHD
5302	#	16	M	20	European	ADI-R	EXOME	NICHD
4899	#	14	M	9	European	ADI-R	EXOME	NICHD
4999	#	20	M	14	European	ADI-R	EXOME	NICHD

Notes - ADI-R: autism diagnostic interview, revised; NA: control subjects with no diagnosed autism; WGS: whole-genome sequencing; Exome: exome sequencing; ATP: Autism Tissue Program; NICHD: NICHD Brain and Tissue Bank; PMI: postmortem interval.
