# Supplementary material for: Integrated systems analysis reveals a molecular network underlying autism spectrum disorders
Source: Mol Syst Biol. 2014 Dec 30;10(12):774. doi: 10.15252/msb.20145487 (PMC4300495; doi:10.15252/msb.20145487)
Supplement: Supplementary file 17 [file msb0010-0774-sd17.pdf]

Table S3. Information for whole-genome sequencing

ID	133350	111297	133334	133337	111302	111301	133331	111291	133332	111305
Ti/Tv	2.027	2.025	2.030	2.030	2.032	2.026	2.031	2.026	2.13	2.10
Cvg*	38.3	38.7	38.6	34.7	38.4	35.9	37.2	40.4	36.2	41.9
Array*	99.28%	99.28%	99.27%	99.25%	99.26%	99.26%	99.28%	99.28%	-	-

- Cvg is the mean coverage for each sample.
- Array is the percentage of agreement with genotyping validation with OminChip.
