# Supplementary material for: Integrated systems analysis reveals a molecular network underlying autism spectrum disorders
Source: Mol Syst Biol. 2014 Dec 30;10(12):774. doi: 10.15252/msb.20145487 (PMC4300495; doi:10.15252/msb.20145487)
Supplement: Supplementary file 18 [file msb0010-0774-sd18.pdf]

Table S4. Ti/Tv ratio and mean coverage for exome-sequencing

ID	111289	111290	111292	111296	111299	111304	111310	111311	111313	111316	111317	133328
Ti/Tv	3.27	3.18	3.15	3.19	3.18	3.13	3.17	3.18	3.21	3.16	3.19	3.18
Cvg	107.95	110.68	115.59	120.55	113.75	111.03	202.3*	106.51	125.08	108.86	123.45	103.55

ID	133341	133344	133346	4899	4999	5144	5302	5308	5403
Ti/Tv	3.19	3.18	3.19	3.14	3.16	3.14	3.22	3.1	3.22
Cvg	130.47	97.07	120.41	117.97	113.14	120.11	124.26	112.57	127.27

Notes: 1. Cvg is the mean coverage for each sample.

2. Every two samples were sequenced in one HiSeq lane, and the sample 111310 were sequenced alone in one lane, which doubled its coverage. Cvg is the mean coverage for each sample.

3. The sample 111311 was not analyzed in this study since this is a non-European individual.
