# Supplementary material for: Integrated systems analysis reveals a molecular network underlying autism spectrum disorders
Source: Mol Syst Biol. 2014 Dec 30;10(12):774. doi: 10.15252/msb.20145487 (PMC4300495; doi:10.15252/msb.20145487)
Supplement: Supplementary file 19 [file msb0010-0774-sd19.pdf]

Table S5. Sample Information for RNA-sequencing

Case ID	Age	Sex	PMI	Ethnicity	Ctl ID	Age	Sex	PMI	Ethnicity
5403	16	M	35	European	5407	16	M	33	European
5144	7	M	3	European	5391	7	M	12	European
5308	4	M	21	European	4670	4	M	17	European
5302	16	M	20	European	5242	15	M	9	European
4899	14	M	9	European	5163	14	M	12	European
4999	20	M	14	European	4727	20	M	5	European
