# Supplementary material for: Integrated systems analysis reveals a molecular network underlying autism spectrum disorders
Source: Mol Syst Biol. 2014 Dec 30;10(12):774. doi: 10.15252/msb.20145487 (PMC4300495; doi:10.15252/msb.20145487)
Supplement: Supplementary file 20 [file msb0010-0774-sd20.pdf]

Table S6. Genes showing extreme expression difference (FPKM) in at least one matched case-control pair(s)

Symbols	case-4899	ctl-5163	case-4999	ctl-4727	case-5144	ctl-5391	case-5302	ctl-5242	case-5308	ctl-4670	case-5403	ctl-5407	Case-Ctl pairs showing diff. expresion
	AGE-14	AGE-14	AGE-20	AGE-20	AGE-7	AGE-7	AGE-16	AGE-15	AGE-4	AGE-4	AGE-16	AGE-16	
ACTN2	8.17378	6.91817	7.05457	7.6857	4.85415	12.3486	7.46995	13.5616	7.54513	9.69953	10.2076	6.55199	5144_5391
ATP2B2	10.4157	10.2315	14.496	2.27459	4.07595	7.84837	1.95087	1.29592	0.957262	12.9955	2.83299	6.72334	4999_4727 5308_4670
BCAS1	54.125	59.9034	57.4663	80.9105	45.298	63.5851	77.5107	97.8034	100.546	49.4591	83.5928	62.3803	5308_4670
CAMK2A	12.4896	17.5579	24.9935	3.8031	10.5568	11.6632	1.94691	2.23104	1.62797	29.614	1.83633	6.98267	4999_4727 5308_4670 5403_5407
CNTNAP4	34.1099	37.2074	52.8401	65.2879	27.0515	35.0873	18.6162	81.2807	68.3625	29.7576	44.5378	29.4967	5308_4670
DGKZ	6.60179	4.92138	7.28702	2.62397	3.08711	7.22943	2.03579	2.42599	2.02202	14.8028	1.94708	3.19701	5144_5391
DLGAP2	0.880738	0.85067	0.866568	0.315409	0.466513	0.964308	0.157345	0.154299	0.079893	1.66111	0.085811	0.351003	5308_4670
DLGAP3	1.79048	1.13156	0.707896	0.26221	0.446388	1.09454	0.274824	0.117639	0.132243	1.7777	0.168847	0.8147	5144_5391 5308_4670
DYNLL1	76.9804	100.479	114.349	147.13	112.733	75.688	18.2117	133.366	95.5138	100.619	67.9696	44.878	5302_5242
GDA	5.42175	4.82084	10.0478	2.45284	4.35218	9.22799	0.365664	1.55957	0.852387	24.4891	1.01248	1.69001	4999_4727 5308_4670
GRIA1	6.5753	5.24132	6.42643	3.39868	4.6167	5.57764	0.754103	2.04652	1.14055	14.8272	2.1878	2.13048	5308_4670
GRIK3	1.55601	0.80589	1.57387	0.922747	0.631652	2.19164	0.158799	0.79529	0.647623	3.31867	0.60924	0.897166	5144_5391
GRIN2A	5.43702	5.23169	12.1565	3.92986	3.49946	5.99765	1.41466	2.73144	0.994802	13.798	3.74731	3.22638	5308_4670
GRIN2B	6.06032	3.73538	5.5023	1.38787	2.89003	4.81559	0.350852	1.0746	0.910705	13.5725	0.71534	1.40033	5308_4670
HTR2C	2.48281	19.0262	0.766865	0.64221	0.352887	0.363268	0.381782	0.629359	0.117263	1.48519	22.7309	31.4639	4899_5163 5308_4670
KCNA4	1.37102	0.51685	0.78718	0.265734	0.439702	0.572336	0.066066	0.078082	0.054512	1.17392	0.144732	0.318205	5308_4670
KCNJ2	4.97208	11.6348	13.5834	18.9721	19.7206	17.288	7.30111	24.2115	21.5645	11.8395	11.7479	5.37666	4899_5163
KCNJ4	1.07885	0.8044	1.28626	0.252741	0.396522	0.785802	0.261297	0.15163	0.119609	2.2375	0.120816	0.295969	4999_4727 5308_4670
LDB3	2.6986	6.11025	3.73788	5.926	2.29005	8.6733	4.31096	8.16793	8.58386	4.77803	7.49784	4.55085	5144_5391
LPL	7.36867	1.50055	1.39253	1.42135	2.63802	0.621972	1.45769	1.32159	0.988848	0.84403	1.51843	2.22148	4899_5163
NRXN2	4.87073	5.95951	4.89019	4.46779	3.49419	9.54515	3.97993	5.68091	6.32916	9.73752	5.83865	5.11209	5144_5391
PGM5	0.479049	1.85635	2.03931	2.67337	1.90154	1.25966	0.755508	1.98993	1.87654	1.78705	1.34142	0.358495	4899_5163 5403_5407
PTPRN	3.47961	2.13605	2.92521	0.491405	1.04898	2.90347	0.181454	0.30846	0.251279	6.38684	0.303596	0.94505	4999_4727 5144_5391 5308_4670
S100A3	2.56913	0.11203	0.060789	0.081383	0.076386	0.03406	0.178854	0.257424	0.050422	0.04706	0.137539	1.23705	4899_5163 5403_5407
SCN1A	5.38941	6.30019	15.8115	2.74602	3.16211	3.092	1.8294	2.09287	2.05957	5.82705	2.78413	2.50353	4999_4727
SHANK2	1.66051	1.73733	1.18268	0.630883	1.05491	1.45654	0.609728	0.376086	0.269225	3.5049	1.36204	2.77783	5308_4670
SHANK3	1.24115	1.35859	0.594706	0.449257	0.537711	1.45191	1.10618	0.413875	0.473776	1.51758	0.684206	1.44919	5144_5391 5302_5242
TBR1	1.46978	1.27778	2.82477	0.373084	0.989652	2.2465	0.218025	0.129242	0.108868	2.84041	0.191095	0.399778	4999_4727 5144_5391 5308_4670
TJAP1	2.31436	4.58465	3.07065	4.04476	2.77386	6.35728	2.97949	5.82153	5.31939	3.97288	4.89807	3.74644	5144_5391
ZDHHC23	1.39532	1.2062	3.06164	0.445495	0.987482	1.37944	0.45738	0.282604	0.366755	2.76498	0.825861	1.17759	4999_4727
