# Supplementary material for: Integrated systems analysis reveals a molecular network underlying autism spectrum disorders
Source: Mol Syst Biol. 2014 Dec 30;10(12):774. doi: 10.15252/msb.20145487 (PMC4300495; doi:10.15252/msb.20145487)
Supplement: Supplementary file 21 [file msb0010-0774-sd21.docx]

**Supplementary Legends**

**Supplementary Figures**

**Fig. S1. The flowchart of the study design.** This study first uncovered an ASD-related module, followed by validation among ASD patients, and by functional characterization using network and transcriptome analyses. In the Discovery panel, the red nodes are genes previously known to be associated with ASD. In the Integrative analysis panel, blue and red nodes represent excessive mutation and differential expression for a given gene in the network. (MRI image of the corpus callosum: Allen Institute of Brain Science)

**Fig. S2. Co-expression of the interacting proteins.** A comparison among randomly sampled protein pairs (*random* on the x-axis), HINT(Das & Yu, 2012; Wang et al, 2012) (a recently benchmarked protein interaction network) and BioGrid revealed that interacting proteins in BioGrid have the highest expression correlation among the 79 human tissues and cell types(Su et al, 2002).

**Fig. S3. Topological properties of the network modules. (A). Cluster size distribution follows a power-law.** The inset of the histogram is a log-log plot for the cluster sizes showing a significant scale-free property. **(B). The elevated network modularity Q for the real human protein interaction network.** A set of 100 randomized networks was generated to determine the statistical significance. The random simulation preserved the number of interacting partners for each node but randomly rewired the interactions.

**Fig. S4. (A) FDR distribution of GO enrichment for the protein modules.** The vast majority of the modules are highly significant with FDR<5e-3. **(B) The threshold selection to determine the size of the clusters showing GO enrichment.** The number of enriched clusters plotted against the size threshold varied from 1 to 20 (the blue line with circles). The gradient of the blue line at each threshold was shown in black (with squares). We chose to use n=5 as a threshold in our analysis, which represented a transition point from a rapid increase of the gradient towards full convergence.

**Fig. S5. Hierarchical clustering of the enrichment map for the topological modules.** Red pixels indicate GO term enrichment (arranged along the horizontal axis) for each module (arranged along the vertical axis). Exemplar terms are also highlighted in the map. The right panel depicts the enrichment (false discovery rates, FDRs) of each module for a collection of known autism genes. Insignificant FDR is set to 1, and the two autism-associated modules (module #2 and #13) are enriched for transcriptional regulation and neuron synaptic transmission, respectively.

**Fig. S6. Full spectrum of the enriched terms and their distribution in module #13.** The most enriched term is synaptic transmission, and its related GO terms indicate that module #13 contains over-represented genes regulating the cascade reaction of synaptic transmission. The horizontal axis indicated the number of genes in the module belonging to each term marked on the vertical axis. Percentage on each bar indicates the percentage of the module genes compared to all the genes associated with the same term. ClueGO was used to perform this GO analysis (http://www.ici.upmc.fr/cluego/).

**Fig. S7. Enrichment for the ASD genes in this module #13.** The enrichment tests were performed on the known SFARI ASD genes from different releases. The newly added genes were those from Sep. 2012 to Jul. 2013, representing the growth of our knowledge.

**Fig. S8. Absolute expression of genes in the 2 groups across 295 brain sections.** The median of each group in each brain section (in black) was compared with the transcriptome median (shared by both groups, in green). The zoom-in view shows an elevation of gene expression of Group 1 genes in the corpus callosum, where Group 2 genes were down-regulated. The overall expression of Group 2 genes was all above the transcriptome background (in green) across all the brain sections, indicating its ubiquitous expression.

**Fig. S9. Expression propensity for the two gene groups.** Increased expression specificity index for genes in Group 1 (**A**) is consistent with its reduced expression breadth (**B**). Expression breadth is defined to be the number of the brain sections where a gene is expressed. Three cutoffs were used to determine the absence/presence of a gene in a tissue, representing the 5%, 25% and the 50% percentiles of the expression data across all genes and all sections.

**Fig. S10. The biased expression of LRP2 in the corpus callosum.** RNA-sequencing for the Brodmann areas 9 (BA9), 40 (BA40), and the amygdala (AMY) was from a typically developing individual. LRP2 expression in the corpus callosum (CC) was evaluated based on its expression across the six non-diseased subjects in this study.

**Fig. S11.** **Immunohistochemistry analysis of LRP2 in the human corpus callosum**. (**A**) A control subject was immuno-stained with anti-LRP2, whose specificity was determined by a positive control and two sets of negative control (B-D). **(B)** The positive control was the staining in the human kidney carcinoma, which has been known to have extremely high LRP2 protein levels. **(C)** IgG staining in the corpus callosum was used as the first negative control. **(D)** LRP2 staining in the normal human ovary was used as the second negative control, where the absence of LRP2 has been indicated in literature.

**Fig. S12. Pearson’s correlation between 2 biological replicates of RNA-seq experiments.** Biological replicates were performed on 6 samples, where different sections from the tissue blocks were assayed. Genes with extreme expression (FPKM>50, accounting for less than 1% of the genes in the human genome) were excluded from the analysis.

**Fig. S13. The layered structure of the protein interaction network in this study.** *K*-core decomposition was used to partition the network. The visualization was implemented by LaNet-vi. Node colors followed the rainbow color scale, with violet for the most peripheral nodes (*K*=1), and red nodes for the greatest *K* in the network.

**Fig. S14. The cumulative distribution of the node coreness in the network.** In this analysis, we considered nodes in the network center with *K*≥10, where >80% of proteins in the network were below this threshold.

**Supplementary Tables**

**Table S1A.** Validation for the enrichment of ASD candidate genes in this module.

**Table S1B.** Enrichment tests for genes in module #13 affected by different types of mutations. In each test, genes in module #13 were compared with a set of randomly sampled control genes (protein-coding), whose CDS length and GC content is indistinguishable from those of genes in module #13. Genes affected in ASD probands and non-ASD individuals were both compared. Mutations identified from ASD probands were highlighted in red. P values were derived from Fisher’s exact test.

**Table S2.** Sample Information for DNA-sequencing.

**Table S3.** Information for whole-genome sequencing.

**Table S4.**The Ti/Tv ratios and the mean coverage for exome-sequencing data in this study.

**Table S5.** Sample Information for RNA-sequencing.

**Table S6.** Genes showing extreme expression differentiation in at least one matched case-control comparison(s).

**Supplementary Datasets**

**Supplementary Dataset S1.** The global network and gene membership in each topological module on the network.

**Supplementary Dataset S2.** The enriched GO terms for each network modules. The complete lists of genes implicated in ASD from were also separately listed.

**Supplementary Dataset S3.** Information of the variants identified in this study. Their overlap with SFARI genes or the dbGAP exome-sequencing data was also listed. 28 genes in the screen were novel, and 10 of the 28 genes showed abnormal behavior (neurological) or a defective nervous system in their respective mouse mutants (from MGI annotation).

**Supplementary Dataset S4.** Expression sub-groups of genes in module #13. Group 1 genes were highly expression in the T1 regions, and Group 2 genes were more expressed in T2 regions. The individual brain region and gene membership in each sub-group was separately listed.
