# Supplementary material for: Integrated systems analysis reveals a molecular network underlying autism spectrum disorders
Source: Mol Syst Biol. 2014 Dec 30;10(12):774. doi: 10.15252/msb.20145487 (PMC4300495; doi:10.15252/msb.20145487)
Supplement: Supplementary file 26 [file msb0010-0774-sd26.pdf]

molecular systems biology

Integrated Systems Analysis Reveals A Molecular Network Underlying Autism Spectrum Disorders

Jingjing Li, Minyi Shi, Zhihai Ma, Shuchun Zhao, Ghia Euskirchen, Jennifer Ziskin, Alexander Urban, Joachim Hallmayer and Michael Snyder

Corresponding author: Michael Snyder, Stanford University

Review timeline:

Submission date:	10 June 2014
Editorial Decision:	22 July 2014
Revision received:	06 October 2014
Editorial Decision:	17 November 2014
Revision received:	27 November 2014
Accepted:	03 December 2014

Editor: Maria Polychronidou

Transaction Report:

(Note: With the exception of the correction of typographical or spelling errors that could be a source of ambiguity, letters and reports are not edited. The original formatting of letters and referee reports may not be reflected in this compilation.)

1st Editorial Decision 22 July 2014

Thank you again for submitting your work to Molecular Systems Biology. We have now heard back from the three referees who agreed to evaluate your manuscript. As you will see from the reports below, the referees acknowledge that the presented approach and findings are potentially interesting. However, they raise a series of concerns, which should be carefully addressed in a revision of the manuscript.

Without repeating all the comments listed below, some of the more fundamental issues are the following:
- The statistical significance of the presented findings needs to be carefully documented.
- Including of further analyses on module #2 will enhance the impact of the study.
- The presented results/conclusions should be better placed in the context of existing literature and previous findings.
Moreover, the reviewers point out that the manuscript needs to be carefully re-written in order to make the key findings easily accessible and to ensure that the methods are clearly described.

Reviewer #1:

The paper by Li et al. report the identification of a specific molecular network associated with autism spectrum disorders, through integration of genetic, genomic, transcriptomic, interactomic, functional and clinical data. The overall evidence provided is convincing and well documented through appropriate gigures and detailed, extensive supporting information. The integrative

© European Molecular Biology Organization

approach developed by the authors, associated with sound and systematic statistical significance testing of alternate hypotheses, allows them to overcome the limitations seen in previously published studies in which the focus was mainly on genetic mutations, and failed to uncover the underlying mechanisms. In that respect, the paper will be of interest not only to the specialists of autism and related disorders, but to a broader audience of readers who might be interested to take a simmilar approach for other complex diseases.

The paper however requires substantial editing and improvements in its presentation on several important points:

1- The authors leveraged a wealth of previously published and publicly available data sets, and added very significant experimental contributions. While this is precisely documented in the method section and the supporting information, it is not immediately clear from the Abstract and not always in the text. It is important that the authors indicate what their direct contribution to the experimental datasets was, and that they emphasize the value of exisiting datasets, as this is part of the power of their approach, and will provide an incentive for other investigators to both share openly their datasets, and leverage those produced by others.

2- The paper focuses mainly on module #13. It would be of interest that the authors add a short section on module #2 which contains a large number of transcription factors, and at least explain how they envision exploring it functionally. In addition, from Figure 1, it appears that there are other modules such as #4 that seem to be also enriched in autism-related genes. The authors should mention this and explain the rationale they used to define a threshold of enrichment, if any.

3- The transcriptomic evidence presented in Figure 3 and page 10 is not as clear cut as it seems from the text with regard to general or tissue-specific expression. In both groups of genes, the heat map sections are very heterogeneous, and there are in both cases quite a number of individual genes that do not follow the general pattern of their group. In that respect, the overemphasis of the trends is not sufficiently supported by the experimental evidence and should be either discussed more precisely or toned down.

4- Similarly, the evidence presented in Figure 4B and discussed on page 11 only points to overexpression of subsets of genes in oligodendrocytes, while failing to discuss large groups of genes that are overexpressed in neurons (bottom left corner of 4B) or in astrocytes (middle-right of 4B) and underexpressed in oligodendrocytes. The authors should discuss these groups and explain the rationale for not taking them into account as seriously as the others as possible contributors to the mechaisms of autism-related disorders.

5- The authors mentioned in passing two genes that appear as outliers in Fig. 5 D (DYNLL1 and BCAS1) with regard to their expression patterns. They should discuss whether or not this has functional implications.

6- The authors have put a great deal of emphasis on the potential role of the LRP2 gene and protein overexpression as an important component of module #13 implication in the patophysiology of ASD. Yet, it does not appear in the the list of genes showing extreme expression differences in the matched case-control validation experiments reported in Table S6. This could mean that LRP2 is less important and/or that other genes/proteins listed are also important contributors, and this should be properly discussed by the authors.

7- The manuscript is overall well written and clearly structured, although in some sections rather lenghty and technical. Although this is largely unavoidable due to the complexity of the approach and the requirements for careful assessments of confounding factors, the manuscrit could benefit from a comprehensive and more concise presentation, associated with careful English syntax editing.

Reviewer #2:

Summary: In this very extensive and well-done study, Li and colleagues construct an interaction network from human protein interaction data. They find and validate an autism-associated module

with gene expression and genome sequencing data. They delve into the biology of this module and find enrichment for oligodendrocyte and corpus callosum expressed genes, implicating these cells and this neuroanatomical region in autism pathogenesis.
More specifically, Li and colleagues look at the BioGRID human protein interactome data as a network, and verify the quality of the network by evaluating its agreement with other data, including RNA co-expression data. They apply a topological clustering analysis to identify 817 modules, and global modularity in the observed interaction network is convincingly demonstrated with an edge-shuffling approach that retains global topological relationships. 85 of the identified modules show enrichment for a previously annotated gene ontology terms. Out of these modules, one (Module #13, 119 genes) was enriched for known ASD genes taken from the SFARI database. This enrichment was robust to permutations controlling for GC content and gene length. Intriguingly, Module #2, which was related to DNA-dependent transcription, also showed enrichment for ASD genes.
The authors then extensively validate Module #13's relationship to autism. They show that the enrichment for Module #13 was strong and not entirely driven by trivial factors such as gene list bias or synaptic genes and demonstrate strong enrichment from high-confidence CNV-affected genes and weaker enrichment for larger CNV and SNV gene sets. The authors also show that Module #13 is enriched for autism genes more than schizophrenia genes, and not enriched at all for Alzheimer's disease genes. These analyses show specificity of the module toward autism-related genes from multiple sources. Taken together, the authors convincingly identify an autism-relevant module in the available human protein interactome.
The authors next use data from 25 sequenced autism patients, and find 113 nonsynonymous mutations in Module #13 to be weakly enriched over the exome background at $p = 0.02$. They show that the preference of these mutations for Module #13 was not strongly affected by gene length. Based on recurrently observed mutations in LRP2, an initial lead to the corpus callosum is suggested. This is followed up on later. Additional comparisons to larger patient cohorts demonstrated that 38 module-specific candidate genes were enriched for mutations, though at moderate statistical significance (uncorrected $p < 0.05$, but $p > 0.01$ in many cases).
The rest of the manuscript follows the LRP2 lead and attempts to dissect the cellular and neuroanatomical relationships implicated by Module #13. First, it is shown that Module #13 can be split into two groups co-expressed across human brain regions using the Allen Brain data, with one more ubiquitously expressed (group 2), and the other expressed most highly in corpus callosum (group 1). Next, this enrichment for corpus callosum is validated by the authors' own RNA sequencing and further confirmed by immunohistochemistry to confirm LRP2 and oligodendrocyte presence in the corpus callosum. Next, to home in on cell-types, the authors use mouse cell-type data to implicate oligodendrocytes as affected by group 1 and group 2 and combine mouse oligodendrocytes culture and MRF knockout data to suggest that the up-regulation of group 1 and down regulation of group 2 is essential to proper oligodendrocytes development. Finally, RNA-seq from the corpus callosum of ASD patients is used to show that, taking the union of dysregulated genes across patients, Module #13 genes are more affected compared to synaptic genes or broader autism genes. Most of these enrichments were marginal in effect size at the gene set level, but enough data is provided to put forth a compelling story.

General remarks: It seems that authors are trying to drive home three major points: 1) the protein interactome in humans is modular, and that this structure can be used to better understand autism, 2) a protein-interaction module could help prioritize mutations from whole exome and genome studies, and 3) protein-interactions implicate oligodendrocyte maturation and corpus callosum development in autism. Extensive validation is performed, which provides further experimental evidence supporting these three major points. This study therefore will be of widespread interest.

However, the claims of novelty or uniqueness as put forth by the authors are less convincing and one wonders why this elegant work is not more integrated with other published work. A simple literature search reveals multiple studies that have identified interaction networks of various sorts implicating specific neurodevelopmental pathways in autism (Ben-David and Shifman, 2012b; Gilman et al., 2011; Parikshak et al., 2013; Willsey et al., 2013), and several have used protein interaction networks in a variety of ways (Corominas et al., 2014; Cristino et al., 2014; Sakai et al., 2011), many of which are not cited. The value of this study is not for its "novel" use of genome wide interaction data per se. Rather, the study does employ a unique combination of previously used approaches in a manner that is of very high quality and that is more convincing than some of the previous studies and is quite complementary to others - this latter point needs more emphasis. It also delves deeply into the biology of one of the network components, and arrives at a convergent

pathway in autism. But one is left asking: What about ASD that has been observed in these previous studies can this work confirm or validate, and what does it not validate?
This work will be most interesting for neuroscientists and geneticists studying neurodevelopmental disorders, particularly autism. For this audience, it would be ideal for the authors to clearly demonstrate how the module identified here relates to the existing work described above. This is discussed in detail in the major points below. There are also technical concerns that should be addressed to give readers more confidence in the soundness of the work.

Major points:
- Grammar needs some checking - there are problems with articles and plurals throughout the paper that detract attention, starting in the Introduction.
- The major novelty of this study as described in the manuscript originates, as the authors state, from identifying a "naturally occurring pathway underlying this disease" instead of starting from mutations as has been done in other studies cited: (O'Roak et al., 2012; Willsey et al., 2013).
o However, others have used "naturally occurring" pathways defined at the genome-wide transcript co-expression level to do similar types of analyses (Ben-David and Shifman, 2012b; Parikshak et al., 2013)
o They have also been identified using a "seeding-and-expansion" approach at the protein interaction level (Cristino et al., 2014), which also uses a clustering algorithm to identify shared relationships (though the work here is more genome-wide and considerably more statistically sound, again a comparison that can be discussed by the authors)
- The novelty in this study came across to this referee from the following:
o Implication of the corpus callosum, however note that
- Previously, Ben-David et al. 2012 identified corpus callosum modules using the same anatomical expression data, and a connection to immature oligodendrocytes was also made. They had a "black" and "yellow" co-expression module that were both highly expressed in this region, but they did not find strong evidence that these were affected by the autism risk genes they assessed.
- The authors should at least cite that study and ideally compare Module #13 to these two modules.
- Parikshak et al. also describe enrichment for ASD genes (based on RNA expression) in neurons that project intra and interhemispherically in layers 2/3 of monkey cortex that comprise virtually all of the collosal projecting fibers in primates. This finding therefore would be consistent with the current analyses presented here and this should be emphasized as it uses a very different approach to come to a similar condition. This is also consistent with a model of cortical disconnection, which is the first such study since Parikshak, to show this. Furthermore, the data here are somewhat stronger in this regard, so these findings could be considered a significant extension of this notion.
- The RNA profiling in corpus callosum is the first such study and provides strong experimental support. The enrichment analysis with control gene sets is elegant.
o Usage of new genomic variant data to validate the network
- Recent work has done this with the above-mentioned protein interaction based "seeding-and-expansion" approach (called AXAS) with exome sequencing in trios, suggesting An et al. 2014 could group ASD-associated mutations in families - the current work should discuss how it differs or agrees with the An et al. work (An et al., 2014)
o I think the authors have made greater advances in both of these areas relative to the above two studies, and they should emphasize the strength of their work in that context rather than only its novelty. In reality the strength of this study is that it is very well done and has solid statistics and assumptions and validation.

- The authors control for potential biases related to gene length and GC content as done in previous work (O'Roak et al., 2012; Willsey et al., 2013). However there is substantial additional biases to consider when using global but incomplete protein interactome data that are curated from multiple studies and whole exome / whole genome data for enrichment analyses:
o Protein interaction networks in human are incomplete. Other than the fact that they are not tissue or time specific, it is estimated that there are 150K to 370K total interactions in the human proteome Hart et al., 2006), and only 69113 could be assessed here (ignoring inaccuracies in the interaction data). Therefore, only 20-50% of the putative human interactome is measured. Furthermore, even in yeast, even when ~50% of the interactome was measured, a study showed that the global topological structure of the interactome is highly unstable, depending on what filtering steps are taken to include protein interactions and the fact that the most studied genes have the most interactions (Hakes et al., 2008). It is likely that these issues highly affect the future generalizability of the networks presented here, particularly the high modularity (>800 modules) observed. Here are two suggestions to be

more transparent about this bias:
- The authors could try to model the effect of bias in their permutation analyses. See Rossin et al., 2011 for details (Rossin et al., 2011). They could enact a better permutation scheme by including, in addition to GC content and CDS length (or in lieu of these factors), genes that have a similar distribution in the global number of protein interactions in the global network. If Module #13 is not driven by biases in the overall interactome, then the calculated p-values should remain significant.
- The authors could use recent work deeply querying a subset of the interactome (Corominas et al., 2014), which likely overlaps with Module #13. These interactions could be used to estimate how biased the current network structure might be, and how complete it is relative to a known "complete" subset.
o The authors controlled for GC content and CDS length in enrichment for the initial SFARI gene list in Module #13 by permuting random sets matched for these factors.
- However, they seem to not use this permutation scheme for some of the later enrichments where these factors are actually playing the greatest biasing role - with the genetic variants from exome and whole genome studies. The GC content/CDS length permutation approach should be used throughout the study, rather than relying on constructing "matched" backgrounds or doing Wilcoxon tests for contributions from gene length - these are less stringent approaches and it becomes convoluted when different approaches are used at different points in the manuscript.
- The authors find enrichment for genes expressed in the oligodendrocytes and the corpus callosum as seen in previous work (Ben-David and Shifman, 2012b)
o A concern here is that the preference for the corpus callosum and oligodendrocytes over other regions shown in Figure 3 and 4 is marginal. The authors should discuss the reason why group 1 specificity for the corpus callosum and oligodendrocytes is significant, but not very striking, and discuss what other cells and regions might also be involved
- The sequencing study in 25 patients identifies 38 candidate loci, mostly missense. Much larger exome sequencing studies have shown these classes of mutations to occur equally in probands and controls essentially, so the genetic evidence presented here is not convincing that they have detected 28 new (not described previously) genes. They would need to compare case-control burden doing a proper case-control study as is standard in human genetics. This is consistent with the modest increased burden in module 13. Is this corrected for multiple comparisons? The use of dbGAP samples helps bolster their claim. Perhaps focusing on the 14 genes from among the original 38 for which there is some additional support is more appropriate than their current emphasis.
- Currently the manuscript is not clearly placed in a quantitative context with the published systems level work that already exists in ASD, but this could be easily done. To help orient readers, I have a few additional suggestions (not necessary to address as module-level data may not easily be available from all of the studies - more important to address the data sharing minor point):
o Compare Module #13 with the results from the AXAS (Cristino et al., 2014) and NETBAG (Gilman et al., 2012; 2011) approaches that also heavily rely on protein interactions.
o Directly compare with more transcriptomically-driven modules from Ben-David et al (Ben-David and Shifman, 2012b), Parikshak et al (Parikshak et al., 2013), Willsey et al (Willsey et al., 2013), and/or Liu et al (Liu et al., 2014)
o Check the developmental time course of the identified module on BrainSpan - though may not be helpful since the BrainSpan database does not contain corpus callosum. Other studies have done this by simply taking normalized expression levels for the module and assessing plotting them for different regions (Ben-David and Shifman, 2012a; Gilman et al., 2012; Gulsuner et al., 2013).

- Module #2 seems very interesting. It appears as if some de novo variant affected genes are in this module, and it could be useful to further study or at least compare with previous work cited here - otherwise the focus on module 13 seems a little arbitrary. The authors note that module #2 is less significant than module #13, but it is also 10x larger than the module 13, which certainly could affect enrichment scires. Also module 13 is enriched for synaptic function, which has been studied extensively and thus the relative enrichment may at least partially reflects this bias. Why is this #2 module so big - does it actually correspond to multiple true modules that reflect different aspects of tissue or developmental time co-expression? For instance since PPI is typically defined outside of cell or tissue or developmental time context, might this large PPI module (module 2), really reflect multiple distinct in vivo functional modules that could be disentangled by looking at tissue and developmental time specific co-expression based on RNA?
- In the same vein, the separation of the transcription factor and synaptic functions into distinct modules does coincide with O'Roak et al. and some of the other published studies cited above. All of the enrichment and comparisons are done with module 13 and it would be important to at least

include those analyses for module 2 in supplemental tables.

Minor points:
- Figure 1 would be improved by including Module #13's enrichment not just for the SFARI genes, but also for some of the other gene sets queried
- Figure 2A is difficult to interpret - what exactly are the authors trying to show? Perhaps it would help to show where genes in other modules cluster on this plot, which would contrast how these genes cluster in this feature space compared to other genes?
- Citations are lacking for "two independent studies" that have implicated the upper and lower cortical layers in autism
- Data sharing: In the supplemental tables, the authors should provide a table containing each protein and the module that contains it. This information will allow others to use the network in future work for comparison and further validation. In supplemental data, the authors should include the exact protein interactions used in the study from BioGRID. These are easily done but essential steps for future usability of these analyses. Finally, the RNA-seq data used here should be made available via SRA and GEO.
- A more descriptive title emphasizing the relationship to the corpus callosum would be helpful
- Introduction: The statement that the known mutations "explain" 10-20% is not really clear. They may be found in that percentage of patients, but given reduced penetrance that has been demonstrated for many, including major gene disrupting CNV, they explain much less of the known genetic variance contributing to ASD.
- The schizophrenia overlap in module #13 is somewhat glossed over (page 7). This is very interesting and should also be emphasized as it is an emerging area of interest to the field. A difference in enrichment percentage may just reflect that more ASD genes are known with higher certainty than SZ genes.

Overall perspective: On the whole, this is an important study that extends our understanding of the molecular mechanisms that contribute to autism. My points above related to a) bias and b) comparability and overlap with other published studies should be addressed to give readers more confidence and better context for this work. But, despite my detailed comments, these additional analyses should only require minor revisions, mostly related to writing and emphasis.

References:
An, J.-Y., Cristino, A.S., Zhao, Q., Edson, J., Williams, S.M., Ravine, D., Wray, J., Marshall, V.M., Hunt, A., Whitehouse, A.J.O., et al. (2014). Towards a molecular characterization of autism spectrum disorders: an exome sequencing and systems approach. Transl Psychiatry 4, e394.
Ben-David, E., and Shifman, S. (2012a). Combined analysis of exome sequencing points toward a major role for transcription regulation during brain development in autism.
Ben-David, E., and Shifman, S. (2012b). Networks of Neuronal Genes Affected by Common and Rare Variants in Autism Spectrum Disorders. 8, e1002556.
Corominas, R., Yang, X., Lin, G.N., Kang, S., Shen, Y., Ghamsari, L., Broly, M., Rodriguez, M., Tam, S., Trigg, S.A., et al. (2014). Protein interaction network of alternatively spliced isoforms from brain links genetic risk factors for autism. Nat Comms 5, 3650.
Cristino, A.S., Williams, S.M., Hawi, Z., An, J.-Y., Bellgrove, M.A., Schwartz, C.E., da F Costa, L., and Claudianos, C. (2014). Neurodevelopmental and neuropsychiatric disorders represent an interconnected molecular system. Molecular Psychiatry 19, 294-301.
Gilman, S.R., Chang, J., Xu, B., Bawa, T.S., and Gogos, J.A. (2012). Diverse types of genetic variation converge on functional gene networks involved in schizophrenia.
Gilman, S.R., Iossifov, I., Levy, D., Ronemus, M., Wigler, M., and Vitkup, D. (2011). Rare de novo variants associated with autism implicate a large functional network of genes involved in formation and function of synapses. 70, 898-907.
Gulsuner, S., Walsh, T., Watts, A.C., Lee, M.K., Thornton, A.M., Casadei, S., Rippey, C., Shahin, H., Nimgaonkar, V.L., Go, R.C.P., et al. (2013). Spatial and Temporal Mapping of De Novo Mutations in Schizophrenia to a Fetal Prefrontal Cortical Network. 154, 518-529.
Hakes, L., Pinney, J.W., Robertson, D.L., and Lovell, S.C. (2008). Protein-protein interaction networks and biology--what's the connection? Nat Biotechnol 26, 69-72.
Hart, G.T., Ramani, A.K., and Marcotte, E.M. (2006). How complete are current yeast and human protein-interaction networks? Genome Biol 7, 120.
Liu, L., Lei, J., Sanders, S.J., Willsey, A.J., Kou, Y., Cicek, A.E., Klei, L., Lu, C., He, X., Li, M., et al. (2014). DAWN: a framework to identify autism genes and subnetworks using gene expression

and genetics. Mol Autism 5, 22.
O'Roak, B.J., Vives, L., Girirajan, S., Karakoc, E., Krumm, N., Coe, B.P., Levy, R., Ko, A., Lee, C., Smith, J.D., et al. (2012). Sporadic autism exomes reveal a highly interconnected protein network of de novo mutations. 1-7.
Parikshak, N.N., Luo, R., Zhang, A., Won, H., Lowe, J.K., Chandran, V., Horvath, S., and Geschwind, D.H. (2013). Integrative Functional Genomic Analyses Implicate Specific Molecular Pathways and Circuits in Autism. 155, 1008-1021.
Rossin, E.J., Lage, K., Raychaudhuri, S., Xavier, R.J., Tatar, D., Benita, Y., Constortium, I.I.B.D.G., Cotsapas, C., and Daly, M.J. (2011). Proteins Encoded in Genomic Regions Associated with Immune-Mediated Disease Physically Interact and Suggest Underlying Biology. 7, e1001273.
Sakai, Y., Shaw, C.A., Dawson, B.C., Dugas, D.V., Al-Mohtaseb, Z., Hill, D.E., and Zoghbi, H.Y. (2011). Protein interactome reveals converging molecular pathways among autism disorders. Science Translational Medicine 3, 86ra49-86ra49.
Willsey, A.J., Sanders, S.J., Li, M., Dong, S., Tebbenkamp, A.T., Muhle, R.A., Reilly, S.K., Lin, L., Fertuzinhos, S., Miller, J.A., et al. (2013). Coexpression networks implicate human midfetal deep cortical projection neurons in the pathogenesis of autism. 155, 997-1007.

Reviewer #3:

Li et al. in their paper, "Integrated systems analysis reveals molecular network underlying autism spectrum disorders", carry out a series of integrative analyses and experiments over a diversity of data to uncover a subnetwork that is enriched for genes that harbor mutations that associate with ASD and that confirm processes and brain regions involved with ASD, but also uncover novel insights (both process, tissue type, and genes) into ASD. This manuscript addresses an important problem in modern disease research where many different types of data are being generated but then few groups really trying to integrate all such data to come up with comprehensive characterizations of disease. I think the authors strike a nice balance between leveraging very extensive existing data on disease and non-disease conditions and generating their own data and carrying out validation experiments. However, my main struggle with the paper is that it involved a long series of steps that more or less depended on one another to get to the claims made around module 13 and its role in ASD, but the results within these steps were not always clear or convincing on the statistical side, so that when one considers the propagation of error through the various steps, it is not clear how strongly supported the claims are. I've tried to highlight these issues in the specific comments below.

Specific Comments:

1. The approach taken by the authors while perhaps novel for autism is similar in spirit to network-based approaches taken over the last 5+ years. For example, in Plos Genetics e1000932 (2010) coexpression networks were constructed from different tissues and coexpression modules were identified and then novel loci were identified given modules identified as enriched for loci associated with disease in genome-wide association studies. Here protein interactions are used instead of gene expression and sequencing in place of GWAS, but again similar in spirit. There are a number of other instances of this across different diseases. It may be worthwhile putting this present work in context of what has been done already in different disease areas to help motivate why the particular choices made in this paper were made. For example, why protein interaction instead of gene expression? Protein interactions are typically not assayed in any relevant disease context, they are not scored in a population context, and so on, whereas gene expression is so assayed. On the other hand protein interactions are certainly critical to cell function, defining important mechanisms.

2. The approach proposed by the paper was made more difficult to follow given the figures were cited out of order (e.g., the mean expression correlation of expression traits with corresponding protein interactions was cited as supplementary figure 1 in the supplementary information, when in fact it is given as supplementary figure 2). In addition, the methods section is a bit of a mess. It appears to be just a series of thoughts strung together in sentences that are in a single paragraph but that span completely different analyses carried out. Maybe it was intended to be a summary, but it was very, very difficult to follow (e.g., the first several sentences talk about the network that was built, but then the transition from talking about the algorithm applied to build the network and the expression of genes in the Allen Brain Atlas is non-existent and so unclear whether one is talking about the network reconstruction or the assessment of the expression of genes in the module of interest across different brain regions).

3. Last sentence, page 4: the authors indicate a "novel parameter-free algorithm" to construct a network based on the protein interaction data, but in the supp information there is a couple of sentence given to describe the approach, referencing papers written 6 years ago on the method. I wouldn't characterize that as a novel algorithm.

4. In the first paragraph on page 6, the authors indicated that module #2 is enriched for genes known to associate with autism, but for this result they do not carry out the permutation testing they carried out for the module #13 result. Why is that? Does module #2 not demonstrate significance under such testing? If so that should be reported.

5. Throughout when the authors are quoting enrichment statistics from application of the Fisher Exact test, just the p value is being reported without indicating the actual statistic on which the p value is based or without some more intuitive statistic such as the fold-enrichment. Because the p value is correlated with sample size in this test, it is difficult to assess the meaningfulness of the result without knowing the counts and/or the fold enrichment on which the test is based. One could have a 1.1-fold enrichment with a really small pvalue if the sample size is very big, but that would be interpreted very differently than a really small pvalue with a 10-fold or greater enrichment. Even in the tables that report these enrichments, such as supplementary file 1, only FDRs are reported, with no count information, no enrichment statistics, etc.

6. On page 6, to support that module #13 is enriched for ASD genes beyond synaptic genes the authors test for enrichment of all non-synaptic genes. However, this would assume that all synaptic genes are 100% known, that the annotations used to identify synaptic genes are 100% accurate and complete. This is unlikely to be true. Couldn't it be that there are many unknown synaptic genes? Given the strong conclusion the authors are attempting to draw from this result, that "the ASD enrichment in module #13 cannot be attributed to only synaptic genes..." this seems an important point.

7. At the start of the second paragraph on page 7 the authors restrict to a set of 9,782 genes with CDS and GC content comparable to the module 13 genes. However, in the supplementary info, section 1 of the supp methods that they reference for this gene number, the authors indicate a set of 7,743 genes was identified. The results in the main text and in the supp methods should be made consistent.

8. In the second paragraph on page 7 the authors give several results reflecting the enrichment of ASD genes identified in human genetic studies in module 13. These results do seem to support the importance of this module in ASD, but there is not much information given to appropriately evaluate this, especially as some of the p values reported are only marginally significant at a 0.05 level. For the de novo and rare CNVs, and disruptive mutations used for these results (referenced in Table S1), it would be nice to have a table of the CNVs, the corresponding genes, some indication of how they were picked, then the genes that are mutated and how "disruptive mutation" were defined. While the reader could go digging through the papers referenced to try and figure this all out, one would still struggle to reproduce the results the authors indicate because the precise count information would be nearly impossible to reconstruct unless explicitly noted by the authors.

9. In second paragraph on page 8, authors indicate they identified 113 nonsynonymous mutations to genes in module 13 and then claim an enrichment of such mutations in this module, but the p value is only 0.021. Given the marginal significance of this enrichment, it is important to understand how many variants were identified overall, what was the exact selection criteria used to identify these variants (some info is given in the supp, but not complete) and then what was the actual expected versus observed counts. Further, the authors are using 1000 genomes as the basis for identifying rare mutations in the set they sequenced, but there are other resources available such as ESP, TCGA, etc. that could give a more comprehensive background estimate if combined.

10. I found the second paragraph on page 9 confusing. The results being presented claim replication of the candidate loci ostensibly from the WGS/WES sequencing carried out by the authors on the 29 or cases and controls. Were the variants tested the 113 loci that were localized to module 13? When the authors indicate that the "nonsynonymous variants with greater allele frequency differences between cases and controls...", does this mean such differences were statistically significant? And if

so what was the threshold used to declare significance and how was that established? Is the claim that while individual loci are not replicating, that the enrichments for the genes in the module are replicating? I think the authors need to be far clearer on what their primary hypothesis being tested is, how they are testing the null hypothesis corresponding to this, and why the test they are using is valid for this hypothesis. As it stands it appears the authors are using nominal significance thresholds (like 0.05), performing many individual tests, then performing tests on the results of those tests, and not really empirically estimating the null distribution through that entire process but rather doing some permutation here and there to support the results. It does appear there may be something really interesting here, but there are just too many missing details to see it.

11. Page 10, second paragraph, there are claims on the cluster depicted in Figure 3a of two groups being enriched for tissues associated with corpus callosum and neuron-rich regions, hippocampal formation, but no support is given for this claim. Some kind of enrichment test should be performed to support the claim. I think supplementary figures 8 and 9 kind of start to get at that, but it's not clear from this that whatever labels are on the brain regions being depicted are enriched for genes that are specific to different tissue types. It is true that subsequent experiments carried out like the staining do support the claim, but it is just unclear or at least confusing why the claim could be made in the first place from the clustering.

12. On page 14 the authors carry out RNAseq on brains from individuals with autism and test reproducibility of the expression data by sequencing multiple samples from the same brain. The correlation results depicted in supplementary figure 12 are used to argue high degree of reproducibility. While the correlations are very high, it would be of interest to carry out the same correlations across the different samples, both within the cases and between cases and controls, just to highlight that such correlation is driven by high intra-individual reproducibility that you do not see in "unmatched" samples.

1st Revision - authors' response 06 October 2014

Reviewer #1:

The paper by Li et al. report the identification of a specific molecular network associated with autism spectrum disorders, through integration of genetic, genomic, transcriptomic, interactomic, functional and clinical data. The overall evidence provided is convincing and well documented through appropriate figures and detailed, extensive supporting information. The integrative approach developed by the authors, associated with sound and systematic statistical significance testing of alternate hypotheses, allows them to overcome the limitations seen in previously published studies in which the focus was mainly on genetic mutations, and failed to uncover the underlying mechanisms. In that respect, the paper will be of interest not only to the specialists of autism and related disorders, but to a broader audience of readers who might be interested to take a similar approach for other complex diseases.

Our response: We sincerely thank this reviewer for appreciating the value of our work, and particularly for the insightful comments to improve our work. Below, in great detail, we list our revisions according to the reviewer's suggestions.

The paper however requires substantial editing and improvements in its presentation on several important points:

1- The authors leveraged a wealth of previously published and publicly available data sets, and added very significant experimental contributions. While this is precisely documented in the method section and the supporting information, it is not immediately clear from the Abstract and not always in the text. It is important that the authors indicate what their direct contribution to the experimental datasets was, and that they emphasize the value of existing datasets, as this is part of the power of their approach, and will provide an incentive for other investigators to both share openly their datasets, and leverage those produced by others.

Our response: We thank the reviewer for this suggestion, and we have substantially revised the text to highlight the experimental data that we have generated and also the publicly available datasets used in this study. Due to the word limit in the abstract section (175 words), we cannot list every data source in the Abstract, but we did try to highlight this better in the text. In particular, we also summarized the data we generated in our Discussion, as well as the sources of the public datasets used in this study. See below (pg. 19 in text) –

> *"We leveraged abundant genomic data including the human protein interactome, the transcriptome data in human and mouse brain, the MRF knockout data in mouse oligodendrocytes and also the mutation data from previous ASD sequencing projects. In addition, we also independently sequenced the genomes, exomes and transcriptomes in patients' brains to validate our observations from those publically available data or to gain new insights into this disease. Our integrative approach incorporated these genomic data of diverse dimensions, suggesting several key findings relevant to autism…"*

We agree with the reviewer that the field is now experiencing an exponential growth of the genomic data, and encouraging other investigators to extensively explore these sets of data will rapidly expand our knowledge.

2- The paper focuses mainly on module #13. It would be of interest that the authors add a short section on module #2 which contains a large number of transcription factors, and at least explain how they envision exploring it functionally. In addition, from Figure 1, it appears that there are other modules such as #4 that seem to be also enriched in autism-related genes. The authors should mention this and explain the rationale they used to define a threshold of enrichment, if any.

Our response: We thank the reviewer for this comment. Module #2 is indeed another very interesting module. It fits with the recently proposed "unexpected roles for chromatin (remodelers)" in autism (Ronan, J.L. et al. *Nature Review Genetics*, 2013), including CHD8. In addition, the language factor FOXP2, the Rett syndrome methylation gene MECP2 and its associated BAF complexes were all localized in this module. However, in this manuscript, we focused on module #13 simply because it gave the strongest enrichment (for SAFRI genes it was FDR=4.6e-11) relative to module #2 (FDR=2.3e-3) and other modules. Because the analysis of module #13 was extremely intensive, we did not feel that we could cover both in a single paper, and the analysis of module #2 will certainly dilute the topic of this paper.
However, we do agree with the reviewer that exploring module #2 will give additional insights into this disease, and in fact, we are working on another project involving a systematic exploration of genes in module #2. Unlike the overall association of module #13 in ASD, only a subset of TFs and chromatin remodelers in module #2 is strongly implicated in ASD, which has required us to use a different analytic framework to first identify these factors, followed by molecular characterization. Thus we feel that it is more appropriate to focus on module #13 in this paper, and module #2 itself deserves a separate study.

Module #4 was not significant because this module did not survive the multiple-hypothesis correction with FDR=0.52 (after correction). However, the reviewer indeed raised an interesting point here that despite a lack of strong enrichment for autism, we also tested the overall enrichment of these modules for any genes annotated to be disease-related (according to GeneCard annotation, ~3000 genes in total), and module #4 showed a substantial enrichment for the curated human disease genes with FDR=4.18e-7. This module is a signal-transduction module (e.g. activation of MAPKK activity, GO enrichment FDR= 3.83E-18). Therefore we believe that our analysis framework was not only suitable for ASD, but will also be useful for studying other biological pathways and human diseases as well.

We did not employ any specific threshold to define enrichment. In general we performed the regular hypergeometric tests for each module followed by multiple hypothesis correction (FDRs), and modules associated with FDR≤0.05 were considered significant. One additional criterion was used when performing the GO enrichment test, where we required that a module should have at least 5 member genes. As shown in Supplementary Fig. S4, this threshold was found to be the optimal for a trade-off in sensitivity and specificity.

3- The transcriptomic evidence presented in Figure 3 and page 10 is not as clear cut as it seems from the text with regard to general or tissue-specific expression. In both groups of genes, the heat map sections are very heterogeneous, and there are in both cases quite a number of individual genes that do not follow the general pattern of their group. In that respect, the overemphasis of the trends is not sufficiently supported by the experimental evidence and should be either discussed more precisely or toned down.

Our response: We thank the reviewer for this comment. In response:
(1) We now mention the issue of heterogeneity in the text. See below (pg. 12 in text) -

> *"Hierarchical clustering of normalized gene expression across brain sections revealed two distinct spatial patterns with some heterogeneity apparent in each…"*

(2) The clustering of Group 1 and Group 2 genes was based on several types of evidence. The heatmap was from a hierarchical clustering of the relative expression of these modular genes across 295 brain sections, which identified two gene groups (Group 1 and 2) that separated the neuronal brain sections (120 T2 regions, exemplified by the hippocampus in Fig. 3A) from the non-neuronal brain sections (175 T1 regions, exemplified by the corpus callosum in Fig. 3A). Such a natural clustering was further validated in Fig. 4B, where the grouping of Group 1 and 2 genes in this brain-section heatmap (Fig. 3A) was due to their preferential expression in neuronal cells and glial cells, respectively (P=6.4e-4, Chi-square test). This additional evidence assured that the clustering in Fig. 3A was solid and biologically reasonable.

(3). Despite the overall trend, as pointed out by the reviewer, individual genes in each gene group did not always follow this pattern. For example, in Fig. 3A, although the overall expression of Group 1 genes in the neuronal T2 sections was low (the blue pixels), a few genes showed increased expression marked by the red pixels. In our experience, this is expected from a regular clustering analysis of microarray data. To further validate our observations (from Fig. 3A), we RNA-sequenced postmortem brain tissues (Fig. 3B, with different technique and tissue sources from Fig. 3A). RNA-Seq supported our observations from the microarray data (Fig. 3A): Group 1 genes were highly enriched for the non-neuronal sections, represented by the corpus callosum (P<1.6e-6, Wilcoxon ranksum test), and Group 2 genes were most enriched for the neuronal brains sections, represented by the regions of BA9, 40 and amygdala (AMY, P<8e-7, Wilcoxon ranksum test). Please also note that not every gene in a cluster should act exactly like all genes in the cluster as genes can have multiple functions. Nonetheless, the strong enrichments and multiple lines of evidence support our conclusions.

4- Similarly, the evidence presented in Figure 4B and discussed on page 11 only points to overexpression of subsets of genes in oligodendrocytes, while failing to discuss large groups of genes that are overexpressed in neurons (bottom left corner of 4B) or in astrocytes (middle-right of 4B) and underexpressed in oligodendrocytes. The authors should discuss these groups and explain the rationale for not taking them into account as seriously as the others as possible contributors to the mechanisms of autism-related disorders.

Our response: We thank the reviewer for this helpful comment. In this manuscript, we specifically discussed the oligodendrocytes because this cell type is the major constituent of the corpus callosum in the human brain (as shown in Fig. 4A). We agree with the reviewer that genes highly expressed in neurons (which is expected, e.g. the Group 2 genes), and also in astrocytes, may also significantly contribute to this disease. Thus, following the reviewer's suggestion, we now point out that ASD might not be caused simply from a particular cell type. See below (pg. 20 and 21) -

> *"Group 2 genes, in addition their relatively high expression in the corpus callosum (**Fig. 3C**), showed the strongest expression in neuronal regions in brain (**Fig. 3B** and **4B**), explaining the high enrichment signal of synaptic genes in module #13 in our initial GO enrichment analysis. This observation supports the synaptic theory of this disease ... Since current ASD research has been primarily focused on neuronal regions, future study is warranted to examine the implications of other cell types in this disease… "*

5- The authors mentioned in passing two genes that appear as outliers in Fig. 5 D (DYNLL1 and BCAS1) with regard to their expression patterns. They should discuss whether or not this has functional implications.

Our response: (1) We labeled these two genes as outliers because of their extremely high expression in the corpus callosum (average FPKM>130 in the six individuals for both genes). In the correlation analysis, the two outlier genes (DYNLL1 and BCAS1) were included together with all other genes. **(2).** Molecular functions of these genes in the corpus callosum have not yet been reported in literature (to the best of our knowledge). Thus following this reviewer's suggestion, we asked whether their extreme expression could be also observed in other brain regions (especially in the neuronal sections). We re-examined both gene's expression in BA9, BA40, and the amygdala from one individual (ID:#5407, Fig. 3B) from our RNA-seq data, and their FPKM's are listed below in Table R1.

Table R1. DYNLL1 and BCAS1 expression (FPKM) in 3 neuronal regions from an individual #5407

	BA9	BA40	amygdala
DYNLL1	148.33	96.39	43.19
BCAS1	5.45	13.21	20.80

As observed from Table R1, it is clear that the high expression of DYNLL1 in the corpus callosum can also be seen in other neuronal tissues (BA9 and BA40), suggesting that its strong expression is likely ubiquitous across brain. In fact, this is consistent with its molecular function as an important cytoplasmic dynein complex subunit for cellular transportation. However, BCAS1, previously known as an oncogene candidate, exhibited substantial less expression in the 3 neuronal brain regions (Table R1) relative to the corpus callosum (FPKM=138.92) in this individual.

To determine that the expression enrichment of BCAS1 in the corpus callosum was not only limited to the individual in our sequencing study, we further examined BCAS1 expression from the Allen Brain Atlas (different individuals), and its brain expression is shown in Fig. R1. The microarray data were consistent with our RNA-Seq data, where the strongest enrichment of this gene in brain was in the corpus callosum.

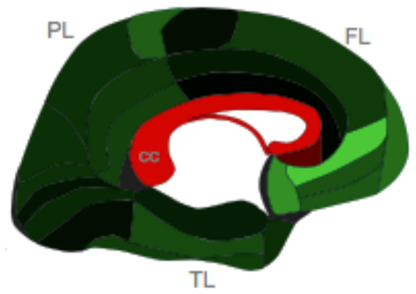

Fig. R1. Expression specificity of BCAS1 in the corpus callosum (CC). Z-scores of the microarray measurements of BCAS1 in each brain section were colorized from red to green indicating BCAS1 expression specificity in brain sections from high to low. FL, PL, TL, and CC represent frontal, parietal and temporal cortex and the corpus callosum, respectively. The data and image for BCAS1 were retrieved from Allen Brain Atlas.

Collectively, these comparisons suggest a novel function of BCAS1 in this particular brain region, and its molecular mechanism requires future exploration. We thank the reviewer for this helpful comment, and in our manuscript, we now further highlighted these 2 genes as follows (pg. 17):

> *"We note that two genes, DYNLL1 and BCAS1, displayed extreme expression in the corpus callosum (**Fig. 5D**) with FPKMs>130. Examination of their expression in the three neuronal regions (BA9, BA40 and AMY, **Fig. 3B**) revealed that DYNLL1 is a ubiquitously expressed gene with high expression across all the brain sections, whereas the extreme*

> *expression of BACS1 was unique only in the corpus callosum (FPKM<20 in other neuronal regions). Its specific expression in the corpus callosum was further confirmed on the microarray data from Allen Brain Atlas, suggesting a novel function of this gene in the corpus callosum. "*

6- The authors have put a great deal of emphasis on the potential role of the LRP2 gene and protein overexpression as an important component of module #13 implication in the patophysiology of ASD. Yet, it does not appear in the list of genes showing extreme expression differences in the matched case-control validation experiments reported in Table S6. This could mean that LRP2 is less important and/or that other genes/proteins listed are also important contributors, and this should be properly discussed by the authors.

Our response: This is a good point. (**1**) We and others (Ionita-Lazaemail, et al. 2012, Am J Hum Genet) have identified this gene showing excessive mutations in ASD patients relative to the control subjects, and its role in this disease is thus supported by replicable mutational analysis. However, our expression analysis did not reveal its abnormal expression among patients. This phenomenon has been observed for other diseases, where, for example, genes showing excessive cancer-associated mutations do not always exhibit differential expression between cases and controls. In particular, in colorectal cancers (CRCs), the KRAS mutant CRCs displayed distinct expression profiles from the wild type CRC samples, but the presence of the clinical KRAS mutations did not significantly shift its own expression (KRAS) in the patient cohort (Watanabe, T. et al. Eur J Cancer, 2011). This is not uncommon because an alteration of an amino acid may not necessarily lead to differential expression at the messenger RNA level of the same gene. (**2**) On the other hand, changing gene expression is not associated with deleterious nonsynonymous mutations either, which is evidenced by the fact that many of the differentially expressed genes in ASD patients (Voineagu, I. et al. *Nature*, 2011) did not harbor known pathogenic ASD mutations. (**3**) Thus we feel that a better practice is to integrate genomic data from different sources, rather than using one dataset to disprove the other. This is exactly the motivation of this study, where we integrated datasets from different sources to study the disease. We feel that the reviewer indeed raised a good point, which is an important angle to highlight the importance of data integration for the analysis of complex human disease. Upon the Reviewer's suggestion, we now highlighted these points in the first paragraph of Discussion (pg. 18-19 in text) –

> *"Most of our knowledge today about ASD genetics has been gained from genetic association or exome-sequencing analyses of large ASD patient cohorts, which allows us to begin to observe the molecular underpinnings of this disease. However, a complete picture for this disease may require an integration of ASD genetic data from different dimensions. For example, a number of studies have analyzed genes that displayed differential expression in ASD brains (Voineagu et al, 2011), but aberrant mutations have not yet been identified for most of these genes. Since the retention of genetic mutations within a population is strongly driven by natural selection and population demographics(Hartl & Clark, 2007), mutations in genes critical for ASD are likely to depleted by purifying selection or simply by population bottleneck, preventing the identification of ASD candidate genes only from mutational analyses. In addition, another example of a gene that would be missed by differential expression studies is LRP2, whose implication in ASD was found by the sequencing studies in this study and an earlier investigation (Ionita-Laza et al, 2012); but it did not exhibit altered expression in ASD patients. These observations strongly suggest that genetic alterations leading to ASD might occur at different levels, perturbing gene regulation or affecting gene function, and highlight the importance of building an integrative model to study ASD, where genomic data from multiple independent dimensions are incorporated to reveal the hidden architecture of this disease. "*

(**4**) We also wish to note that we mentioned LRP2 in this paper was because this protein is specific in the corpus callosum, and had excessive mutations in our screen. However, the majority of our story is centered on the entire module #13, where we examined brain/cell type expression of all of its members and determined expression dynamics of the gene sub-groups within this module, not just limited to LRP2. We agree with the reviewer that except for LRP2, many other genes are also likely important contributors to this disease, and thus in this revision we provided new data to show the mouse mutant phenotypes of genes in this module (pg. 10), which demonstrates the importance of other modular genes (not only LRP2) in this disease.

> *"…To better support their association with this disease, we further examined their mouse mutant phenotypes in Mouse Genome Informatics (http:// http://www.informatics.jax.org), and observed that 10 of the 28 new candidate genes displayed abnormal behavioral traits or a defective nervous system in their respective mouse mutants (see Supporting File S3). For example, mouse mutants of 1) ANKS1B and KCNJ12 exhibited hyperactivity, 2) ERBB2IP hyporesponsive behavior to stimuli, 3) GRID2IP abnormal reflex and 4) SCN5A seizure…"*

7- The manuscript is overall well written and clearly structured, although in some sections rather lenghty and technical. Although this is largely unavoidable due to the complexity of the approach and the requirements for careful assessments of confounding factors, the manuscript could benefit from a comprehensive and more concise presentation, associated with careful English syntax editing.

Our response: We thank the reviewer for this comment, and we now have carefully revised the manuscript to make it more compact and concise. We found the reviewer's comments to have been very helpful in improving the manuscript, and we sincerely thank this reviewer for all these insightful suggestions.

Reviewer #2:

1. Summary: In this very extensive and well-done study, Li and colleagues construct an interaction network from human protein interaction data. They find and validate an autism-associated module with gene expression and genome sequencing data. They delve into the biology of this module and find enrichment for oligodendrocyte and corpus callosum expressed genes, implicating these cells and this neuroanatomical region in autism pathogenesis.
More specifically, Li and colleagues look at the BioGRID human protein interactome data as a network, and verify the quality of the network by evaluating its agreement with other data, including RNA co-expression data. They apply a topological clustering analysis to identify 817 modules, and global modularity in the observed interaction network is convincingly demonstrated with an edge-shuffling approach that retains global topological relationships. 85 of the identified modules show enrichment for a previously annotated gene ontology terms. Out of these modules, one (Module #13, 119 genes) was enriched for known ASD genes taken from the SFARI database. This enrichment was robust to permutations controlling for GC content and gene length. Intriguingly, Module #2, which was related to DNA-dependent transcription, also showed enrichment for ASD genes.
The authors then extensively validate Module #13's relationship to autism. They show that the enrichment for Module #13 was strong and not entirely driven by trivial factors such as gene list bias or synaptic genes and demonstrate strong enrichment from high-confidence CNV-affected genes and weaker enrichment for larger CNV and SNV gene sets. The authors also show that Module #13 is enriched for autism genes more than schizophrenia genes, and not enriched at all for Alzheimer's disease genes. These analyses show specificity of the module toward autism-related genes from multiple sources. Taken together, the authors convincingly identify an autism-relevant module in the available human protein interactome.
The authors next use data from 25 sequenced autism patients, and find 113 nonsynonymous mutations in Module #13 to be weakly enriched over the exome background at $p = 0.02$. They show that the preference of these mutations for Module #13 was not strongly affected by gene length. Based on recurrently observed mutations in LRP2, an initial lead to the corpus callosum is suggested. This is followed up on later. Additional comparisons to larger patient cohorts demonstrated that 38 module-specific candidate genes were enriched for mutations, though at moderate statistical significance (uncorrected $p < 0.05$, but $p > 0.01$ in many cases).
The rest of the manuscript follows the LRP2 lead and attempts to dissect the cellular and neuroanatomical relationships implicated by Module #13. First, it is shown that Module #13 can be split into two groups co-expressed across human brain regions using the Allen Brain data, with one more ubiquitously expressed (group 2), and the other expressed most highly in corpus callosum (group 1). Next, this enrichment for corpus callosum is validated by the authors' own RNA sequencing and further confirmed by immunohistochemistry to confirm LRP2 and oligodendrocyte

© European Molecular Biology Organization

presence in the corpus callosum. Next, to home in on cell-types, the authors use mouse cell-type data to implicate oligodendrocytes as affected by group 1 and group 2 and combine mouse oligodendrocytes culture and MRF knockout data to suggest that the up-regulation of group 1 and down regulation of group 2 is essential to proper oligodendrocytes development. Finally, RNA-seq from the corpus callosum of ASD patients is used to show that, taking the union of dysregulated genes across patients, Module #13 genes are more affected compared to synaptic genes or broader autism genes. Most of these enrichments were marginal in effect size at the gene set level, but enough data is provided to put forth a compelling story.

General remarks: It seems that authors are trying to drive home three major points: 1) the protein interactome in humans is modular, and that this structure can be used to better understand autism, 2) a protein-interaction module could help prioritize mutations from whole exome and genome studies, and 3) protein-interactions implicate oligodendrocyte maturation and corpus callosum development in autism. Extensive validation is performed, which provides further experimental evidence supporting these three major points. This study therefore will be of widespread interest.

Our response: We sincerely thank this reviewer for his/her appreciation of our effort and these valuable comments, which are very helpful to improving our work. Below we provide more details for the reviewer's questions, and also the corresponding changes in our text following the reviewer's suggestions.

2. However, the claims of novelty or uniqueness as put forth by the authors are less convincing and one wonders why this elegant work is not more integrated with other published work. A simple literature search reveals multiple studies that have identified interaction networks of various sorts implicating specific neurodevelopmental pathways in autism (Ben-David and Shifman, 2012b; Gilman et al., 2011; Parikshak et al., 2013; Willsey et al., 2013), and several have used protein interaction networks in a variety of ways (Corominas et al., 2014; Cristino et al., 2014; Sakai et al., 2011), many of which are not cited. The value of this study is not for its "novel" use of genome wide interaction data per se. Rather, the study does employ a unique combination of previously used approaches in a manner that is of very high quality and that is more convincing than some of the previous studies and is quite complementary to others - this latter point needs more emphasis. It also delves deeply into the biology of one of the network components, and arrives at a convergent pathway in autism.

Our response: We fully agree with the reviewer that the novelty of this work lies in how we analyzed the 'omics' data to gain new insights into this disease, rather than in the use of protein interaction dataset. The 4 papers listed above that we had not mentioned (Corominas et al., 2014; Cristino et al., 2014, Parikshak et al., 2013; and Willsey et al., 2013) were published well after we first completed our study. In fact, the 2014 papers were published almost at the same time or even after our submission to MSB. Nonetheless, we have substantially re-written our Introduction and Discussion section, and included all these papers listed by the reviewer. We thank the reviewer for this thoughtful suggestion.

3. But one is left asking: What about ASD that has been observed in these previous studies can this work confirm or validate, and what does it not validate? This work will be most interesting for neuroscientists and geneticists studying neurodevelopmental disorders, particularly autism. For this audience, it would be ideal for the authors to clearly demonstrate how the module identified here relates to the existing work described above. This is discussed in detail in the major points below. There are also technical concerns that should be addressed to give readers more confidence in the soundness of the work.

Our response: This is an excellent point. In this revision, we now followed the reviewer's suggestion and compared more extensively our findings with the conclusions from other ASD genomic studies. As discussed in the later sections, most of our observations were not redundant from previous studies, and has provided additional insights into this disease. These comparisons now appear in the Introduction and Discussion sections in the revised manuscript.

Major points:

4. - Grammar needs some checking - there are problems with articles and plurals throughout the paper that detract attention, starting in the Introduction.

Our response: We thank the reviewer for this comment, and now we have carefully edited the manuscript, and have fixed the grammatical problems.

5. - The major novelty of this study as described in the manuscript originates, as the authors state, from identifying a "naturally occurring pathway underlying this disease" instead of starting from mutations as has been done in other studies cited: (O'Roak et al., 2012; Willsey et al., 2013).
o However, others have used "naturally occurring" pathways defined at the genome-wide transcript co-expression level to do similar types of analyses (Ben-David and Shifman, 2012b; Parikshak et al., 2013)
o They have also been identified using a "seeding-and-expansion" approach at the protein interaction level (Cristino et al., 2014), which also uses a clustering algorithm to identify shared relationships (though the work here is more genome-wide and considerably more statistically sound, again a comparison that can be discussed by the authors)

Our response: We thank the reviewer for this comment. As we mentioned above, now we have substantially re-written our Introduction and Discussion sections to discuss these papers (many of which were published only very recently, after the completion of this project) and to give a better description of the novelty of our study as suggested by the reviewer. We now compared our results with other papers in the Discussion section.

6. - The novelty in this study came across to this referee from the following:
o Implication of the corpus callosum, however note that
• Previously, Ben-David et al. 2012 identified corpus callosum modules using the same anatomical expression data, and a connection to immature oligodendrocytes was also made. They had a "black" and "yellow" co-expression module that were both highly expressed in this region, but they did not find strong evidence that these were affected by the autism risk genes they assessed. • The authors should at least cite that study and ideally compare Module #13 to these two modules.

Our response: We thank the reviewer for this comment. We now examined their "black" and "yellow" modules (59 and 744 genes, respectively), and found that the overlap between our protein interaction module #13 with their two co-expression modules were minimal, with only one gene (PGM5) also in their "black" module and one gene (GRID2) in their yellow module. This difference probably explains why the ASD candidate genes were not enriched in their co-expression modules highly expressed in the corpus callosum, and also suggests that our analysis with completely different approaches (topological clustering rather than their co-expression analysis) has indeed provided new insight into this disease. We thank the reviewer for this interesting comparison, and now we have cited this paper in our manuscript. In our revised manuscript, we now have mentioned this comparison in our Discussion (pg. 22) –

> *"Two groups of genes were identified previously which displayed elevated expression in the corpus callosum, but were not significantly associated with ASD (Ben-David & Shifman, 2012). The overlap between our module and these genes was restricted to two genes. "*

7. • Parikshak et al. also describe enrichment for ASD genes (based on RNA expression) in neurons that project intra and interhemispherically in layers 2/3 of monkey cortex that comprise virtually all of the collosal projecting fibers in primates. This finding therefore would be consistent with the current analyses presented here and this should be emphasized as it uses a very different approach to come to a similar condition. This is also consistent with a model of cortical disconnection, which is the first such study since Parikshak, to show this. Furthermore, the data here are somewhat stronger in this regard, so these findings could be considered a significant extension of this notion.

Our response: We thank the reviewer for this comment. We had a small paragraph in an earlier version of the Discussion that points out the connection of our study with Parikshak et al. We now systematically compare our conclusion with those derived from earlier genomic studies, and highlight that the model of "cortical disconnection" supported by Parikshak et al. is also supported by our study with a very different approach. See below (pg.21) –

© European Molecular Biology Organization

"Two recent studies (Parikshak et al, 2013; Willsey et al, 2013) have implicated the superficial cortical layer (II/III) or the deep cortical regions (layer V/VI) in ASD. Callosal projection neurons are primarily localized in the superficial layers II/III (~80%) or deep layers V/VI (~20%); thus our study now connected the two studies suggesting a critical role of the interhemispheric connectivity circuitry, whereby disrupting its sub-components to affect the interhemispheric signal transduction through the corpus callosum will likely to give rise to ASD phenotypes. Therefore the disease etiology should be understood at the level of the complete interhemispheric connectivity circuitry, not simply by a particular brain region or cell type. This could not only explain the enrichment in ASD-associated mutations in genes highly expressed in the constitutive parts of the circuitry (superficial or deep cortical layers in the earlier studies, or in the corpus callosum in this study), but also might provide a molecular basis for the observation from the imaging studies of the under-development of the corpus callosum among ASD patients. Importantly, different from previous research, our study illustrates the role of the oligodendrocyte cells in ASD, which myelinate and support the axons in the corpus callosum for interhemispheric signal transduction. Since current ASD research has been primarily focused on neuronal regions, future study is warranted to examine the implications of other cell types in this disease."

8 • The RNA profiling in corpus callosum is the first such study and provides strong experimental support. The enrichment analysis with control gene sets is elegant.
o Usage of new genomic variant data to validate the network
• Recent work has done this with the above-mentioned protein interaction based "seeding-and-expansion" approach (called AXAS) with exome sequencing in trios, suggesting An et al. 2014 could group ASD-associated mutations in families - the current work should discuss how it differs or agrees with the An et al. work (An et al., 2014)
o I think the authors have made greater advances in both of these areas relative to the above two studies, and they should emphasize the strength of their work in that context rather than only its novelty. In reality the strength of this study is that it is very well done and has solid statistics and assumptions and validation.

Our response: We thank the reviewer for this suggestion, and we now provide our comparison with An et al. We first compared the technical differences between our study and AXAS used in An et al, which was initially proposed by Cristino, A.S., 2014, and then we show how the differences has led to different observations between our study and the exome-sequencing study by An et al. (also Cristino, A.S., 2014).

(1). Their AXAS network was defined based on the "seeding-and-expansion" approach. 534 genes associated with autism, X-linked intellectual disability, attention deficit hyperactive disorder and schizophrenia were sampled from the global human protein interaction network, and the authors made the AXAS network by linking these genes with their first-degree interacting neighbors on the network. Then all the downstream analyses, such as the exome-seq analysis by An et al., were limited to this seeded-and-expanded sub-network.

In contrast, our approach did not use the "seeding" strategy, but started with the global network to identify a natural module associated with ASD, followed by more detailed functional characterizations of the identified ASD module. In this regard, their AXAS was an empirical framework (ascertained by previous association studies), but our module reflects the natural organization of gene-gene interactions (previous ASD data merely served the purpose for validation, but not for identification).

(2). The AXAS network included 2743 genes in their empirical ASD network (the seeded ASD genes with the first-order interacting proteins), but did non capture ~30% of the modular genes (119 genes in total) in our analysis. In the publication by Cristino et al., clustering was performed on their AXAS network, but it was only for examining the distribution of genes from different disease categories (ASD, ADHD, SZ, etc.) in each AXAS cluster. They reported one AXAS cluster (680 genes) enriched for synaptic function; however their "seed-and-expansion" was based on previously curated ASD genes, which themselves are enriched for synaptic transmission.

(3). An et al. used the AXAS network and reported on 1754 genes that were regarded as 'causal' genes for ASD based on their patient data (Supplementary Table S4 in An et al., where these genes were listed in the 1st column labeled as "*causal variants in cases*"). We observed 19/1754 genes that were included in our module (119 genes). The degree of overlap was not statistically significant as the ratio was also expected from a random draw from the genome (P=0.11, Fisher's exact test).

Taken together our analytical approach differs substantially. Unlike An et al., where AXAS were used to prioritize ASD candidate genes, our mutational analyses (together with other molecular data) were primarily employed to characterize the molecular function of this module, and to establish its functional association with ASD. In this regard, we agree that mutations on other network components outside of our module may also contribute to this disease.

In the revised manuscript, we now compared with our results with AXAS in our Discussion. See below (pg.22) –

> *"Notably a more recent paper considered a sub-network implicated in ASD constituted by known ASD candidate genes and their first-degree interacting neighbors (An et al, 2014; Cristino et al, 2014). This empirical network was large and encompassed more than 2000 genes for ASD, but ~30% of genes in our module were not captured by their empirical network."*

9 - The authors control for potential biases related to gene length and GC content as done in previous work (O'Roak et al., 2012; Willsey et al., 2013). However there is substantial additional biases to consider when using global but incomplete protein interactome data that are curated from multiple studies and whole exome / whole genome data for enrichment analyses:
o Protein interaction networks in human are incomplete. Other than the fact that they are not tissue or time specific, it is estimated that there are 150K to 370K total interactions in the human proteome Hart et al., 2006), and only 69113 could be assessed here (ignoring inaccuracies in the interaction data). Therefore, only 20-50% of the putative human interactome is measured. Furthermore, even in yeast, even when ~50% of the interactome was measured, a study showed that the global topological structure of the interactome is highly unstable, depending on what filtering steps are taken to include protein interactions and the fact that the most studied genes have the most interactions (Hakes et al., 2008). It is likely that these issues highly affect the future generalizability of the networks presented here, particularly the high modularity (>800 modules) observed. Here are two suggestions to be more transparent about this bias:

Our response: We thank the reviewer for this comment. The two suggestions are addressed separately below (see our response to the point #10 and #11), and we herein briefly answer the question about using the human protein interaction network. First of all, we agree with the reviewer that current protein interactome data are incomplete; however even the incomplete network has substantially advanced our knowledge about human diseases. For example, in a very recent paper in *Science* for corticospinal motor neuron disease, the investigators mapped identified mutations from exome-sequencing to the similar curated protein interaction network ("*a protein network of all known human genes and/or proteins*", quoted from the paper), and successfully identified the disease-related pathways (Novarino, G. *et al., Science* vol. 343:*506-511, 2014*). In addition, earlier work showed that using the same type of curated protein interactions can successfully predict breast cancer prognosis (Taylor, I.W., *et al. Nature Biotechnology*, 27(2): *199-204*). A recent study also showed that disease mutations are more likely to disrupt the known protein interactions curated from all sources like BioGrid (Wang, X. *et al. Nature Biotechnology*, 30(2):*159-64*). Importantly, in the ASD community, these protein interactions have been increasingly used (e.g. the InWeb network examined in the *Nature* papers for ASD exome-sequencing for *de novo* mutations, Sanders, S.J. *et al., Nature*, 2012, and O'Roak, B.J. et al., *Nature*, 2012, and also the paper by An et al. for the AXAS network as we discussed above). We share the same concern with the reviewer, and we performed additional quality controls for the curated protein interactions used in this study:

(1) We have compared the data quality against the best benchmarked human protein interaction data (Wang, X. *et al. Nature Biotechnology*, 30(2):*159-64*), and we showed that the interactome data used in this study showed higher gene co-expression, an important criterion for assessing the data quality from yeast-two-hybrid or APMS studies (affinity purification followed by mass spectrometry).

(2) We also considered the issue raised by the reviewer that the highly studied proteins may have more interactions. In fact, we examined this issue, and counted the number of literature curated interactions that support the interactions in our ASD module, and compared these with that from the global network background, but did not see a substantial difference. The results of this comparison are presented in our Supplementary Information.

(3) As noted by the reviewer, the network structure might be unstable; thus in our study, we have taken this into account (also see our response to the next point for the work by Rossin et al., 2011). Since the detection of network modules was purely based on network topology we used degree-preserving shuffling to simulate the white noise in the network. This permutation was performed by generating 100 such pseudo-networks with the same size of the real network (we did not go above 100 because this simulation was very computationally expensive). It is important to note that for network topological analysis, this protocol is the standard protocol for highest stringency and was previously proposed and used in detecting network motifs (Milo, R., *Science*, 298(5594):*824-827*). With this analysis we showed that our module detection is highly robust against random fluctuations (Supplementary Fig. S3).

(4) Most importantly, on the biological side, we performed RNA-seq in the corpus callosum, and we can clearly see a significant correlation between network topology and mRNA abundance in this specific brain region (Fig. 4C). This strongly suggests that even if we do not have the complete interactome data, the curated interactions from all sources obtained thus far could indeed provide useful insights.

Overall we thank the reviewer for asking this question, and with all these discussed above, we hope the reviewer will appreciate our effort in performing all these control experiments.

10 • The authors could try to model the effect of bias in their permutation analyses. See Rossin et al., 2011 for details (Rossin et al., 2011). They could enact a better permutation scheme by including, in addition to GC content and CDS length (or in lieu of these factors), genes that have a similar distribution in the global number of protein interactions in the global network. If Module #13 is not driven by biases in the overall interactome, then the calculated p-values should remain significant.

Our response: We thank the reviewer for this suggestion. The paper by Rossin et al., 2011 generated a set of randomized networks with the same topology as the real network by randomly re-assigning gene labels to other network nodes of the same degree – such a random re-assignment can exactly be thought of as shuffling node edges to other interacting protein but maintaining the same degree for each node (to simulate non-specific bindings), which was exactly the same protocol as we used in this study (and also in Milo, R., *Science*, 298(5594):*824-827*, which was used to detect network motifs). Thus this shuffling protocol generates a set of randomized network with the same size, edges and degree distribution as those in the real network. With this protocol, as shown in Supplementary Fig. S3, none of the permutated network could achieve the modularity seen from the real network. In fact, our human genomic data and mouse data strongly support the notion, that module 13 as a whole clearly plays an important role in the corpus callosum (Fig. 4), thus excluding the possibility that this module was merely a chance finding.

Since module detection was purely a topological analysis (and the same is true for network motif detection as in Milo, R. et al.), we feel it is better to keep those modules as “purely topological” clusters, i.e. not affected by non-topological elements. The reason we considered unequal CDS and GC content was because they may generate unequal number of mutations, which, is separate from defining network modules. Thus it is more reasonable to only include these factors in the downstream mutational analysis.

11 • The authors could use recent work deeply querying a subset of the interactome (Corominas et al., 2014), which likely overlaps with Module #13. These interactions could be used to estimate how biased the current network structure might be, and how complete it is relative to a known "complete" subset.

Our response: We thank the reviewer for this suggestion. (**1**).We extracted the reported 506 ASIN gene-level PPIs from this Y2H study (Supplementary Data 4 from Corominas et al., 2014), which included 71 unique BD proteins and 291 unique AD proteins. Among the 71 bait proteins, only 3 (DLGAP2, INPP1 and NLGN3) were in our module #13. Thus, it is difficult to determine biases from only 3 proteins. (**2**). Module #13 detection was dependent on the connections within and outside of the modules (i.e. connection with the background network), so we feel it would be difficult to just use the 506 Y2H interactions to assess the overall quality of our module detection or the completeness of the global network. In fact, careful examination also revealed that many interactions not seen from this Y2H could also be detected by APMS in vivo, or by other small-scale biochemistry studies. For example, this Y2H did not report a positive interaction between FMR1 and FXR1; however, this interaction has been well known as early as 1995 (Zhang, Y. et al., EMBO J. Nov. 01, 1995; 14(21);5358-66). Undoubtedly, this Y2H resource is very valuable for the ASD community, but due to its limited size, a comparison with these 506 Y2H interactions might not provide confirmatory evidence to prove or disprove protein interactions from other sources at this stage. In our discussion, we now have cited the Y2H papers (pg. 22):

> *"Worthy of note, based on independent yeast-two-hybrid screens, recent studies have attempted to generate the complete interactomes for individual proteins implicated in ASD (Corominas et al, 2014; Sakai et al, 2011), and thus we envision a significant expansion of our current observation when the human protein interactome is more complete."*

12 o The authors controlled for GC content and CDS length in enrichment for the initial SFARI gene list in Module #13 by permuting random sets matched for these factors.
• However, they seem to not use this permutation scheme for some of the later enrichments where these factors are actually playing the greatest biasing role - with the genetic variants from exome and whole genome studies. The GC content/CDS length permutation approach should be used throughout the study, rather than relying on constructing "matched" backgrounds or doing Wilcoxon tests for contributions from gene length - these are less stringent approaches and it becomes convoluted when different approaches are used at different points in the manuscript.

Our response: In this revision, we have re-analyzed these sequencing data to include the GC content/CDS length permutation approach as suggested by this reviewer. Briefly, we mapped the non-synonymous variants identified from the patients onto module #13. Among the total of 153 non-synonymous variants in the module, we observed 30 that were rare and not previously observed from the 1000 Genomes dataset. We then performed 10000 random permutations by randomly sampling the same number of genes (as those in this module), and these 10000 sets of genes in our permutation test were all with indistinguishable CDS length (P=0.1008, Wilcoxon rank-sum test) and GC content (P=0.82, Wilcoxon rank-sum test) from the genes in this module. With these permutated gene sets (Fig. R2), we performed the same analysis, and found the enrichment of the rare variants in this module was not expected by chance (P=1.2e-3).

© European Molecular Biology Organization

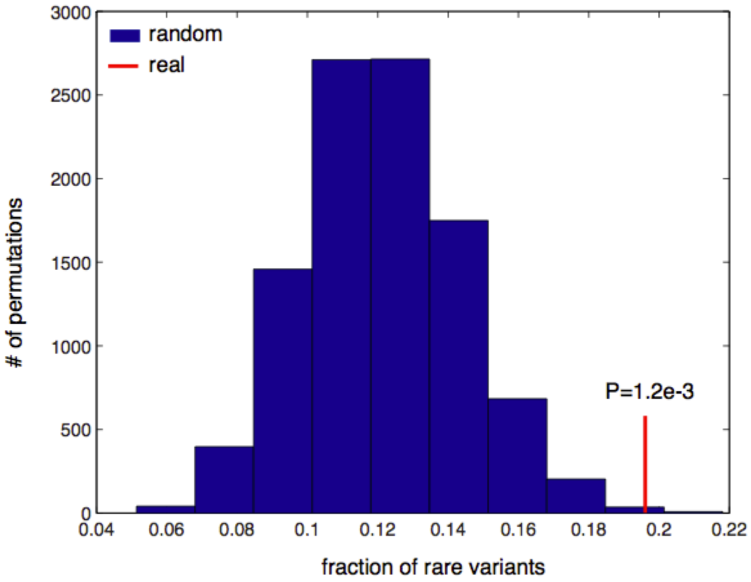

Fig. R2. The enrichment of rare nonsynonymous variants in this module was not expected by chance. The histogram was derived from the 10000 random permutation test.

We thank this reviewer for this helpful suggestion, which now provided a more stringent comparison. We have now included this permutation test in the revised manuscript (pg. 9 in text).

> *"…identified 153 non-synonymous variants that were mapped onto the module, among which 19.6% (30/153) were extremely rare and were not previously observed in the 1000 Genome dataset. Randomly sampling the same number of genes 10,000 times, with indistinguishable CDS length and GC content from those in this module, demonstrated a significant enrichment for the rare non-synonymous variants in this module (P=1.2e-3, with the expected fraction 12%)."*

13. *The authors find enrichment for genes expressed in the oligodendrocytes and the corpus callosum as seen in previous work (Ben-David and Shifman, 2012b)*

Our response: As we discussed before, their "yellow" and "black" modules had minimal overlap with the one in our paper, and now we have cited this paper in our manuscript.

14 o A concern here is that the preference for the corpus callosum and oligodendrocytes over other regions shown in Figure 3 and 4 is marginal. The authors should discuss the reason why group 1 specificity for the corpus callosum and oligodendrocytes is significant, but not very striking, and discuss what other cells and regions might also be involved.

Our response: We apologize for not making this point clear. (**1**). From the microarray data (Fig. 3A in the manuscript, from the Allen Brain Atlas) our hierarchical clustering showed that the 119 genes in this module had a clear separation of the two sub-components defined by their expression preferences in different brain sections, and the Group 1 genes was one of the components showing increased expression in the 175 T1 regions relative to the other 120 T2 regions (Fig. 3A in the manuscript); the corpus callosum was a prominent example that was further validated by our RNA-Seq analysis in this manuscript (Fig. 3B in the manuscript, also see Fig. R2 below for more information).

As we discussed in the manuscript, this clustering pattern was mostly driven by the cell type composition in different brain sections, and especially for the Group 1 genes showing tissue enrichment for the corpus callosum. At the cell type level, their expression (Group 1 genes) showed high enrichment for oligodendrocytes and astrocytes but was depleted for neurons (Fig. 4B in the manuscript). This explains why their (i.e. the Group 1 genes) enrichment in the 175 T1 brain sections from our hierarchical clustering, as shown in Fig. 3A, was mostly due to white matter

structures, and their expression was insignificant in the other 120 neuron-rich T2 sections. As confirmed by our IHC study (Fig. 4A in text), the major constituent of the corpus callosum was the oligodendrocytes, and thus the expression preference of Group 1 genes in this tissue was expected.

(**2**). We then tested this expression preference by RNA-sequencing postmortem brain regions for a healthy individual (Fig. 3B in the manuscript). To elaborate this comparison for the expression preference in the corpus callosum, now we re-examined the data in Fig. 3B, but only looked at the FPKM expression of Group 1 genes in the corpus callosum (CC) relative to other brain regions enriched for neurons (BA9, BA40, and AMY, the red boxes in Fig. R3). As shown in Fig. R3 below, it is clear that Group 1 genes displayed the strongest up-regulation in the corpus callosum (relative to the transcriptome background, P=1.6e-6, Wilcoxon ranksum test) than in the other neuronal regions (P=9.7e-3, 8.6e-3 and 1e-3 in BA9, BA40 and AMY, respectively). The RNA-Seq data supported our observations from the microarray data in Fig. 3A, and thus confirmed the expression preference of Group 1 genes in the corpus callosum, and thus the difference was not marginal.

(**3**). In our study we extensively studied the corpus callosum given that it gave the strongest signal for Group 1 genes (Fig. 3A and B in the manuscript). We combined these expression data with mouse knockout data and demonstrated their functional role in the oligodendrocytes in the corpus callosum. However, these genes may also have other important functions in other brain sections enriched for the oligodendrocytes and astrocytes given their cell-type-specific expression.

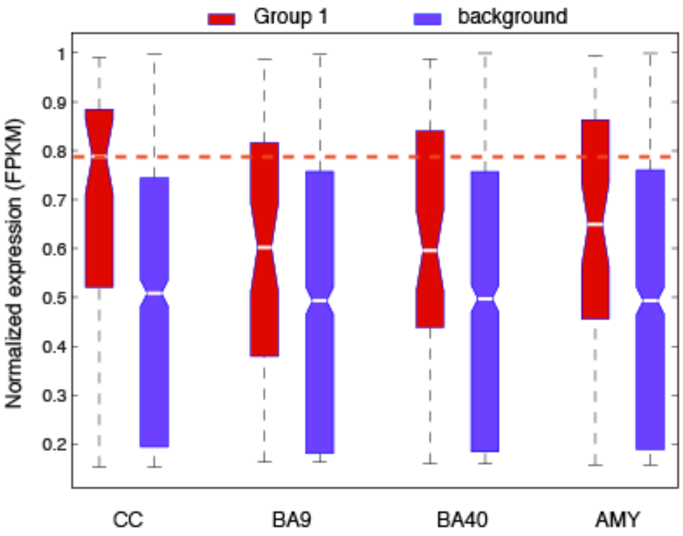

Fig. R3. The expression propensity of Group 1 genes in the corpus callosum over other brain regions. The expression was quantified by FPKM from our RNA-Sequencing experiments. The expression propensity in the corpus callosum (red boxes) were also tested against a group of genes randomly sampled from the transcriptome background in each brain region (blue boxes), and confirmed that this expression preference was specific for Group 1 genes.

15. The sequencing study in 25 patients identifies 38 candidate loci, mostly missense. Much larger exome sequencing studies have shown these classes of mutations to occur equally in probands and controls essentially, so the genetic evidence presented here is not convincing that they have detected 28 new (not described previously) genes. They would need to compare case-control burden doing a proper case-control study as is standard in human genetics. This is consistent with the modest increased burden in module 13. Is this corrected for multiple comparisons? The use of dbGAP samples helps bolster their claim. Perhaps focusing on the 14 genes from among the original 38 for which there is some additional support is more appropriate than their current emphasis.

Our response: We thank the reviewer for this comment. (**1**) We agree with the reviewer that common missense variants usually have equal distribution between cases and controls (Anney, R. et

al. 2012, Hum Mol Genet.); however the 38 genes identified from our sequencing study mostly harbor rare variants with average allele frequencies f≤0.01 in the 1000 Genomes dataset, and 2/3 of the variants were private among the patients and not observed among the comparison populations (1000 Genomes). We now provide additional evidence, which supports their involvement in ASD. (**2**) 10 genes among the 38 genes have been already annotated in SFARI For the remaining 28 genes we examined their corresponding mouse mutant phenotypes, and 10 among the 28 new genes displayed defective behavioral (neurological) traits or abnormal phenotypes in their nervous system according to MGI annotation (Table R2).

Table R2. Mouse mutant phenotypes for the newly identified candidate genes

Human Genes	Mouse Genes	Behavior/neurological	Nervous system
ANKS1B	Anks1b	X	
DLG1	Dlg1	X	X
ERBB2IP	Erbb2ip	X	X
GRID2IP	Grid2ip	X	X
GRIK3	Grik3		X
KCNJ12	Kcnj12	X	
KCNJ15	Kcnj15	X	
NOS1	Nos1	X	X
SCN5A	Scn5a	X	X
UTRN	Utrn		X

For example, the NOS1 mouse mutant displayed altered aggression response, increased grooming behavior and abnormal seizures and sleep patterns. ANKS1B and KCNJ12 led to hyperactivity, and ERBB2IP is associated with hypo-responsive behavior to stimuli. In particular, the ERBB2IP mouse mutant also displayed abnormal myelination and axon morphology, in concordance with our conclusion in later sections about the role of this module in regulating the myelination process in the corpus callosum (Fig. 4D). Only ~6000-8000 genes have available phenotype information at this stage, which is not yet complete. Thus the phenotypic associations support their involvement in this disease, and future study is warranted to study the phenotypes of other genes on the list. This new information is now provided in the revised manuscript (pg. 10, also see below):

> "*To better support their association with this disease, we further examined their mouse mutant phenotypes in Mouse Genome Informatics (http:// http://www.informatics.jax.org), and observed that 10 of the 28 new candidate genes displayed abnormal behavioral traits or a defective nervous system in their respective mouse mutants (see Supporting File S3). For example, mouse mutants of 1) ANKS1B and KCNJ12 exhibited hyperactivity, 2) ERBB2IP hyporesponsive behavior to stimuli, 3) GRID2IP abnormal reflex and 4) SCN5A seizure*."

Furthermore, these identified genes in our study also showed non-random distribution on the protein interaction network as revealed by our network analysis (Fig. 5B and C in the manuscript), and our analyses on the independent dbGAP cohort also showed these genes were more likely to have disparate allele frequencies between cases and controls. Thus we feel that it is reasonable to present the 38 genes from our analysis, which will likely motivate further study for their functions and potential implications in ASD. We agree with the reviewer that higher confidence will be achieved if a candidate locus is supported by multiple lines of evidence, so in the supplementary dataset, we now separately indicated the 14 genes also supported by the dbGAP dataset (Supporting File S3). With this, the audience will be able to make their own decisions on focusing on a particular set of genes for their future study.

(**2**) For the mutational burden test as suggested by the reviewer, we have now provided additional analysis to test the role of this module in ASD. Since a mutational burden test is ideally performed on a large patient cohort and CNVs are known to have greater effect size in ASD, we examined the new de novo CNV datasets from 2446 ASD-affected families (Pinto, et al. 2014, Am J Hum Genet) together with previous ASD-associated de novo CNV datasets from Sanders et al. and Levy et al. (2011, Neuron). Using both the union and intersection sets of CNV calls from multiple studies, we consistently observed the module was more enriched for these CNV-affected genes in ASD

© European Molecular Biology Organization

probands relative to the other genes with indistinguiable CDS length and GC content (19.3% vs 11.27%, P=0.012 for the union set, and 5.04% vs 2.07%, P=0.039 for the intersection set, Fisher's exact test). We also considered de novo CNV events from healthy individuals (Kirov, G. 2012, Mol. Psychiatry) or unaffected siblings in Sanders et al. and Levy et al., but did not observe any enrichment signal in the module (1.68% vs 2.65%, P=0.77). Therefore we concluded that the module has increased mutational load for de novo CNVs in ASD probands. We thank the reviewer for this insightful comments, and now in this revision, we have added a brief discussion on the associated mouse mutant phenotypes of our genes identified, and also included the mutational burden test for the de novo CNVs (Supplementary Table S1B, and pg.8 in the revised manuscript). Lastly, all the statistical significance was determined after multiple hypothesis correction, and false discovery rates (FDRs) were presented.

16. Currently the manuscript is not clearly placed in a quantitative context with the published systems level work that already exists in ASD, but this could be easily done. To help orient readers, I have a few additional suggestions (not necessary to address as module-level data may not easily be available from all of the studies - more important to address the data sharing minor point):
o Compare Module #13 with the results from the AXAS (Cristino et al., 2014) and NETBAG (Gilman et al., 2012; 2011) approaches that also heavily rely on protein interactions.

o Directly compare with more transcriptomically-driven modules from Ben-David et al (Ben-David and Shifman, 2012b), Parikshak et al (Parikshak et al., 2013), Willsey et al (Willsey et al., 2013), and/or Liu et al (Liu et al., 2014)

o Check the developmental time course of the identified module on BrainSpan - though may not be helpful since the BrainSpan database does not contain corpus callosum. Other studies have done this by simply taking normalized expression levels for the module and assessing plotting them for different regions (Ben-David and Shifman, 2012a; Gilman et al., 2012; Gulsuner et al., 2013).

Our response: We thank the reviewer for this comment, and as mentioned above, in the revised manuscript, we now have added one section in Discussion to compare our study with other related publications (pg. 21-22 in text). (**1**) The comparisons with Ben-David et al (Ben-David and Shifman, 2012b) and AXAS have been shown in our responses above (point #6 and #8), where their overlaps with our module were insignificant. (**2**) We now compared our module genes with those identified by NETBAG (Gilman et al., 2012), and again we only found 4 genes (among the 119 genes, DLG1-4, NLGN3) also identified NETBAG. We particularly note that NETBAG was based on a network constructed for gene pairs sharing any potential functional association, including shared interacting partners, co-expression, co-evolution, shared GO annotation, similar KEGG annotation, etc. This network with such heterogeneous information was drastically different from the "physical" interaction network in this study, and our analytical approaches were different as well. Therefore our results provide additional insight into this disease. (**3**) Regarding comparisons with BrainSpan data, and with those derived from this dataset (Parikshak et al, Willsey et al, and/or Liu et al), we agree with the reviewer that these datasets did not include the corpus callosum and therefore a simple comparison cannot reveal much useful information. However, even though we used different datasets in our study, our conclusion was still in line with those based on the BrainSpan data, especially with Parikshak et al,. Our observations also supported the role of interhemispherical disconnection in ASD by disrupting callosal projecting fibers. This was also the point the reviewer asked us to emphasize in the manuscript (the reviewer's comment #7 above). So we now specifically highlighted this comparison in our discussion as well. (**4**) With all these, we did not further compare other datasets involving gene expression in the corpus callosum, since we had our own independent validation, where we have performed deep RNA-Sequencing (~200 million reads for each sample) for different brain regions (neuronal regions and the corpus callosum). Presumably these datasets will provide much higher resolution than existing dataset mostly based on microarray platforms. Our RNA-seq showed remarkable consistencies with our observation initially made from the Allen Brain Atlas, and our conclusions were also supported by our small-scale IHC study, and also mouse knockout experiments. All these independent genomic datasets lead to mutually supporting results. As is customary for our laboratory, all of these datasets will be publically available. The comparisons with other related studies are now presented in our Discussion section on pg. 21-22.

17 - Module #2 seems very interesting. It appears as if some de novo variant affected genes are in this module, and it could be useful to further study or at least compare with previous work cited here - otherwise the focus on module 13 seems a little arbitrary. The authors note that module #2 is less significant than module #13, but it is also 10x larger than the module 13, which certainly could affect enrichment scores. Also module 13 is enriched for synaptic function, which has been studied extensively and thus the relative enrichment may at least partially reflects this bias. Why is this #2 module so big - does it actually correspond to multiple true modules that reflect different aspects of tissue or developmental time co-expression? For instance since PPI is typically defined outside of cell or tissue or developmental time context, might this large PPI module (module 2), really reflect multiple distinct in vivo functional modules that could be disentangled by looking at tissue and developmental time specific co-expression based on RNA?
In the same vein, the separation of the transcription factor and synaptic functions into distinct modules does coincide with O'Roak et al. and some of the other published studies cited above. All of the enrichment and comparisons are done with module 13 and it would be important to at least include those analyses for module 2 in supplemental tables.

Our response: We thank the reviewer for this comment. (**1**) In the revised manuscript, we have now connected our findings with previous studies, and in Discussion explicitly mentioned that the separation of the TF and synaptic modules coincide with O'Roak et al. See below (see pg. 19-20 in text)

> *"In particular module #2 (with GO enrichment for gene regulation) and #13 (with GO enrichment for synaptic transmission) showed statistically significant enrichment for ASD genes. Their enriched functional categories are consistent with earlier studies for de novo mutations associated with ASD (Ben-David & Shifman, 2013; O'Roak et al, 2012).*

(**2**) The relatively large size of module #2 was due to the dense connections between transcription factors or chromatin remodelers, whereas their connections were sparse outside of this module. The particular pattern was unlikely to be caused by the experimental issues mentioned by the reviewer (e.g. the difference between in-vitro and in vivo results) because such a systematic bias would not have specifically affected TFs only. (**3**) In fact, such extensive interactions among TFs reflects the cooperative nature of TFs for transcriptional regulation. For example, in our recent Cell paper, we identified 207 physical interactions among 26 TFs in K562 cells (Xie, D., et al. Cell, 2013), and such extensive interactions were not expected for randomly sampled genes from the global network. Therefore TFs forming a highly inter-connected network is expected. (**4**) For the implication of this TF module in ASD, the reviewer asked why the enrichment signal was weaker than the synaptic module 13. The reason was not because the synaptic genes in module 13 have been extensively studied, but because only a subset of TFs in the TF module showed clear relevance to ASD. So the overall enrichment signal will be attenuated if we consider all TFs in the module as a whole (albeit still significant). So unlike module 13, where comparisons were done on all the modular genes, further studies on this TF module is all dependent on identifying this set of ASD-associated TFs. We are now working to specifically characterize the ASD-associated TFs in this module, where all these analyses will be performed. To perform this, we have developed a new set of analytic approaches, and generated independent sets of genomic data, which not only supported our observations on this TF module, but also allowed us to identify novel ASD-associated TFs, followed by extensive validation using mouse knockouts. (**5**) In this manuscript we just wish to show the overall trend of the TF module, leaving more detailed characterizations and validations to a subsequent study. There is already a massive amount of effort in the present manuscript, just identifying and characterizing module #13, which the reviewer seemed to recognize.

Minor points:

18. *Figure 1 would be improved by including Module #13's enrichment not just for the SFARI genes, but also for some of the other gene sets queried*

Our response: We thank the reviewer for this comment, and we have now made changes accordingly, and in the new Fig. 1, we now used different colors to label the SFARI genes and genes

in this module associated with ASD-associated de novo CNVs, which presumably have greater effect size.

19 *Figure 2A is difficult to interpret - what exactly are the authors trying to show? Perhaps it would help to show where genes in other modules cluster on this plot, which would contrast how these genes cluster in this feature space compared to other genes?*

Our response: We thank the author for this comment. Now in the revised manuscript, we have made further clarification for this figure panel in the text.

20 -Citations are lacking for "two independent studies" that have implicated the upper and lower cortical layers in autism

Our response: We thank the reviewer and now have added the missing citations.

21- Data sharing: In the supplemental tables, the authors should provide a table containing each protein and the module that contains it. This information will allow others to use the network in future work for comparison and further validation. In supplemental data, the authors should include the exact protein interactions used in the study from BioGRID. These are easily done but essential steps for future usability of these analyses. Finally, the RNA-seq data used here should be made available via SRA and GEO.

Our response: The BioGrid data are now provided (the 2nd datasheet of Supp. File S2) , and can also be downloaded from BioGrid website. RNA-Seq data are submitted to GEO as well.

22 *A more descriptive title emphasizing the relationship to the corpus callosum would be helpful*

Our response: The reviewer raises and interesting point and after much discussion, we prefer the existing title which we feel better reflects the comprehensive nature of our study.

23 *-Introduction: The statement that the known mutations "explain" 10-20% is not really clear. They may be found in that percentage of patients, but given reduced penetrance that has been demonstrated for many, including major gene disrupting CNV, they explain much less of the known genetic variance contributing to ASD.*

Our response: We agree with the reviewer, and it is true that when taking into account the reduced penetrance among patients, the actual percentage should be significantly lower. Given the difficulty in precisely estimating the exact percentage, we have now re-worded the sentence (paragraph 1 in pg. 3 in text), and also see below –

> “These mutations account for very few autism cases, suggesting that the genetic architecture of autism is comprised of extreme locus heterogeneity(Abrahams & Geschwind, 2008).”

24 *-The schizophrenia overlap in module #13 is somewhat glossed over (page 7). This is very interesting and should also be emphasized, as it is an emerging area of interest to the field. A difference in enrichment percentage may just reflect that more ASD genes are known with higher certainty than SZ genes.*

Our response: We thank the reviewer for this comment, and now we have highlighted this observation in Discussion. It is possible that more ASD genes are currently known than SZ genes; however, in our study, if this difference has contributed to the increased ASD enrichment signal towards our module, enrichment in our module would have been expected of these “additional” ASD genes in our knowledge relative to SZ genes. Since both ASD and SZ are synaptic diseases and this module is just newly uncovered in our study, we do not see such a hypothetic scenario is plausible on the basis of our current understanding of both diseases.

Nevertheless we agree with the reviewer that it is interesting that the shared molecular basis between ASD and SZ genes were also recapitulated in our study. In this paper we have provided multiple

lines of evidence supporting the role of this module in ASD, and its potential involvement in other neurological diseases awaits further examination in future.

25 - Overall perspective: On the whole, this is an important study that extends our understanding of the molecular mechanisms that contribute to autism. My points above related to a) bias and b) comparability and overlap with other published studies should be addressed to give readers more confidence and better context for this work. But, despite my detailed comments, these additional analyses should only require minor revisions, mostly related to writing and emphasis.

Our response: We sincerely thank the reviewer for these insightful comments, which have substantially improved our manuscript.

Reviewer #3:

Li et al. in their paper, "Integrated systems analysis reveals molecular network underlying autism spectrum disorders", carry out a series of integrative analyses and experiments over a diversity of data to uncover a subnetwork that is enriched for genes that harbor mutations that associate with ASD and that confirm processes and brain regions involved with ASD, but also uncover novel insights (both process, tissue type, and genes) into ASD. This manuscript addresses an important problem in modern disease research where many different types of data are being generated but then few groups really trying to integrate all such data to come up with comprehensive characterizations of disease. I think the authors strike a nice balance between leveraging very extensive existing data on disease and non-disease conditions and generating their own data and carrying out validation experiments. However, my main struggle with the paper is that it involved a long series of steps that more or
less depended on one another to get to the claims made around module 13 and its role in ASD, but the results within these steps were not always clear or convincing on the statistical side, so that when one considers the propagation of error through the various steps, it is not clear how strongly supported the claims are. I've tried to highlight these issues in the specific comments below.

Our response: We thank the reviewer for all these thoughtful comments, and we now addressed them below.

Specific Comments:

1. The approach taken by the authors while perhaps novel for autism is similar in spirit to network-based approaches taken over the last 5+ years. For example, in Plos Genetics e1000932 (2010) coexpression networks were constructed from different tissues and coexpression modules were identified and then novel loci were identified given modules identified as enriched for loci associated with disease in genome-wide association studies. Here protein interactions are used instead of gene expression and sequencing in place of GWAS, but again similar in spirit. There are a number of other instances of this across different diseases. It may be worthwhile putting this present work in context of what has been done already in different disease areas to help motivate why the particular choices made in this paper were made. For example, why protein interaction instead of gene expression? Protein interactions are typically not assayed in any relevant disease context, they are not scored in a population context, and so on, whereas gene expression is so assayed. On the other hand protein interactions are certainly critical to cell function, defining important mechanisms.

Our response: We thank the reviewer for this question, and we agree that this is indeed an important question to clarify. (**1**) In the revised manuscript, we have now made further clarification in both Introduction and Discussion sections. In particular, as requested by the reviewer, we have now significantly re-written our Introduction section to include most relevant literature (pg. 3-4), which will help place our study in the context of previous studies and also we have now highlighted the motivation underlying this study. (**2**) In Discussion, we have now provided comparisons with previous studies based on different approaches (including those based on co-expression analysis, pg.

21-22 in text). (**3**) We studied protein interactions rather than co-expression network as in previous studies because we aimed to uncover "physical" pathways underlying this disease. Although co-expression networks are likely enriched for interacting genes, information contained in a co-expression network is highly heterogeneous, also reflecting co-localization and co-evolution, etc. Thus, co-expression characterizes "functional co-association" between genes, in which protein interactions only account for a minimal portion. For example, we re-examined expression data from Allen Brain Atlas and computed pair-wise expression correlation for 20803 genes across 295 brain sections. With a threshold of Pearson's R>0.7, we identified 2,810,870 co-expressed gene pairs, whereas only ~70K protein interactions have been experimentally identified so far (accounting for 2.5% of the co-expressed genes), and approximately ~260K (~9% of the co-expressed genes) for the complete human interactome (Rual, J.F. et al. *Nature*, 2005). Thus, we used the protein interaction network to study physical organization between genes. (**4**) As we have shown in the manuscript, studying the protein interaction network can provide novel insights that were not observed from previous co-expression analyses. For example, as asked by Reviewer #2, Ben-David et al. 2012 analyzed co-expression network based on the Allen Brain Atlas data, and identified 2 modules highly expressed in the corpus callosum, but none was associated with ASD. However, with our protein interaction network analysis, we identified a different module for the corpus callosum, whose members are strongly associated with ASD. (**5**) For the use of protein interactions, Reviewer #2 also asked about the completeness of the current interactome data, and we have given a specific response to these technical issues – i.e. please see our response to Reviewer #2's question #9.

2. The approach proposed by the paper was made more difficult to follow given the figures were cited out of order (e.g., the mean expression correlation of expression traits with corresponding protein interactions was cited as supplementary figure 1 in the supplementary information, when in fact it is given as supplementary figure 2). In addition, the methods section is a bit of a mess. It appears to be just a series of thoughts strung together in sentences that are in a single paragraph but that span completely different analyses carried out. Maybe it was intended to be a summary, but it was very, very difficult to follow (e.g., the first several sentences talk about the network that was built, but then the transition from talking about the algorithm applied to build the network and the expression of genes in the Allen Brain Atlas is non-existent and so unclear whether one is talking about the network reconstruction or the assessment of the expression of genes in the module of interest across different brain regions).

Our response: We sincerely thank the reviewer for these comments, and we have carefully revised the supplementary information, and have made better transitions between paragraphs. We have also corrected the order of supplementary figures in our supplementary information.

3. Last sentence, page 4: the authors indicate a "novel parameter-free algorithm" to construct a network based on the protein interaction data, but in the supp information there is a couple of sentence given to describe the approach, referencing papers written 6 years ago on the method. I wouldn't characterize that as a novel algorithm.

Our response: We have now revised this, and have removed the use of "novel" in the text. However, we wish to note that despite the fact that the algorithm was proposed 6 years ago, it has not yet received significant attention for detecting network modules. For example, the best known algorithms for module clustering in protein network is MCL (markov Cluster) and affinity propagation (AP, Frey, B.J. 2007, *Science*), and a recent comparative study on a set of benchmarked datasets has shown that MCL outperformed AP significantly (Vlasblom J. and Wodak, S.J. 2009, *BMC Genomics*). However, both methods have been applied on protein networks from yeast or E.coli (Peregrín-Alvarez, J.M. et al. 2009 *PLoS Comp. Biol.*), but were unsuccessful for the protein interaction networks for human. We now performed additional tests, and on the same human protein interactome used in this study, we observed the algorithm used in our study has achieved 3-fold increase in the modularity score Q relative to MCL and AP, and the identified modules in this study displayed significantly more functional coherence within each topological modules than those from MCL and AP. Given these facts, in our earlier version, we considered the algorithm used in our study was novel, and now, upon the reviewer's comments, we have remove the use of "novel" in our text, and added the comparisons with other clustering algorithms in the Supplementary Information.

4. In the first paragraph on page 6, the authors indicated that module #2 is enriched for genes known to associate with autism, but for this result they do not carry out the permutation testing they carried out for the module #13 result. Why is that? Does module #2 not demonstrate significance under such testing? If so that should be reported.

Our response: We thank the reviewer for this question. (**1**) Reviewer #2 also asked the same question, please see our response to his comment #17. (**2**) Briefly, module #2 was significantly different from module #13, where only a particular set of TFs were strongly associated with ASD, which corresponded to the "unexpected" role of chromatin remodelers in ASD (Ronan, J.L., et al. 2013, "*From neural development to cognition: unexpected roles for chromatin*". *Nature Review Genetics*). Thus across all the member genes, module #2 showed a significant enrichment, but further control experiments/permutation should be differently designed from those procedures in module #13. (**3**) We have a separate study specifically analyzing genes in module #2, including many genomic datasets newly generated to experimentally support this point, and also covering the questions raised by this reviewer. So here we wish to show the overall enrichment signal of this module, leaving more details to our next paper as a natural extension of this manuscript.

5. Throughout when the authors are quoting enrichment statistics from application of the Fisher Exact test, just the p value is being reported without indicating the actual statistic on which the p value is based or without some more intuitive statistic such as the fold-enrichment. Because the p value is correlated with sample size in this test, it is difficult to assess the meaningfulness of the result without knowing the counts and/or the fold enrichment on which the test is based. One could have a 1.1-fold enrichment with a really small pvalue if the sample size is very big, but that would be interpreted very differently than a really small pvalue with a 10-fold or greater enrichment. Even in the tables that report these enrichments, such as supplementary file 1, only FDRs are reported, with no count information, no enrichment statistics, etc.

Our response: We thank the reviewer for this comment, and in this revision we now provided detailed information about enrichment tests in Supplementary Table S1B, where all the information (gene count, percentage and fold-enrichment) has been provided. It is clear that all the enrichment signals reported in this paper were at least 1.7x (from the de novo CNVs).

To assure the reported statistical significance was meaningful, we now included more comparisons for genes affected by the same mutational categories (CNVs, or point mutations) in non-ASD individuals and unaffected siblings. It is now clear that genes affected by spontaneous CNVs or disruptive mutations in ASD probands displayed significant enrichment in our module, whereas those identified from non-ASD individuals did not show any statistical significance, nor did the synonymous mutations in both probands and siblings (Supplementary Table S1B). These sets of additional data and comparisons can be seen in pg. 7-8 in the revised manuscript.

6. On page 6, to support that module #13 is enriched for ASD genes beyond synaptic genes the authors test for enrichment of all non-synaptic genes. However, this would assume that all synaptic genes are 100% known, that the annotations used to identify synaptic genes are 100% accurate and complete. This is unlikely to be true. Couldn't it be that there are many unknown synaptic genes? Given the strong conclusion the authors are attempting to draw from this result, that "the ASD enrichment in module #13 cannot be attributed to only synaptic genes..." this seems an important point.

Our response: (**1**) We certainly agree with the reviewer that not every synaptic gene has been identified so far; however, we feel this comparison is necessary because this set of synaptic genes represent the current status of our knowledge. (**2**) Even if we do not use this synaptic gene list, it is still evident that the enrichment cannot only be attributed to synaptic genes: in Fig. 4 we showed this module overall is involved in oligodendrocyte differentiation and myelination, and these non-neuronal functions are not directly related to synaptic functions; and the corpus callosum itself is a white matter structure devoid of neuronal cells. Thus even if the current synaptic gene list is not complete at this stage, our functional genomic study strongly suggests the implication of this module in ASD cannot be explained by the presumed synaptic bias.

7. At the start of the second paragraph on page 7 the authors restrict to a set of 9,782 genes with CDS and GC content comparable to the module 13 genes. However, in the supplementary info, section 1 of the supp methods that they reference for this gene number, the authors indicate a set of 7,743 genes was identified. The results in the main text and in the supp methods should be made consistent.

Our response: The 9,782 genes in the main text were the background genes with similar CDS length and GC content relative to the genes in the module #13. On the other hand, 7743 genes were of the similar CDS length and GC content with the known ASD genes in literature (SFARI genes). The two gene lists were used for tests of different purposes.

8. In the second paragraph on page 7 the authors give several results reflecting the enrichment of ASD genes identified in human genetic studies in module 13. These results do seem to support the importance of this module in ASD, but there is not much information given to appropriately evaluate this, especially as some of the p values reported are only marginally significant at a 0.05 level. For the de novo and rare CNVs, and disruptive mutations used for these results (referenced in Table S1), it would be nice to have a table of the CNVs, the corresponding genes, some indication of how they were picked, then the genes that are mutated and how "disruptive mutation" were defined. While the reader could go digging through the papers referenced to try and figure this all out, one would still struggle to reproduce the results the authors indicate because the precise count information would be nearly impossible to reconstruct unless explicitly noted by the authors.

Our response: We thank the reviewer for this helpful comment. In this revision, we now provide Supplementary Table S1B, which included all the detailed test statistic and data sources. We now separately listed all the ASD-associated genes affected by different mutational categories, and also genes affected in unaffected siblings (Supporting File S2). Overall, these data were collected from recent publications, whose PMIDs have been separately listed in Supplementary Table S1B. The "disruptive mutations" were identified from the 3 exome-sequencing studies for ASD-related de novo mutations (PMIDs: 22495309, 22495306, 22495311).

9. In second paragraph on page 8, authors indicate they identified 113 nonsynonymous mutations to genes in module 13 and then claim an enrichment of such mutations in this module, but the p value is only 0.021. Given the marginal significance of this enrichment, it is important to understand how many variants were identified overall, what was the exact selection criteria used to identify these variants (some info is given in the supp, but not complete) and then what was the actual expected versus observed counts. Further, the authors are using 1000 genomes as the basis for identifying rare mutations in the set they sequenced, but there are other resources available such as ESP, TCGA, etc. that could give a more comprehensive background estimate if combined.

Our response: We thank the reviewer for this comment. We now have re-analyzed the data based upon Reviewer #2's suggestion for this test. Instead of using the hypergeometric test in our earlier manuscript, we now performed a standard test for mutational burden of the rare nonsynonymous variants in this module. We observed that the module harbors 20% of the nonsynonymous variants found only in our cohort and not previously observed in the 1000 Genome dataset. We then randomly sampled 10000 times from the genome the same number of genes matching CDS length and GC content with the genes in this module, and observed that the random expectation was only 12%±2% ($P=1.2e-3$, and $Z=3.34$). So now we have updated this part in the revised text, and used this test to replace the old test. All other relevant information has been given in our manuscript (pg. 9 in text).

We used 1000 Genomes datasets, and in our earlier study we examined ESP data as well. However, both ESP and TCGA datasets were from diseased individuals. By examining the medical records of our ASD patients, we found that the comorbidities of ASD with other developmental diseases in ESP were not rare, including different types of cancers. Since individuals sampled for 1000 Genomes data were presumably from subjects without known disease, we feel that it better serves the purpose as a control cohort.

© European Molecular Biology Organization

10. I found the second paragraph on page 9 confusing. The results being presented claim replication of the candidate loci ostensibly from the WGS/WES sequencing carried out by the authors on the 29 or cases and controls. Were the variants tested the 113 loci that were localized to module 13? When the authors indicate that the "nonsynonymous variants with greater allele frequency differences between cases and controls...", does this mean such differences were statistically significant? And if so what was the threshold used to declare significance and how was that established? Is the claim that while individual loci are not replicating, that the enrichments for the genes in the module are replicating? I think the authors need to be far clearer on what their primary hypothesis being tested is, how they are testing the null hypothesis corresponding to this, and why the test they are using is valid for this hypothesis. As it stands it appears the authors are using nominal significance thresholds (like 0.05), performing many individual tests, then performing tests on the results of those tests, and not really empirically estimating the null distribution through that entire process but rather doing some permutation here and there to support the results. It does appear there may be something really interesting here, but there are just too many missing details to see it.

Our response: We thank the reviewer for this comment, and we apologize if we did not make this clear in our manuscript. Upon the reviewer's comments, we have now significantly re-written this part and have much improved the clarity especially on the point raised by the reviewer. We now briefly answer the question raised by the reviewer. (**1**) The validation test was to test whether genes identified through our WGS/WES were also likely to have variants with imbalanced allele distribution between cases and controls. So we were not testing the 113 loci, but testing the genes in this module, and the reviewer is correct that genes in the module are replicating, but not individual loci. This is expected given the increased mutational stochasticity in ASD patients (estimated from recent de novo exome-seq studies) and also the well-known genetic heterogeneity of ASD. (**2**) An earlier study analyzed this validation dataset, but could not identify significant signal if testing mutated locus or genes individually (Liu, et al. PLoS Genet. 2013). This was because the disease contribution from individual loci was too subtle to be detected using a regular GWAS approach based upon a pre-defined significance threshold. Thus, in this study, we simply identified variants in genes in this module with allele frequencies greater in ASD patients than in controls ($\Delta F>0$), and observed that genes whose variants are with greater ΔF were also more likely to be observed in our study, whereas this trend was absent when we randomly sampled the same number of genes from the same background (P=9.5e-3 from 10000 simulations). (**3**) As an integrative study, we performed a number of independent tests for many conclusions using the available genomic data. Briefly we used the hypergeometric test for the overall enrichment (e.g. GO/SFARI enrichment), and Fisher's exact test for differential enrichment between two gene groups (e.g. comparisons of mutational burden between cases and controls). We used Wilcoxon's rank-sum test for testing numerical values between two data groups (e.g. gene expression in OPCs and the myelinating oligodendrocytes). We followed common statistical practice and considered P values or FDRs (when necessary) below 0.05 to be statistically significant. (**4**) To assure the statistical significance, in the revised manuscript, we have now included additional comparisons, where we performed the same analyses on the mutations identified from non-ASD individuals or the unaffected siblings, and confirmed the enrichment signals were only seen from the ASD probands (pg. 7-8 in text). We have now summarized all the comparisons in Supplementary Table S1A and B. (**5**) In addition, to the best of our ability, we have re-analyzed some of the tests, and generated the null distributions to estimate statistical significance, such as the enrichment test for rare variants in this module, where we estimated the null distribution by randomly sampling genes with matched GC content and CDS length (see our response to Reviewer 2's comment #12).

11. Page 10, second paragraph, there are claims on the cluster depicted in Figure 3a of two groups being enriched for tissues associated with corpus callosum and neuron-rich regions, hippocampal formation, but no support is given for this claim. Some kind of enrichment test should be performed to support the claim. I think supplementary figures 8 and 9 kind of start to get at that, but it's not clear from this that whatever labels are on the brain regions being depicted are enriched for genes that are specific to different tissue types. It is true that subsequent experiments carried out like the staining do support the claim, but it is just unclear or at least confusing why the claim could be made in the first place from the clustering.

Our response: We thank the reviewer for this comment. Our claim was based on careful examination of the brain sections clustered in each group (Supplementary File S4). Tissues that co-clustered in Group 1 were mostly white matter structures (devoid of neurons, i.e. the corpus

callosum, and the globus pallidus, etc.), whereas the other brain sections were regions with large number of neurons (regions sampled from prefrontal cortex and hippocampal regions, etc.). Therefore by closely examining every brain section in the clustering heatmap, the separation between neuron-rich regions and glial cell-rich regions were evident. Since tissue-expression in brain is mostly defined by the relative cell type compositions, our conclusion was further validated by our study for brain cell types in mice, where the tissues co-clustered with the corpus callosum displayed expression preference towards glial cells (oligodendrocytes and astrocytes), whereas, as expected, the co-clustered neuron rich regions displayed strong expression enrichment in mouse neurons (P=6.4E-4, Fisher's exact test, Fig. 4B). We thank the reviewer for this comment, and in our revision, we now have clarified this point, starting with careful examination of tissue properties and followed by our experimental support from staining and analysis of mouse brain cell types.

12. On page 14 the authors carry out RNAseq on brains from individuals with autism and test reproducibility of the expression data by sequencing multiple samples from the same brain. The correlation results depicted in supplementary figure 12 are used to argue high degree of reproducibility. While the correlations are very high, it would be of interest to carry out the same correlations across the different samples, both within the cases and between cases and controls, just to highlight that such correlation is driven by high intra-individual reproducibility that you do not see in "unmatched" samples.

Our response:

In our earlier manuscript, we showed high correlation of the 2 replicates of brain samples from the same individual. We had two biological replicates (rep. A and B) for the samples, each dissected from different tissue blocks in the same brain region, and the experiments were performed by different individuals Following the reviewer's comments we computed expression correlations between rep. A and rep. B for any pairs of samples. Gene expression for the same individuals indeed displayed substantially higher correlation than any other "unmatched" sample pairs (the median correlation is 0.95 for samples from the same individuals, and the median correlation is 0.89 for samples from different individuals, P=4.4e-3, Wilcoxon ranksum test). Therefore, with this we concluded that the high expression correlation in Supplementary Fig. 12 was indeed due to high intra-individual reproducibility. In the revised manuscript, we added this comparison in the text. See below (pg. 15 in text) –

> *"…The biological replicates produced highly reproducible results with a median Pearson's coefficient equal to 0.95 (range 0.9-0.96; Fig. S12), whereas the correlations among samples from different individuals were substantially lower (median correlation coefficient 0.89, P=4.4e-3, Wilcoxon ranksum test), demonstrating the high intra-individual reproducibility of our technique."*

2nd Editorial Decision 17 November 2014

Thank you again for submitting your work to Molecular Systems Biology. We have now heard back from the three referees who agreed to evaluate your manuscript. As you will see from the reports below, the referees are satisfied with the modifications made and they think that the study is now suitable for publication.

- Please include all relevant information (i.e. accession numbers, the database in which the data has been deposited) regarding the availability of the newly generated RNA-Seq data and exome/whole-genome sequencing data in the "Data Availability" section of your manuscript.
- Additionally, we would be grateful if you could name the institutional body responsible for the approval of the experiments involving human subjects and include this information in Materials & Methods (Human Subjects). Please include a statement that informed consent was obtained from all subjects and that the experiments conformed to the principles set out in the WMA Declaration of Helsinki and the Department of Health and Human Services Belmont Report.

Reviewer #1:

The authors have comprehensively revised their initial manuscript, carefully taking into account the large number of detailed comments from the three reviewers. Altogether, this has resulted in a much clearer and stronger manuscript worth publishing in MSB.

As far as my comments are concerned, all except one (see below) have been addressed properly, relevant changes have been introduced in the revised version, and other issues (some of which raised by the other reviewers, e.g. discussion of module #2) have been discussed in the detailed responses provided in a convincing manner. By themselves, they would be worth a complementary commentary by a domain expert.

As for the comprehensiveness and seriousness of the reviewing process, the length of the reviewers comments and the authors responses speaks for itself: with 33 pages, it is now longer than the main text of manuscript (22 pages without counting the references and of course the wealth of supplementary material). In many respect, this could be used as a a tutorial for future referees and young scientists.

My remaining concern is about the Abstract, which still does not give full justice to one of the strong point of the approach used (also emphasized positively by the other reviewers) that the authors have managed to find an efficient balance in leveraging previously existing and newly generated data. They argue that the allowed word count of the Abstract (175) does not allow them to mention this. However, it only takes a few words to say that tey "integrated previous and newly generated data", and in any case since the current word count of the Abstract is 186, it would have to be revised, or they should be allowed to go up to 200 to include the much needed and valuable statement.

Reviewer #2:

The authors have done a very comprehensive and thorough job of responding to all of our critiques and those of the other reviewers. This paper is an excellent contribution to the literature and should be published in MSB.

Reviewer #3:

I have reviewed the revision made by the authors, their responses to my original comments, as well as the comments of the other reviewers and the author comments to those reviewer comments as well. The authors have made very significant revisions to their manuscript to address all of the reviewer concerns. I believe the authors have adequately addressed all of the reviewer comments, which were very extensive, and as a result the manuscript is significantly improved. I do not have additional significant criticisms.

2nd Revision - authors' response 27 November 2014

Thank you for your information, and we are glad to know that all the reviewers are now satisfied with our revised manuscript.

- We have now provided all the data accession numbers (GEO and SRA) in the section of Data Availability.
- In your email, you asked us to name the "institutional body responsible for the approval of the experiments involving human subjects". We have communicated with our IRB, and it was determined that our study was exempt from an IRB review since only the post-mortem brain tissues were examined in this study, which were initially collected from the de-identified and deceased individuals by the brain banks. Our IRB determination letter is available upon your request. The section of Human Subject has been added to the Methods and Materials.
- We now followed the Reviewer #1's comment and added a sentence in the abstract, stating that we used previously and newly generated data to build an integrative framework.
